# Quantitative multiorgan proteomics of fatal COVID‐19 uncovers tissue‐specific effects beyond inflammation

**DOI:** 10.15252/emmm.202317459

**Published:** 2023-07-31

**Authors:** Lisa Schweizer, Tina Schaller, Maximilian Zwiebel, Özge Karayel, Johannes Bruno Müller‐Reif, Wen‐Feng Zeng, Sebastian Dintner, Thierry M Nordmann, Klaus Hirschbühl, Bruno Märkl, Rainer Claus, Matthias Mann

**Affiliations:** ^1^ Department of Proteomics and Signal Transduction Max Planck Institute of Biochemistry Martinsried Germany; ^2^ Pathology, Medical Faculty University of Augsburg Augsburg Germany; ^3^ Hematology and Oncology, Medical Faculty University of Augsburg Augsburg Germany; ^4^ Present address: Department of Physiological Chemistry Genentech South San Francisco USA

**Keywords:** COVID‐19, mass spectrometry, pathology, proteomics, virus, Microbiology, Virology & Host Pathogen Interaction, Proteomics, Respiratory System

## Abstract

SARS‐CoV‐2 may directly and indirectly damage lung tissue and other host organs, but there are few system‐wide, untargeted studies of these effects on the human body. Here, we developed a parallelized mass spectrometry (MS) proteomics workflow enabling the rapid, quantitative analysis of hundreds of virus‐infected FFPE tissues. The first layer of response to SARS‐CoV‐2 in all tissues was dominated by circulating inflammatory molecules. Beyond systemic inflammation, we differentiated between systemic and true tissue‐specific effects to reflect distinct COVID‐19‐associated damage patterns. Proteomic changes in the lungs resembled those of diffuse alveolar damage (DAD) in non‐COVID‐19 patients. Extensive organ‐specific changes were also evident in the kidneys, liver, and lymphatic and vascular systems. Secondary inflammatory effects in the brain were related to rearrangements in neurotransmitter receptors and myelin degradation. These MS‐proteomics‐derived results contribute substantially to our understanding of COVID‐19 pathomechanisms and suggest strategies for organ‐specific therapeutic interventions.

## Graphical abstract

This study reports a proteomic investigation of fatal COVID‐19 across organs using mass spectrometry, highlighting the central role of circulating inflammatory molecules and uncovering tissue‐specific alterations beyond inflammation.

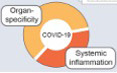

**The paper explained**

**Problem**

COVID‐19 causes profound systemic inflammation as well as severe organ damage in fatal cases. Despite extensive research, how it affects different body tissues is not fully understood. While comprehensive studies have been conducted, they often overlook the role of proteins (the proteome) in this disease.

**Results**

We analyzed the postmortem proteome of an autopsy cohort of the early pandemic phase to investigate the tissue‐specific effects of COVID‐19 beyond inflammation. To enable fast and streamlined processing of hundreds of formalin‐fixed paraffin‐embedded (FFPE) tissue samples, we developed a parallelized protocol for mass spectrometry‐based proteomics that reduced workflow complexity and enabled the direct identification of SARS‐CoV‐2 peptides in the lungs. Our analyses recapitulate the predominant role of the systemic‐inflammatory response to the viral infection across organs but separated it from the organ‐specific effects. In the lungs, the proteomic signature of COVID‐19 was closely related to pathologically similar diffuse alveolar damage. Direct and indirect effects in the kidneys, liver, and lymphatic and vascular systems highlighted multi‐organ damage as well as potential damage pathways in the brain.

**Impact**

Our study improves understanding of how COVID‐19 damages different body tissues, going beyond general inflammation. Moreover, our workflow and analysis strategy allow parallelized and high‐throughput processing of FFPE tissue in clinical contexts and suggest strategies for organ‐specific therapeutic interventions.

## Introduction

The global SARS‐CoV‐2 pandemic has engendered a tremendous research effort aimed at understanding virus biology, intervening in its spread and finding therapeutic modalities. Current literature encompasses clinical observations, case studies, diverse cell culture, or animal models, all of which have greatly expanded our knowledge (Huang *et al*, 2020; Sadarangani *et al*, 2021; Jackson *et al*, 2022). Among these efforts, omics technologies have also crucially contributed to an unbiased, system‐level view of the disease (Overmyer *et al*, 2021; Stephenson *et al*, 2021). Apart from genomic sequencing of virus variants and patients with genetic predispositions (Lu *et al*, 2020; Initiative, 2021; Pairo‐Castineira *et al*, 2021), virus and host transcriptomics has added a more holistic understanding of the infection process and its consequences (Daamen *et al*, 2021; Melms *et al*, 2021; Kim *et al*, 2021a).

Proteomics studies gene expression at the level of functional cellular units and therefore directly measures biological functions (Aebersold & Mann, 2016; Zhu *et al*, 2021). However, due to technological challenges, it has been applied much less frequently than other omics approaches. Mass spectrometry (MS)‐based proteomics has enabled the investigation of the interactome between viral and host proteins, revealing the potential roles of SARS‐CoV‐2 proteins and suggesting host factors and processes for therapeutic intervention (Gordon *et al*, 2020; Stukalov *et al*, 2021). As they were performed in cell lines, these studies do not directly reflect *in vivo* conditions and do not address tissue‐specific aspects of the disease. Proteomics has also successfully been employed to identify biomarkers in plasma or serum to predict patient outcomes. Several studies have analyzed a range of SARS‐CoV‐2‐infected patients, including detailed time courses (Messner *et al*, 2020; Geyer *et al*, 2021). Main effects were related to the immune response, specifically the complement system and blood coagulation. Plasma proteomics has also complemented multi‐omics approaches. Interestingly, despite the limited depth of quantification, MS‐based proteomics had the highest predictive power in assessing disease severity (COMBAT, 2022).

By its nature, proteomics of circulating body fluids does not directly report on the effects of infection in different human organs. Tissue proteomics would be an attractive approach to investigate this, but requires patient tissues and the ability to quantitatively analyze large numbers of human tissue samples. A pioneering study from the initial Wuhan outbreak analyzed seven tissues of 19 patients, which clearly highlighted the effects of virus‐induced inflammation and coagulation (Nie *et al*, 2021). These systemic effects dominated the responses in all examined tissues.

Here, we set out to develop a scalable, MS‐based strategy to study the effects of SARS‐CoV‐2 infection on the proteomes of the major human organ systems. Taking advantage of our previous experience in the establishment of MS workflows to process formalin‐fixed, paraffin‐embedded (FFPE) tissue (Wisniewski *et al*, 2013; Coscia *et al*, 2020), we developed a robust and simple workflow for paraffin dissociation, sample lysis, and MS data acquisition in a parallelized manner. This enabled the analysis of a postmortem cohort encompassing more than 350 proteomes representing 10 different organs. We employed proteomic quality control panels (Geyer *et al*, 2019), which enabled us to control for the effect of circulation‐derived proteins in the measured tissue proteomes. With this approach, we identified and deconvoluted the predominant, tissue‐wide contribution of the systemic‐inflammatory response from organ‐specific effects of a SARS‐CoV‐2 infection. In lung tissues, COVID‐19‐induced damage was most extensive, with similarities and differences to other destructive lung diseases. Several tissue types showed a unique signature in response to a SARS‐CoV‐2 infection with the kidney and liver having the most proteomic alterations apart from the lymph and vessel system. We finally uncover secondary inflammatory effects in the immune‐privileged brain involving rearrangement of neuronal receptors as well as a decrease in myelin abundance.

## Results

**Figure 1:**
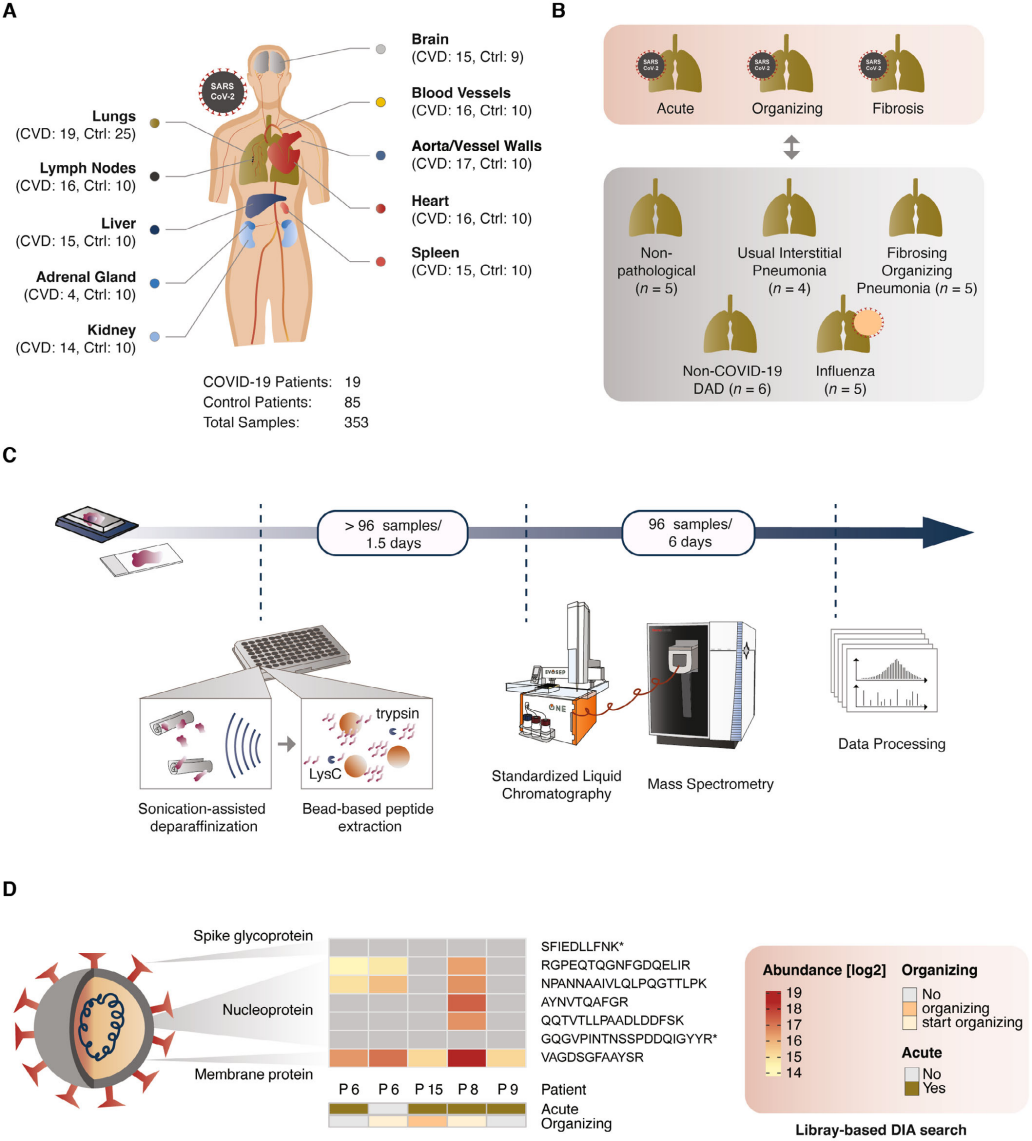
Study design and MS‐based proteomics workflow overview Overview of the cohort and organs included in this study. Specimens of 10 different organs from 19 patients with fatal COVID‐19 (CVD) were analyzed in this study and complemented with an organ‐matched control group (Ctrl) derived from non‐COVID‐19 patients. Control samples did not show a pathology in the respective organ, originated from a heterogenous medical background but comparable age and gender distribution in comparison to the COVID‐19 cohort. Overall, the cohort comprised patients from the COVID‐19 (CVD) and control groups (CTRL) from lung (CVD = 19, CTRL = 25), heart (CVD = 16, CTRL = 10), mediastinal lymph nodes (CVD = 16, CTRL = 10), blood vessels (CVD = 16, CTRL = 10), large vessel (aortal) walls (CVD = 17, CTRL = 10), brain (medulla oblongata, basal ganglia; CVD = 15, CTRL = 9), liver (CVD = 15, CTRL = 10), spleen (CVD = 15, CTRL = 10), kidney (CVD = 14, CTRL = 10) and adrenal glands (CVD = 4, CTRL = 10). A total of 353 samples were acquired from 19 COVID‐19 patients and 85 control patients.For specimens of COVID‐19 in the lungs, tissue was sampled from regions histologically classified into acute, organizing, and fibrotic stages of COVID‐19 where possible. Next to nonpathological (tissue with healthy phenotype) control tissue (patients *n* = 5), these samples were compared to non‐COVID‐19 pathologies of the lungs including influenza (*n* = 5), non‐COVID‐19 diffuse alveolar damage (DAD, *n* = 6), common interstitial pneumonia (UIP, *n* = 4) with progressive fibrosis of the lung, and fibrosing organizing pneumonia (OFP, *n* = 5).Schematic representation of the high‐throughput and standardized proteomic workflow developed in this study. Starting from paraffinized tissue, samples were prepared by the integration of focused sonication and bead‐based aggregation in a 96‐well format, and analyzed by LC–MS/MS using standardized gradients in single‐runs per proteome (15 samples per day).Identification of peptides from diverse components of SARS‐CoV‐2 in the lungs confirmed by a library‐based approach. Relative abundances of peptides in each patient sample denote the presence (heat map color scale) or absence (gray) of signal in the respective sample. Annotated peptides by asterisks (*) were identified in the proteomic libraries only. The identification of these peptides in directDIA mode is shown in Fig EV1E.

Our postmortem cohort was derived from autopsies at the University Medical Center Augsburg from April to May 2020 involving 19 patients with a fatal course of COVID‐19 (Schaller *et al*, 2020). All patients had tested positive for SARS‐CoV‐2 by nasopharyngeal swabs, and viral RNA was quantified by RT‐qPCR (Hirschbühl *et al*, 2021). Patient ages ranged from 57 to 90 years and included 74% male and 26% female patients. For each of the COVID‐19 patients, we histologically assessed FFPE specimens from 10 different organs. A cohort of healthy control tissues was selected from non‐COVID‐19 patients from the FFPE sample archive of the Department of Pathology at University Hospital Augsburg for each organ, respectively. Tissue of the control cohort did not show a pathology in the selected organ, while additional samples were collected for the lungs from phenotypes with similarities to COVID‐19 such as influenza and non‐COVID‐19‐related DAD. Overall, control samples originated from a heterogenous medical background but comparable age and gender distribution to the COVID‐19 cohort. The resulting cohort included patients from the COVID‐19 and control groups (patient *n* = CVD/CTRL) from lung (*n* = 19/25), heart (*n* = 16/10), mediastinal lymph nodes (*n* = 16/10), blood vessels (*n* = 16/10), large vessel (aortal) walls (*n* = 17/10), brain (medulla oblongata, basal ganglia; *n* = 15/9), liver (*n* = 15/10), spleen (*n* = 15/10), kidney (*n* = 14/10), and adrenal glands (*n* = 4/10) and selected them for proteomic analysis (Fig 1A, Table EV1). The additional control group of the lungs comprised different types of phenotypically similar non‐COVID‐19 lung diseases including influenza (*n* = 5), non‐COVID‐19 diffuse alveolar damage (DAD, *n* = 6), common interstitial pneumonia (UIP, *n* = 4) with progressive fibrosis of the lung, and fibrosing organizing pneumonia (OFP, *n* = 5; Fig 1B). To account for different pathological phenotypes within one specimen, several tissue regions were collected for COVID‐19 and non‐COVID‐19 DAD controls. Samples were processed for each tissue separately to allow proteomic stratification within each organ as well as an organ‐wide relative assessment for COVID‐19 and control samples (Appendix Fig S1A), including the extended control cohort of the lungs (Appendix Fig S1B). Altogether, this resulted in more than 350 human tissue proteomes from 19 COVID‐19 and a heterogeneous group of 85 control patients to be analyzed in a robust, quantitative and reproducible manner (Dataset EV1). Patient characteristics including BMI, smoking status, and comorbidities are listed in Tables EV2 and EV3 for COVID‐19 patients, while detailed information on control cases is given in Dataset EV2 and Tables [Link], [Link].

### Development of a streamlined tissue proteomics workflow

Recent efforts in the acquisition of proteomic data in a high‐throughput format have aimed at simplifying and streamlining the chromatographic setups (Bache *et al*, 2018; Bian *et al*, 2020; Messner *et al*, 2021; Gao *et al*, 2022). Facing the need for a rapid and universal strategy, we wanted to build on these concepts as well as previous work that allows the preparation of pathological tissue samples for direct MS analysis (Jiang *et al*, 2007; Wisniewski *et al*, 2013; Foll *et al*, 2018; Coscia *et al*, 2020; Muller *et al*, 2020). Briefly, to enable parallelized, nontoxic paraffin dissociation and sample lysis, we integrated protein aggregation capture (Batth *et al*, 2019; Hughes *et al*, 2019) with sonification‐based and heat‐assisted deparaffinization of archived FFPE tissue. Our new protocol allows all steps to occur in one well of a 96‐well plate, does not use toxic chemicals and would also be suitable for nonparaffinized tissue and complete automation. Upfront of MS analysis, we used a standardized liquid chromatography (LC) system to reduce measurement complexity while keeping high reproducibility (Bache *et al*, 2018), using data‐independent acquisition (DIA) on an orbitrap analyzer. In combination, we found this approach to provide an appropriate methodology for a large‐scale translational project based on pathologically preserved FFPE tissue proteomes while reducing the time of active tissue handling, improving reproducibility and promoting robustness (Fig 1C, Materials and Methods section).

**Figure EV1:**
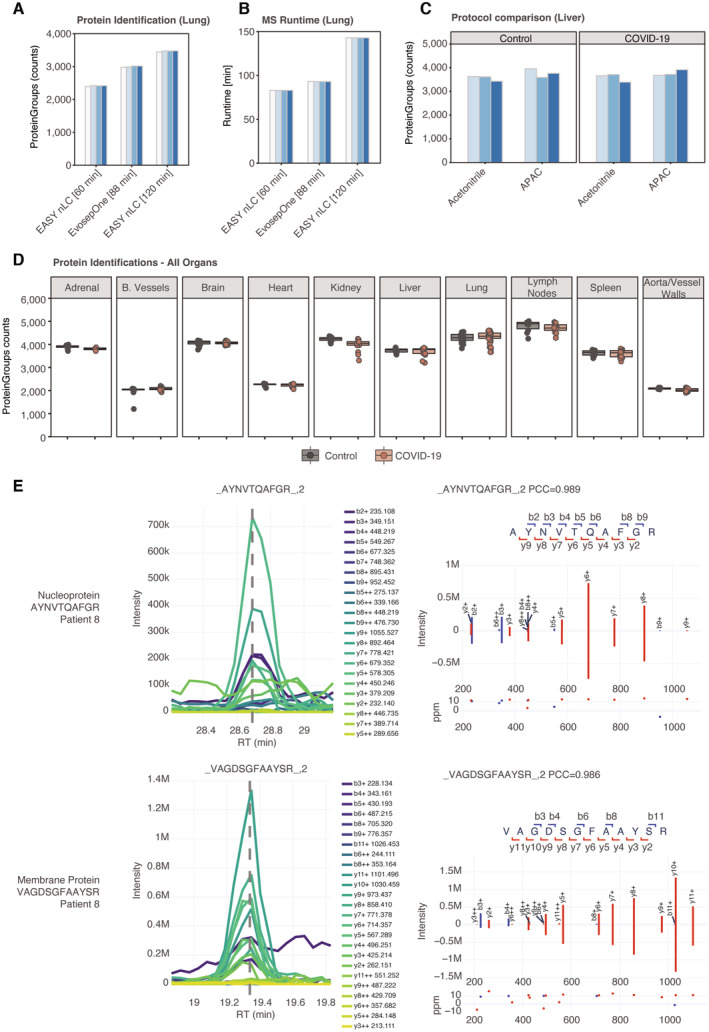
Mass spectrometry quality control Comparison of protein identification in lung tissue of the COVID‐19 cohort between the standardized 88 min gradient on Evosep One and a 60 or 120 min gradient on the EASY nanoLC system, respectively. Bars represent tissue samples of different patients and were grouped by the employed LC system, respectively.MS runtime of the comparison depicted in (B).Evaluation of our workflow for streamlined deparaffinization and lysis (APAC) and our previous state‐of‐art protocol for the processing of deparaffinized FFPE tissue in a 96‐well format (Coscia *et al*, 2020). Each bar depicts tissue samples of different patients while being grouped by the tested workflow, respectively.Counts of quantified protein groups in COVID‐19 samples and corresponding control groups in all organs using direct DIA data processing (not using a separately acquired DDA library). Each data point depicts a patient‐derived sample as indicated in Table EV1.Extracted ion chromatograms and prediction of fragment intensities for two examples of SARS‐CoV‐2 peptides in the directDIA acquisition mode of the lungs. For both peptides, the same patient was selected to represent the cohort.

We previously measured tissue proteomes with 2 h gradients, whereas the Evosep One system features either 15 or 30 samples per day (SPD), corresponding to 44‐ or 88‐min gradients. For our study, we chose the 15 SPD method as the best compromise of the depth of coverage and throughput. Compared to our previous, slower LC setup, we achieved on average 13% less protein identification at 35% less measurement time (Fig EV1A and B). To also evaluate the sample processing quality of streamlined workflow, we compared it to our previous state‐of‐the‐art protocol for the processing of deparaffinized FFPE tissue in a 96‐well format (Coscia *et al*, 2020) by processing a subset of liver tissues from our cohort both on the same LC and MS instruments. Measured by the identification of protein groups, our greatly simplified protocol achieved equal performance (Fig EV1C).

Finally, we applied our new workflow to acquire a total of 353 tissue proteomes in < 1 month of total measurement time. By using this integrative approach, we overall identified 7,315 proteins in all tissue types and demonstrated robust and repeatable identification levels in the COVID‐19 and the control groups, ranging from about 2,000 different proteins in blood vessels to nearly 5,000 in lymph nodes (Fig EV1D).

### Detection of SARS‐CoV‐2 peptides *in vivo* in human lung tissue

MS‐based proteomics is unbiased in the sense of not being targeted to specific molecules of interest. This should make it possible, in principle, to directly detect and quantify SARS‐CoV‐2 virus proteins, although not reported by a previous study despite extensive fractionation of the samples (Nie *et al*, 2021). After adding virus protein sequences to the search database, we indeed unequivocally identified peptides of the most highly expressed viral proteins. We manually verified our findings by matching our experimental data to predictions of fragment intensities of these peptides and the extracted ion chromatograms at the MS2 level as described recently (Zeng *et al*, 2022; Fig EV1E). The viral proteins were found in several lung tissue samples of four of the subjects, all of whom had either acute or organizing diffuse alveolar damage (DAD) and were among those with the highest viral load as judged by qPCR (Fig 1D; Hirschbühl *et al*, 2021). These SARS‐CoV‐2 peptides were either derived from the membrane (M, UniProt: P0DTC5) or the nucleoprotein (N, UniProt: P0DTC9). Hence, the identification of these viral peptides provided a proof of principle of the depth and accuracy of our FFPE‐based workflow from postmortem tissue. We conclude that the robustness, reproducibility and sensitivity of our workflow are of adequate quality for the purposes of our study.

### The systemic inflammatory response of COVID‐19

**Figure EV2:**
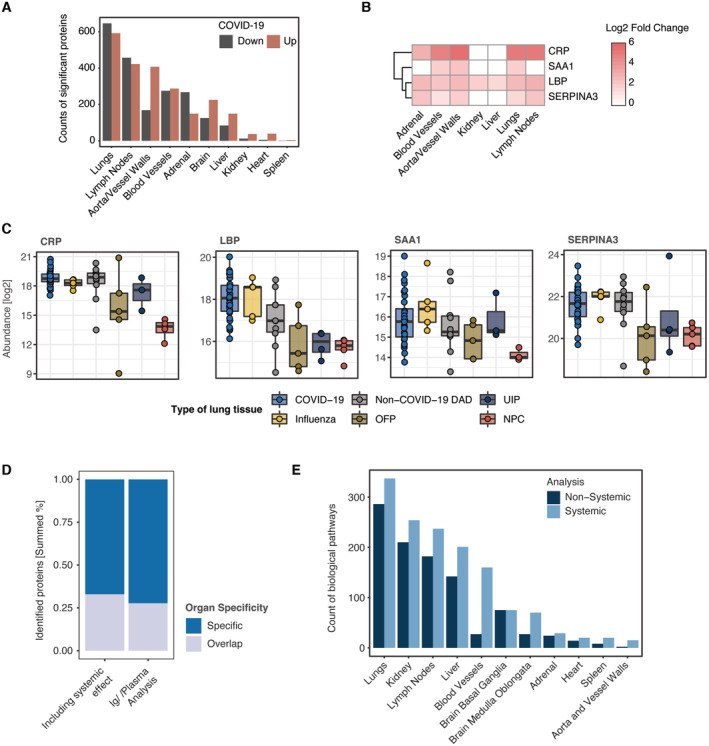
The systemic‐inflammatory response in COVID‐19 Number of significantly differentially regulated proteins (COVID‐19 vs. controls) across all organs (minimum fold‐change 1.5 at a *q*‐value of <0.05).Fold changes (log2) of plasma‐derived markers and predictive proteins for a severe progression of COVID‐19.Boxplot representing the log2‐abundance of the proteins shown in (B) for COVID‐19 and similar pathologies of the lung which have been included into our study. Boxplots represent the 25‐ and 75‐percentile with median values as central band; whiskers span the 1.5‐fold interquartile range. Each data point depicts a patient‐derived sample as indicated in Table EV1.Summed percentage of significantly differentially regulated proteins (minimum fold‐change 1.5 at a *q*‐value of <0.05) for the original proteome and after identification of the systemic effect, calculated for organ specificity and overlap in at least two organs, respectively.Count of biological pathways derived from an enrichment using the Reactome, KEGG and GO biological process databases and shown for differentially regulated proteins with association to the systemic and nonsystemic subset of proteins across all organs.

The clinical presentation of COVID‐19 is highly variable ranging from fever, worsening of the general condition, respiratory tract symptoms to acute lung injury and involvement of other organs, highlighting the systemic nature of the disease. In order to describe these effects at the level of the proteome, we compared the differential protein expression between COVID‐19 patients and respective control tissues across all organs in our study. The proteomic alterations were most substantial in the lungs, in which 25.6% of all proteins significantly changed (minimum fold‐change 1.5 at a *q* value of <0.05, Fig EV2A). The lymphatic and blood vessel systems also had multifold, although fewer changes (17 and 24% of proteins, respectively).

**Figure 2:**
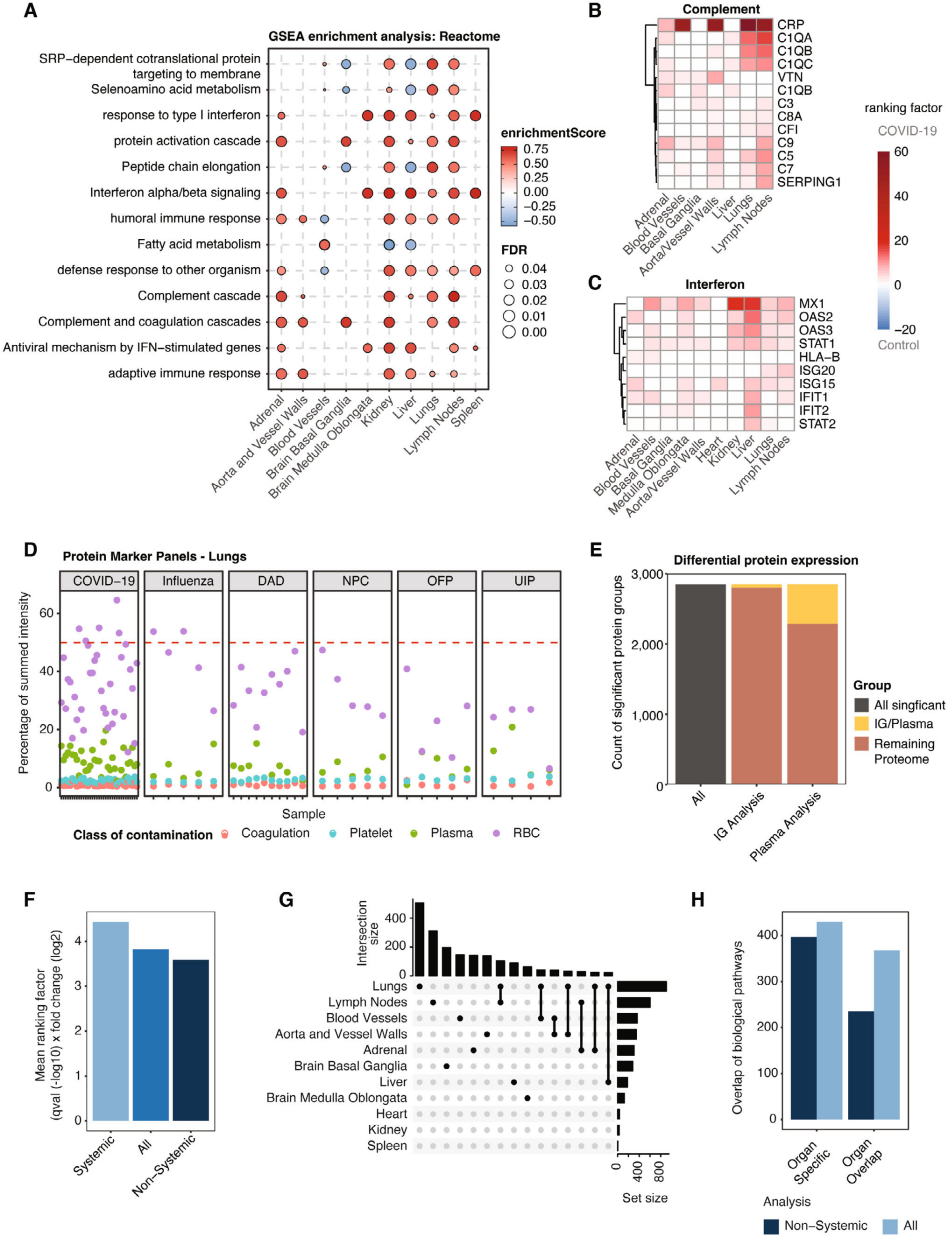
The systemic‐inflammatory response masks tissue‐specific effects ATerms of biological pathway enrichment analysis (GSEA) of the differential protein expression between COVID‐19 and controls and a minimal overlap of six organs in this study using the pathway Reactome database. Color codes of each enrichment term depict the enrichment score, whereas point size reflects the respective FDR.B, CRepresentative protein panels for the biological pathway “complement system” (B) the “interferon signaling” (C) with significance (fc > 1.5, *q*‐val < 0.05) in ≥ 3 organs and 2 organs, respectively. Each heat map depicts the ranking factor (*q*‐value (−log10) × fold change (log2)) for a protein in different organs as a measure of comparable significance.DAssessment of the prevalence for proteins in previously identified quality marker panels on coagulation, plasma, platelets and red blood cells (RBC), exemplarily shown for lung tissue. The percentage of summed protein intensities for each panel is illustrated for each sample across all disease pathologies included in our study. The ratio of 50% signal of the total protein abundance is highlighted (dashed red line).ERatio of plasma or immunoglobulin‐related proteins on the total of significantly differentially regulated proteins (fc > 1.5, *q*‐val < 0.05) between COVID‐19 and control samples across all organs.FCalculation of the mean ranking factor (*q*‐value (−log10) × fold change (log2)) for significantly dysregulated (COVID‐19 vs. control) proteins of the original proteome (All) as well as proteins associated with the systemic and nonsystemic effects in tissue across all organs.GUpset plot depicting the intersection of significantly dysregulated (COVID‐19 vs. control) proteins across all organs.HComparison of a biological pathway enrichment for significantly dysregulated (COVID‐19 vs. control) proteins of the original proteome and after identification of the systemic effects using the Reactome, KEGG and GO biological process databases across all organs. For each enrichment, the number of pathways is shown for organ specificity and overlap in at least two organs.

To characterize the proteome of our tissues with regard to previous reports and known properties of COVID‐19, we performed a GSEA enrichment using the Reactome, KEGG, and GO Biological Processes databases (Mi *et al*, 2019; Gillespie *et al*, 2022; Kanehisa *et al*, 2022) and extracted enriched pathways that showed substantial co‐occurrence across the organs of our study (≥ 6 organs, Fig 2A). Notably, these biological processes highlighted the typical systemic‐inflammatory effects of the disease and suggested that processes such as enhanced coagulation are not primarily of vascular nature. The most prominent of the organ‐overlapping terms was the complement cascade, of which we quantified 13 different proteins that showed dysregulation across seven of 10 different tissue types (Fig 2B). Proteins initiating the cascade, such as Complement C1q subcomponent subunits A, B, and C (C1QA, C1QB and C1QC) were mainly enriched in lungs and lymph nodes. Complement factors C3, C5, C7, C9, in contrast, were commonly upregulated in lymph nodes and aorta/vessel walls.

The second most altered group consisted of components of the interferon pathway that were consistently upregulated in all measured tissue types, including brain, liver, kidney, and adrenal glands (Fig 2C). This included proteins like MX1, OAS2, OAS3, and IFIT1 mediating interferon‐induced and RNA‐specific antiviral activity. Proteins involved in the transmission of type I interferon signaling (STAT1) or the regulation of the IFN‐I response (ISG15) were also significantly higher abundant in several organs.

We noticed that the abundance of acute‐phase proteins CRP and LBP as well as the predictive markers SERPINA3 and SAA1 that had been associated with severe COVID‐19 disease progression in blood and plasma (Messner *et al*, 2020; Geyer *et al*, 2021; COMBAT, 2022), were also clearly increased in the large majority of organs (Fig EV2B). This was also true for the lung diseases used for comparison (Fig EV2C). CRP was upregulated in all disease phenotypes of the lungs compared to control tissue. LBP and SERPINA3 were mainly elevated in lung damage induced by influenza and non‐COVID‐19 DAD. Hence, host systemic inflammation upon SARS‐CoV‐2 infection appeared to be predominant and organ‐spanning in our proteomic tissue screening, recapitulating clinical features previously reported in proteomic tissue studies of COVID‐19 (Nie *et al*, 2021).

### Deconvolution of organ‐specific effects in COVID‐19

Prompted by the ubiquitous predominance of systemic effects in COVID‐19, we next investigated the possible contribution of the circulation‐derived proteome in our tissue‐based study and its impact on data interpretation. For this purpose, we employed proteomic “quality control panels”, which we had previously developed to control for the effect of blood compartment contamination in the proteomes of body fluids (Geyer *et al*, 2016; Karayel *et al*, 2022). We focused on representative markers of panels for coagulation, platelets, plasma, and red blood cells (RBC). This revealed that markers of the RBC panel dominated some specimens of the COVID‐19 cohort and accounted for more than 50% of the summed intensity of the MS signal (exemplarily shown for lung tissue in Fig 2D).

The above results suggested that blood was present in our proteomic samples and contributed significantly to the proteomic profile of the organ samples as expected since residual blood in the preparation cannot be excluded during postmortem processing of human tissue. At the same time, circulation‐mediated effects of the immune system are part of the systemic effects of SARS‐CoV‐2 infection, including tissue destruction. To distinguish these contributions of the blood proteome, we next considered proteins that are constituent and commonly identified parts of plasma proteomes (Geyer *et al*, 2016) including immunoglobulins (in total 1,060 different proteins, Dataset EV3). We termed this the “circulation‐mediated” proteome subset.

Mapping this subset to the significant proteomic alteration between COVID‐19 and control samples across all organs revealed that it accounted for 24.1% of all significantly differentially regulated proteins and 19.9% of which were various immunoglobulins (Fig 2E). To interpret these differences, we considered the ‘ranking factor’, defined as the product of fold‐change and statistical consistency (corrected *q*‐value), for each protein (Xiao *et al*, 2014). The mean ranking factor of the circulation‐mediated subset was substantially higher than the mean of all significantly altered proteins (Fig 2F). The same value of the noncirculation‐mediated subset was clearly decreased, indicating that overall results were indeed influenced by the blood proteome. Based on these findings, we hypothesized that the circulation‐mediated proteome subset might have masked organ‐specific effects in our data interpretation. Interestingly, when separating these effects, the remaining effects of COVID‐19 on the host proteome became more organ specific, with the majority of tissue now exhibiting unique and distinct groups of differentially regulated proteins. As before, the number of differentially regulated proteins with unique changes was most substantial in the lungs, followed by the lymph nodes and the basal ganglia of the brain (Figs 2G and EV2D).

To interpret the impact of the circulation‐mediated effects, we extended the analysis using GSEA pathway enrichment across all organs. Interestingly, the number of enriched pathways decreased markedly in each organ upon data deconvolution (Fig EV2E). The organ specificity of the pathway enrichment remained nearly unchanged. However, we observed a drastic decrease in the organ overlap when considering the noncirculation‐mediated proteome subset (Fig 2H).

Thus, our analysis uncovered a predominant role of the circulation‐mediated effects in the COVID‐19 tissue proteome data. In this regard, we recapitulate the central role of circulation‐derived proteins in COVID‐19 that are related to symptomatic characteristics of the disease such as destructive injury in lungs and other systemic effects during its progression. The quality marker panels that we had previously used to exclude contaminated body fluid samples here proved useful to distinguish organ‐specific from systemic effects. This formed the basis for all subsequent analyses to determine organ‐specific changes in SARS‐CoV‐2 infection after separating out the systemic inflammatory effects, something that had not been done in previous work (Nie *et al*, 2021; Appendix Fig S2A and B).

### Pulmonary manifestations of COVID‐19

**Figure 3:**
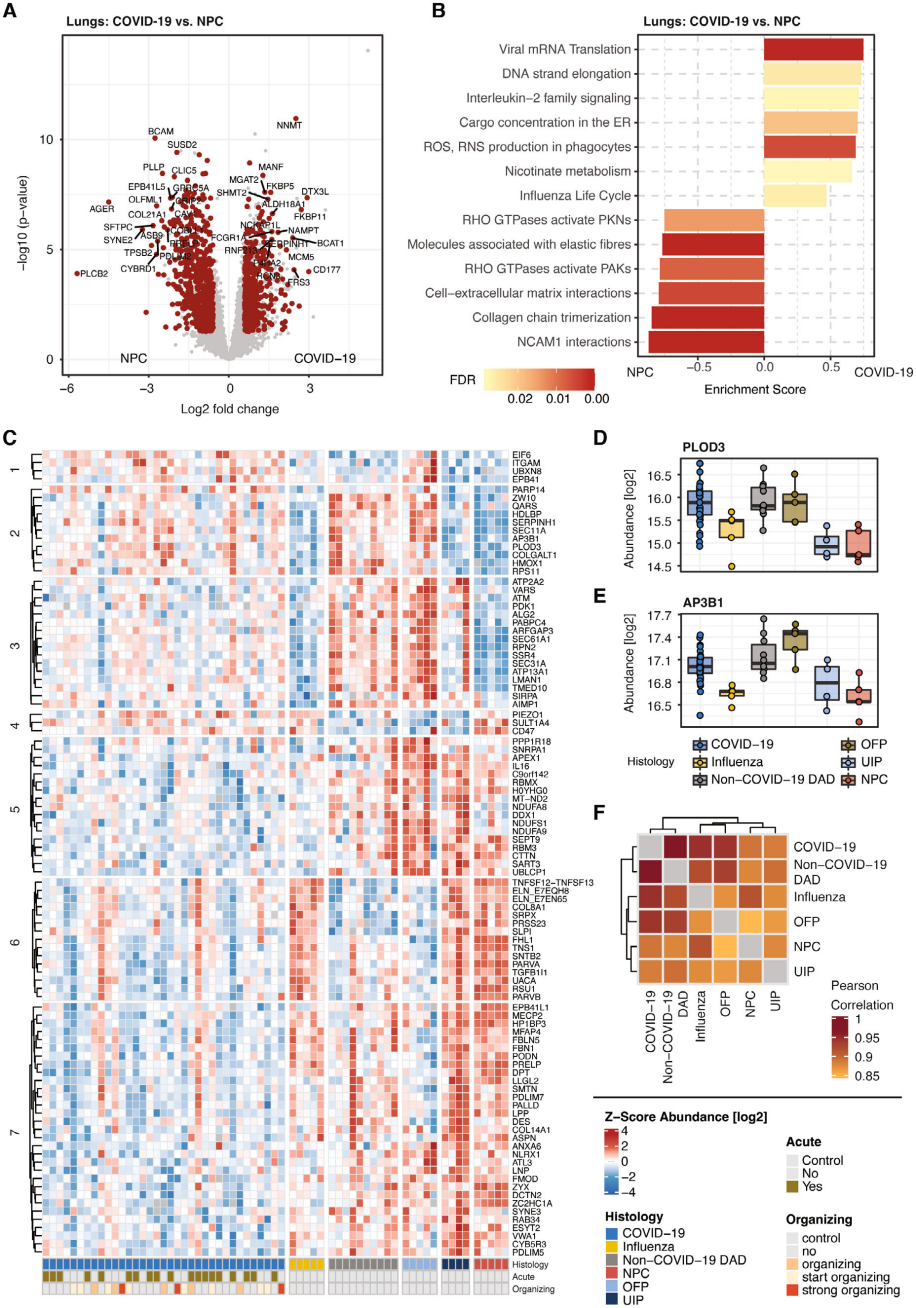
Manifestation in the lungs and classification among related lung diseases ADifferential protein expression between COVID‐19 and nonpathological control samples (NPC). Significant proteins (*t*‐test, *q*‐val < 0.05, fold change > 1.5) are highlighted in red.BGSEA enrichment for the differentially regulated proteins in COVID‐19 and NPCs in (A) using the pathway Reactome database. For each protein subset, the most representative terms are shown.CHeatmap of ANOVA significant (ct < 0.001) proteins that have been filtered for differential regulation by post‐Tukey significance (adj. *P*‐value < 0.05) in at least two lung diseases compared to COVID‐19. Annotations show the groups of lung diseases as well as the disease pathology by acute or organizing tissue architecture (bottom).D, EExemplary depiction of the differentiation of COVID‐19, non‐COVID‐19 DAD and OFP in cluster 2 (C) by the protein abundances of PLOD3 (D) and AP3B1 (E). Boxplots represent the 25‐ and 75‐percentile with median values as central band; whiskers span the 1.5‐fold interquartile range. Each data point depicts a patient‐derived sample as indicated in Table EV1.FPearson correlation of median intensity values across the groups of our lung cohort, indicating the overall level of similarity between COVID‐19 to the other lung diseases.

To separate nonsystemic from the systemic effects in the tissue proteome changes, we implemented the deconvolution of the systemic effects of COVID‐19 as described above, first focusing on the manifestation of COVID‐19 in the lungs. Notably, the selection of control samples included clinical factors such as pre‐existing chronic respiratory disease and smoking status into the study design (Dataset EV2 and Table EV4). In comparison to healthy lung tissue (nonpathologic controls, NPC), 384 and 533 proteins were up‐ and downregulated in COVID‐19, respectively (Fig 3A). Fibroblast growth factor receptor substrate 3 (FRS3) and the negative regulator of collagen production reticulocalbin‐3 (RCN3) were among the most upregulated (5.4‐ and 3.9‐fold, respectively). Interestingly, proteomics directly revealed details on the immune defense against viral infections, including drastic increase in antigen CD177, Integrin alpha‐M (ITGAM) as well as the complex‐forming E3 ubiquitin‐protein ligase DTX3L and PARP9. In contrast, proteins providing tissue structure such as the basal cell adhesion molecule (BCAM) and the tight junction organizing protein EPB41L5 were significantly downregulated. Further downregulated proteins of functional or structural significance in the lungs were the alveolar type I (AT1) cell lineage markers AGER/RAGE, CLIC5 as well as Caveolin‐1 (CAV1) and the pulmonary surfactant‐associated protein C (SFTPC) which is exclusively expressed in alveolar type II (AT2) cells. Reassuringly, proteins prominently discussed here such as RCN3 and NNMT showed comparable fold changes and directionality compared to previous work (Nie *et al*, 2021; Appendix Fig S2C and D). In contrast, we observed a somewhat greater increase of the acute‐inflammatory protein CRP, perhaps indicating greater levels of inflammation in our clinical cohort. Conversely, we confirmed concordant upregulation in the lung of the transcription activators STAT1‐3, cathepsins as well as the cytokine‐related protein NAMPT, thrombospondin‐1 (THBS1), and CHI3L1, which modulates fibroblast proliferation (Dataset EV4).

We next extended the protein‐level analysis with a biological pathway enrichment in comparison to NPC specimen. This highlighted specific processes that divided in either enriched or downregulated processes in COVID‐19 (Fig 3B). Some reflected protein assemblies directly involved in defense against the viral infection, such as phagocytic degradation and interferon signaling, as well as viral mRNA translation and protein cargo concentration in the ER induced by protein translation. Notably, we also observed increased levels of proteins related to ‘nicotinate metabolism’. Next to proteins involved in the synthesis and processing of nicotinamide adenine dinucleotide (NAD), the nicotinamide N‐methyltransferase (NNMT) was among the most significantly upregulated proteins in COVID‐19 compared to control samples of the lungs. In contrast, processes associated with the maintenance of tissue structure such as elastic fiber formation and the organization of the interaction of cells with the extracellular matrix appeared substantially less abundant in COVID‐19 tissue specimen, supporting the protein‐level results described just above. Interestingly, also the activity of RHO GTPases showed decreased effects, pointing out alterations in the cytoskeletal organization and actin/tubulin dynamics in the cell. A detailed protein composition of the respective pathway term is given in Dataset EV4.

### Classification of COVID‐19 among lung disease of related pathology

**Figure EV3:**
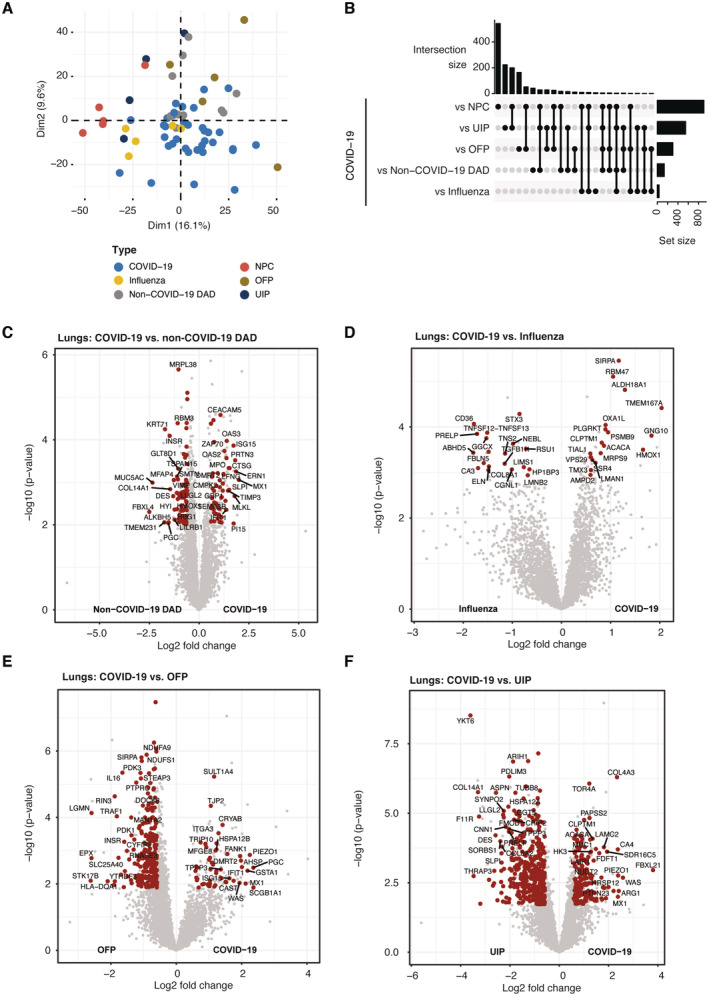
Stratification of COVID‐19 related pathologies in the lungs APrincipal component analysis (PCA) of lung samples derived from COVID‐19 and lung pathologies sharing similar phenotype features as well as the nonpathological control group.BUpset plot depicting the intersection of differentially regulated proteins between COVID‐19 and lung pathologies sharing similar phenotype features as well as the nonpathological control group.C–FDifferential regulation of phospho‐sites derived from pairwise comparisons between COVID‐19 and the control groups of the lungs: non‐COVID‐19 DAD (A), Influenza (B), fibrosing organizing pneumonia (OFP, C) and usual interstitial pneumonia (UIP, D). Protein significance (*t*‐test, *q*‐val < 0.05, fold change > 1.5) is highlighted in red.

To contrast the pulmonary manifestations of COVID‐19 to other lung diseases of related pathology in addition to the NPCs, we next compared our COVID‐19 results to those from samples of patients with influenza, non‐COVID‐19 DAD, common interstitial pneumonia (UIP), and fibrosing organizing pneumonia (OFP). In a principal component analysis (PCA), NPC control samples and UIP separated distinctly from COVID‐19 specimen of the lungs, whereas influenza and non‐COVID‐19 DAD partially overlapped with COVID‐19 (Fig EV3A). Comparing each lung pathology to the proteome of COVID‐19 lungs, we found that the number and overlap of altered proteins was most significant in comparison to UIP followed by NPC control samples, indicating major differences of COVID‐19 to usual interstitial pneumonia (minimum fold‐change 1.5 at a *q*‐value of <0.05, Fig EV3B–F, Dataset EV4).

To stratify COVID‐19 from other lung disease, we next performed an ANOVA (significance cutoff ct < 0.001) followed by post‐Tukey testing (adj. *P*‐value < 0.05) to ascertain differential protein regulation in at least two lung pathologies compared to COVID‐19. This identified 97 significant proteins, grouped in seven different clusters (Fig 3C). Cluster 2 specifically differentiated COVID‐19, non‐COVID‐19 DAD and OFP from influenza and UIP. It showed increased abundances of the AP‐3 complex subunit AP3B1, somatic mutations of which confer higher risk for severe COVID‐19 (Luo *et al*, 2021) as well as the collagen‐associated protein PLOD3, which is associated with common pulmonary fibrosis (Shao *et al*, 2020; exemplary shown in Fig 3D and E). Likewise, levels of the ER‐associated protein Vigilin (HDLBP) were elevated, which has already been reported during a SARS‐CoV‐2 infection (Flynn *et al*, 2021) and procollagen galactosyltransferase 1 (COLGALT1), correlating with increased generation of collagen in lung tissue.

In the UIP group in cluster 3, levels of ATM kinase, SEC31A1 a component of COPII complexes and the mediator of COPI/COPII‐based transports TMED10, were elevated. Thus, cluster 3 is likely to display shared properties of virus‐induced and pneumonia‐related rearrangements via the COP vesicle system within the cell.

Cluster 6 mirrored cluster 2 as it differentiated the same patient groups, however by proteins that decreased in abundance compared to NPC controls. Among those was Elastin (ELN), a major component of the extracellular matrix, along with the respiratory tract defense Antileukoproteinase (SLPI) that has previously been associated with pseudomonas‐induced fibrosis due to the degradation of neutrophil elastase (Weldon *et al*, 2009; Camper *et al*, 2016; Mecham, 2018).

Cluster 7 reflected the gradual downregulation of proteins across lung diseases toward COVID‐19 compared to UIP and NPC controls. This included Fibrillin‐1 (FBN1), Fibulin‐5 (FBLN5) and Fibromodulin (FMOD), which suggested a decline of microfibrils in the extracellular matrix. The antiviral protein NLRX1 was another interesting member of this cluster that was significantly downregulated compared to the majority of patients in the other lung diseases.

Driven by the considerable differences between the lung diseases, we tested for the level of correlation between each group of our lung cohort using median intensity values (Fig 3F). The proteomic signature of COVID‐19 correlated most closely with non‐COVID‐19 DAD (Pearson correlation 0.97). Specimen of influenza and OFP also correlated highly with the COVID‐19 phenotype (0.94), whereas usual pneumonia (UIP) correlated less (0.89).

Thus, our proteomic characterization enabled the stratification of COVID‐19 with respect to other lung diseases, revealing overall similarity to non‐COVID‐19 DAD and distinct differences to influenza‐related lung damage. The molecular similarity between COVID‐19 and non‐COVID‐19 DAD reflected the main histopathologic consequence of SARS‐CoV‐2 infection in the lungs, known as dominant diffuse alveolar damage. The proteomic overlap between COVID‐19 and influenza, in contrast, indicated similarities in disease manifestation and mechanisms, but pointed out distinct proteomic features of each cohort group.

### Infection‐induced phosphorylation signaling in the lungs

**Figure EV4:**
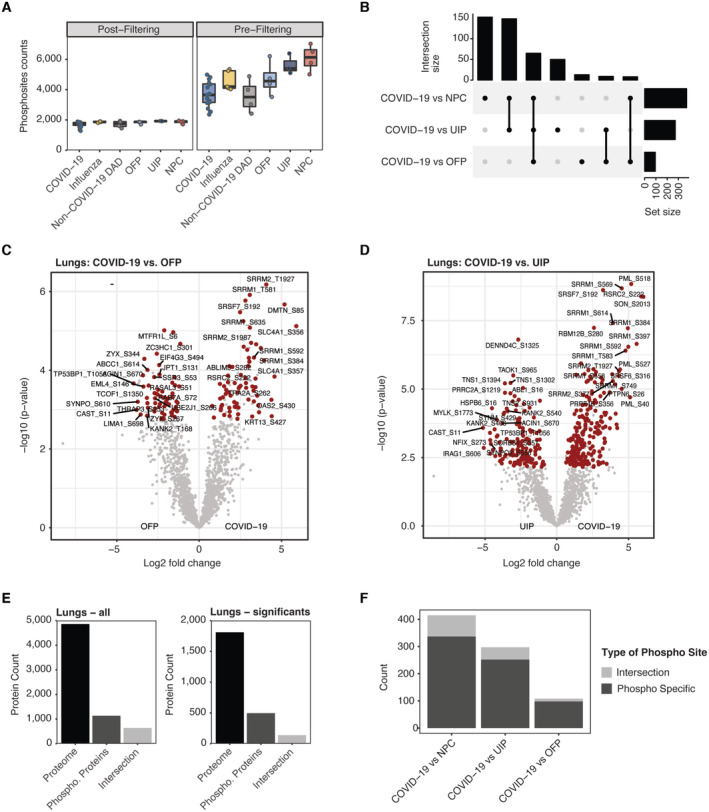
Quantitative assessment of phospho‐proteomic data ACounts of quantified phosphorylation sites in COVID‐19 and all control groups. Data are shown before (right) and after (left) stringent filtering as described (Materials and Methods). Boxplots represent the 25‐ and 75‐percentile with median values as central band; whiskers span the 1.5‐fold interquartile range. Each data point depicts a patient‐derived sample for COVID‐19 (*n* = 16), Influenza (*n* = 5), Non‐COVID‐19 DAD (*n* = 4), OFP (*n* = 4), UIP (*n* = 3) and NPC (*n* = 4).BUpset plot depicting the intersection of differentially expressed proteins across between COVID‐19 and NPC, OFP or UIP, respectively. Statistical testing in comparison to Influenza and non‐COVID‐19 DAD did not result in significant hits.C, DDifferential phospho‐site regulation between COVID‐19 and OFP (C) or UIP (D), respectively. Protein significance (*t*‐test, *q*‐val < 0.05, fold change > 1.5) is highlighted in red.EIntersection between phosphorylated proteins and full proteome in this dataset. Bars show count of identified proteins (black), phosphorylated sites summarized in protein groups (dark gray) and the overlap of the former ones (Intersection, light gray). The analysis for all proteins (left) and significantly differentially regulation (left) determined between all controls and COVID‐19 in the lungs of this study show a low degree of corresponding proteomic and phospho‐proteomic information.FCount of phospho‐sites separated into unique differential alteration on the level of protein phosphorylation (Phospho Specific) compared to the portion of sites which correspond to significant differential expression on the protein level (Intersection).

To investigate how SARS‐CoV‐2 impacts intracellular signaling cascades in the lungs, we performed quantitative phospho‐proteomics, using a recently described direct DIA workflow employing a FAIMS library (Stukalov *et al*, 2021). Here we analyzed FFPE tissue samples from 16 COVID‐19 and 20 control samples including representative specimen from all previously described non‐COVID‐19 pathologies. Among those, 28 samples directly matched samples of the proteome measurements, whereas 17 samples reflected the proteome of multiple specimens within one group. Overall, we identified 11,531 phosphorylation sites on 2,681 proteins which were filtered stringently to achieve up to 2,000 sites of high confidence in each group and equal identification levels among all groups of our cohort (Fig EV4A).

**Figure 4:**
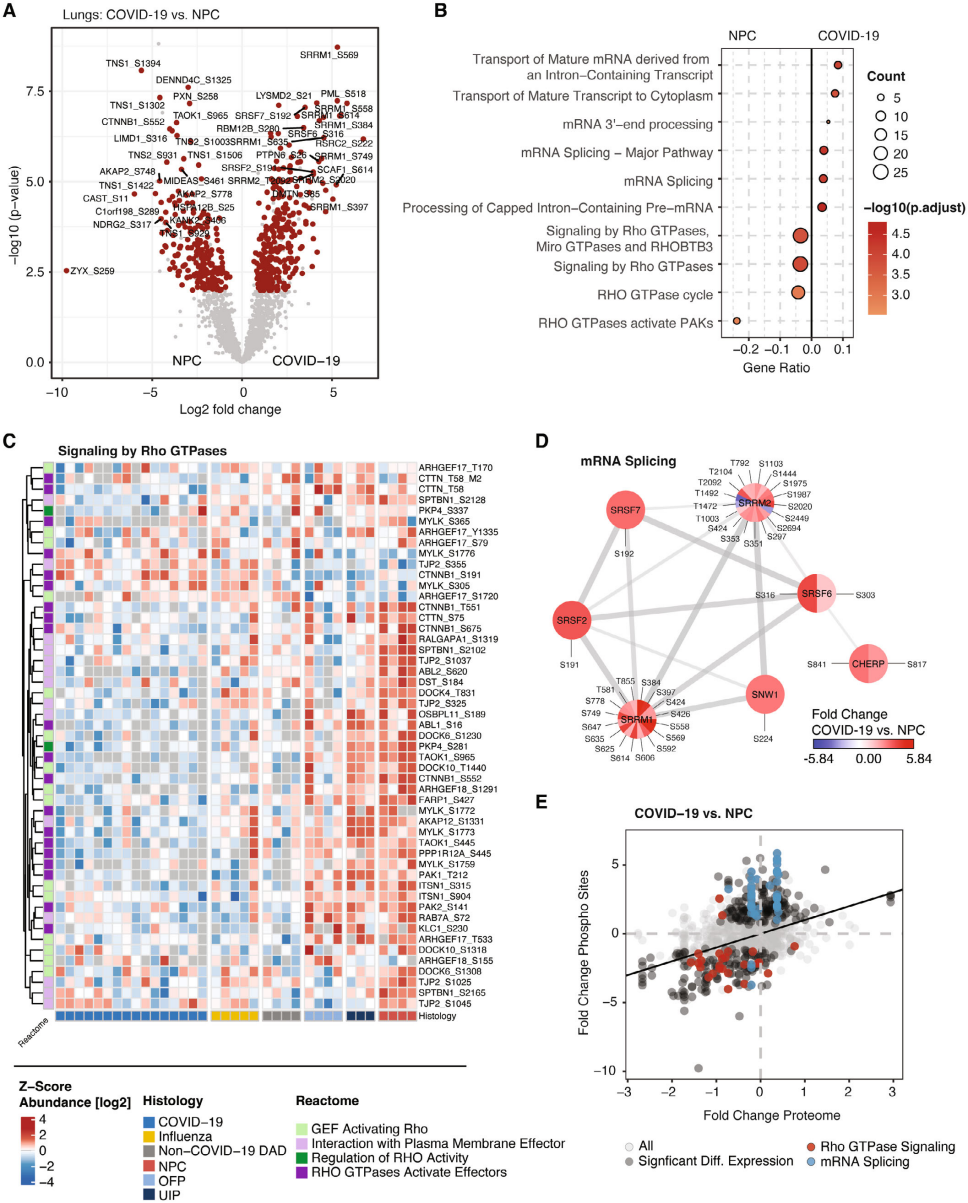
Infection‐induced phosphorylation signaling in the lungs Differential phospho‐site regulation between COVID‐19 and nonpathological controls (NPC). Significant proteins (*t*‐test, *q*‐val < 0.05, fold change > 1.5) are highlighted in red.Biological pathway enrichment (ORA) for the differentially regulated proteins in COVID‐19 and NPCs in (A) using the pathway Reactome database. The count of proteins associated with each term is indicated by point size.Heatmap of proteins involved in signaling by Rho GTPases in (B). Annotations show the groups of lung diseases (bottom) and biological roles in the Rho Pathway (left). COVID‐19 and control groups partially or entirely match the groups shown in the full proteome of the lungs (Fig 3).Network representation of proteins with differentially regulated phosphorylation sites in the context of mRNA Splicing indicating their biological interaction. The color coding of each site visualizes the fold change between COVID‐19 and NPC.Comparison of fold changes between COVID‐19 and NPC on the phosphorylation and whole proteome level. Proteins associated with Rho GTPase signaling (red) and mRNA splicing (blue) are highlighted; significant differential expression (*t*‐test, *q*‐val < 0.05, fold change > 1.5) is indicated in gray.

Compared to COVID‐19, the phospho‐proteomes of NPC, UIP and OFP showed significant changes (*t*‐test, *q*‐val < 0.05, fold change > 1.5, Figs 4A and EV4B–D, Dataset EV5), which had also shown the largest differences at the proteome level. Of note, these parallel changes in the proteome may modulate the outcome of the phospho‐proteome. However, the degree of corresponding proteomic and phospho‐proteomic information specifically derived from FFPE tissue can vary, thereby raising the necessity for a case‐to‐case consideration of proteomic abundance changes (Fig EV4E and F).

Reactome biological pathway enrichment analysis of significantly changed phosphorylation sites between COVID‐19 and NPC revealed two main processes (Fig 4B). The first was a substantial downregulation of Rho GTPase signaling in the proteome of COVID‐19 (Fig 4C). This involved Guanine nucleotide exchange factors (GEFs) for the regulation of Rho GTPase activity such as the dedicator of cytokinesis proteins DOCK4, DOCK6, and DOCK10, suggesting a central role of the DOCK protein family which was already shown for DOCK2 in a genome‐wide association study (GWAS) in patients with severe cases of COVID‐19 (Namkoong *et al*, 2022). Additionally, downstream phosphorylation events of Rho GTPase signaling were decreased, on average 4.3‐fold. These included central kinases such as PAK1/2, the Wnt signaling pathway component Catenin beta‐1 (CTNNB1) as well as the plasma membrane effectors such as the small GTPase RAB7A, the latter regulating the expression of ACE2 on the cell surface in dependence on its phosphorylation status (Daniloski *et al*, 2021).

The second major process affected by virus infection was related to mRNA processing and splicing. Here, we noted the hyperphosphorylation of the serine/arginine repetitive proteins SRRM1 and SRRM2 playing a major role in pre‐ and postsplicing (Fig 4D). SNW1, a component of the spliceosome, and the S/N‐rich splicing factors SRSF2, SRSF7 and SRSF6 likewise showed increased phosphorylation. These results suggest that SARS‐CoV‐2 modulates host splicing machinery by acting on phosphorylation, presumably to facilitate virus replication.

Notably, the increase in phosphorylation of the mRNA processing, splicing machinery, and Rho GTPase signaling was additive to the significant changes in protein levels (Fig 4E) and mRNA‐related processes were consistent with results from our recent multi‐omics study of SARS‐CoV‐2 studying altered phosphorylation in a lung‐derived human cell line (Stukalov *et al*, 2021).

### Organ‐specific effects in COVID‐19

**Figure 5:**
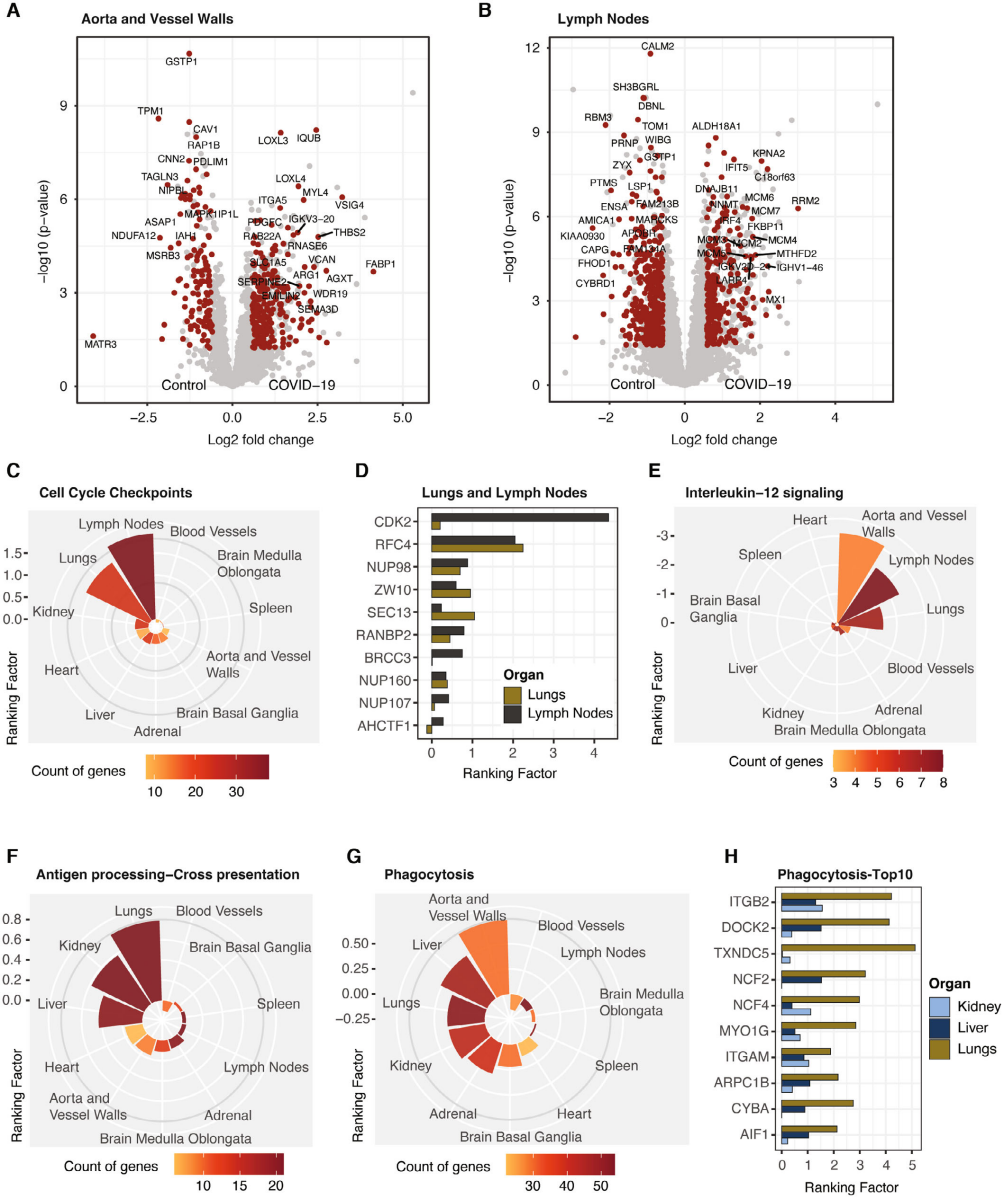
Organ‐specific effects in COVID‐19 A, BDifferential protein expression between COVID‐19 control specimens in the aorta/vessel walls (A) and lymph nodes (B). Protein significance (*t*‐test, *q*‐val < 0.05, fold change > 1.5) is highlighted in red.CMean ranking factor (*q*‐value (−log10) × fold change (log2)) of all proteins associated with the Reactome term “Cell Cycle Checkpoints” across all organs of this study. The number of genes identified for each organ is color coded.DBar plot representing the ranking factor values for proteins from (C) identified in common for lungs and lymph nodes. For clarity proteasomal subunits and MCM proteins (shown in Appendix Fig S3A) are excluded.E–GMean ranking factor values depicted as in (C) for proteins associated with Interleukin‐12 signaling (Reactome database, E), antigen processing‐cross presentation (Reactome database, F) and phagocytosis (GO biological processes, G).HBar plot representing the ranking factor values for the top 10 significant proteins from (G) by ranking factor identified in common for lungs, liver and kidney.

Having analyzed the distinctive proteome alterations in the lungs, we next evaluated the organ‐wide response upon SARS‐CoV‐2 infection beyond its entry portal. We first focused on the lymph‐blood vessel system as it was the second most affected tissue (Fig 2G, Dataset EV6). Among the proteins of the aorta/vessel walls, the downregulation of Matrin‐3 (MATR3) and Ras‐related protein Rap‐1b (RAP1B) related to a decreased capacity to maintain endothelial cell polarity and vascular integrity (Lakshmikanthan *et al*, 2014; Quintero‐Rivera *et al*, 2015; Fig 5A). Conversely, the regulator of inflammatory response LOXL3, the phagocytic receptor and regulator VSIG4 and platelet‐derived growth factor C (PDGFC) were significantly upregulated in the proteome of COVID‐19. While these proteins indicated an active inflammatory response, wound healing and fibrotic disease, those of the interferon system, complement and coagulation showed no specificity to the blood vessel system (Fig 2A).

Between the lymph nodes of COVID‐19 and controls, 252 proteins were upregulated and 366 downregulated, after separating the organ‐specific from the systemic effects as described above (Fig 5B). The DNA replication licensing factor MCM2‐7 as well as the Ribonucleoside‐diphosphate reductase subunit M2 (RRM2), which has an essential role in DNA synthesis, were consistently upregulated by a mean of 3.5‐fold. In addition, we noted the upregulation of the nuclear importer protein KPNA2 that is targeted by the SARS‐CoV‐2 protein ORF6 resulting in nuclear exclusion of STAT1, thereby preventing antiviral signaling (Miyamoto *et al*, 2022). In contrast, proteins such as the RNA‐binding protein 3 (RBM3) that suppresses the activation of innate lymphoid cells in the lungs showed major downregulation (Badrani *et al*, 2022).

Biological pathway enrichment revealed processes such as ‘cell cycle checkpoints’ as upregulated in the lymph nodes which to less extend also occurred in the lungs (Fig 5C, Dataset EV6). In both organs ‐ apart from the MCM proteins (Appendix Fig S3A) – this was caused by proteins such as the Replication factor C subunit 4 (RFC4) and the nuclear pore complex protein NUP98, the latter being a direct target of viral ORF6 to prevent interferon signaling as shown for KPNA2 above (Miorin *et al*, 2020; Fig 5D). Cyclin‐dependent kinase 2 (CDK2) was also upregulated, but this was specific to the lymph nodes rather than in the lungs. RecQ‐like DNA helicase BLM, the cell cycle checkpoint control protein RAD9A, the origin recognition complex subunits ORC 3/4 and the Nucleoporin Nup37 among others were exclusively enriched in the lymph nodes (Appendix Fig S3B). Interestingly, interleukin‐12 signaling was strongly decreased in lymph nodes (represented by eight effectors) as well as to less extend in the lungs and aorta/vessel walls (Fig 5E, Appendix Fig S3C).

We next investigated the effect of COVID‐19 on the inner organs of the abdomen. Biological pathway enrichment revealed an increased abundance of proteins associated with the Reactome term “antigen processing‐cross presentation” in the kidney and liver apart from the lungs (Fig 5F). This was due to proteins such as the antigen peptide transporters 1 and 2 (TAP1/2) as well as the associated Tapasin (TAPBP) – core components of MHC class I antigen presentation. Of note, SEC61A1, a component of the translocon for the transport of polypeptides into the endoplasmic reticulum, was much less increased in the investigated organs compared to the lungs (Appendix Fig S3D). Indicating an active response to the antigen presentation, “Phagocytosis” was among the major upregulated processes in lungs, liver, and kidney (Fig 5G). Proteins in this term included the integrins ITGAM and ITGB2, indicating enhanced adhesion of immune cells, as well as Cytochrome b‐245 light chain (CYBA) and the neutrophil cytosol factor 4 (NCF4) promoting the phagocytic oxidative burst (Fig 5H). Proteins involved in liver metabolism such as Glutathione S‐transferase A1 (GSTA1) were concordantly downregulated in our and previous data (Nie *et al*, 2021; Appendix Fig S2C and D). Pathways uniquely enriched in the liver further suggested a loss‐of‐function effect through decrease in cholesterol biosynthesis, cytochrome P450 or glucuronidation (Appendix Fig S3E). Interestingly, we noted a reduced expression of valacyclovir hydrolase (BPHL) in the kidney of COVID‐19 patients, which is responsible for activation of anti‐viral nucleoside analogs such as acyclovir (Appendix Fig S3F). Of note, there was no significant association of proteomic changes across organs with the postmortem interval of patients (Appendix Fig S4A–C).

### Secondary inflammatory effects of COVID‐19 in the brain

**Figure 6:**
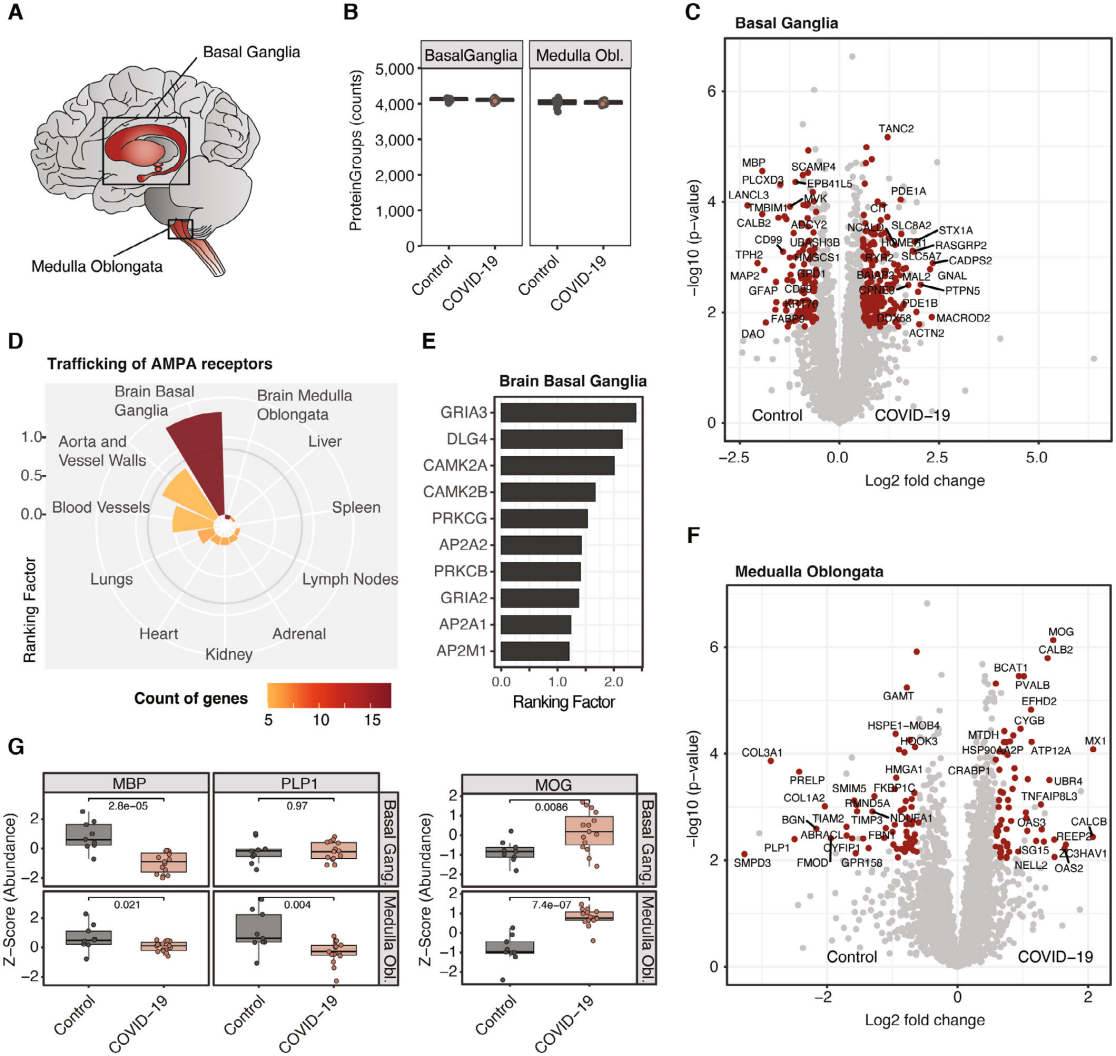
Secondary inflammatory effects of COVID‐19 in the brain Brain regions selected for proteomic analysis.Number of identified protein groups in the basal ganglia and medulla oblongata for COVID‐19 and control specimen. Each data point depicts a patient‐derived sample as indicated in Table EV1.Differential protein expression between COVID‐19 control specimen in the basal ganglia. Protein significance (*t*‐test, *q*‐val < 0.05, fold change > 1.5) is highlighted in red.Mean ranking factor (*q*‐value (−log10) × fold change (log2)) of all proteins associated with the Pathway term “Trafficking of AMPA receptors” (Reactome database) across all organs of this study. The number of genes identified for each organ in color codedBar plot representing the ranking factor values for proteins from (D) identified in the basal ganglia of the brain.Differentially expressed proteins between COVID‐19 and controls of the medulla oblongata as shown in (C).
*Z*‐Score protein abundance of myelin‐associated proteins which were derived from (C) and (F) for COVID‐19 and controls in the brain. An assessment of statistical significance (unpaired *t*‐test) is annotated for each comparison. Boxplots represent the 25‐ and 75‐percentile with median values as central band; whiskers span the 1.5‐fold interquartile range. Each data point depicts a patient‐derived sample as indicated in Table EV1.

As some symptoms of COVID‐19 infection indicate neurological deterioration, we aimed to investigate the proteome of the brain in the postmortem stage upon SARS‐CoV‐2 infection. To provide two contrasting examples of the brain by location and functionality, we analyzed specimen derived from the basal ganglia and the medulla oblongata in the brainstem (Fig 6A). In both regions, we quantified more than 4,000 protein groups that showed consistent identification numbers in both areas of COVID‐19 and control specimens that were largely balanced for age, sex and other clinical parameters (Fig 6B, Table EV5).

**Figure EV5:**
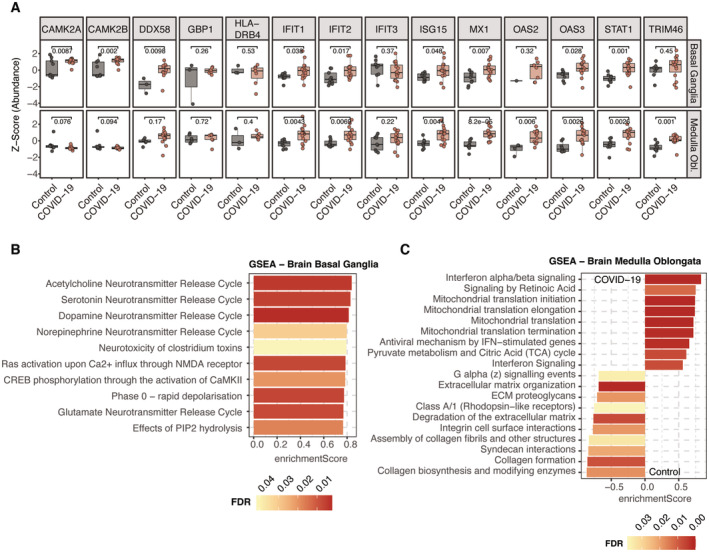
Biological processes associated with COVID‐19 of the brain A
*Z*‐Score protein abundance of proteins associated with “Interferon Signaling” (Reactome database) that showed overlapping occurrence in both regions of the brain. An assessment of statistical significance (unpaired *t*‐test) between COVID‐19 and control samples is annotated for each comparison. Boxplots represent the 25‐ and 75‐percentile with median values as central band; whiskers span the 1.5‐fold interquartile range. Each data point depicts a patient‐derived sample as indicated in Table EV1.B, CGSEA enrichment depicted by the top 10 most significant terms assigned by enrichment score. A positive enrichment score indicates positive protein abundance in COVID‐19, whereas a negative value indicates an enrichment in the control samples. The color coding of all bars depicts the FDR value of the respective enrichment term.

Although we did not detect viral peptides – concordant with PCR‐based analysis of the same specimens (Hirschbühl *et al*, 2021) – there was strong evidence of inflammation. This was specifically evident by proteins of the interferon signaling cascade such as the signal transducer and activator of transcription STAT1, the interferon‐induced enzyme OAS3 as well as the ubiquitin‐like protein ISG15 that showed significantly increased protein levels in COVID‐19 (Fig EV5A). While the upregulation of interferon‐related effectors was shared with the majority of organs, there were also specific changes in the brain.

For the basal ganglia, these biological processes were related to an increased activity of neurotransmitter release cycles (Fig EV5B). Specifically, this included substantial upregulation of TANC2, an mTOR regulator present in neurons (Kim *et al*, 2021b), Syntaxin‐1A (STX1A) that mediates the release of neurotransmitters from the synapse and the postsynaptic protein HOMER1 that interacts with RYR2 which was also upregulated (Fig 6C). GNAL, a signal transducer in the olfactory tract and the basal ganglia, was upregulated. In contrast, cell surface proteins that promote and regulate the adhesion to T‐cells (CD99 and the ubiquitin‐associated and SH3 domain‐containing protein B (UBASH3B)) showed lower abundances. While having a minor role in the medulla oblongata, “Trafficking of AMPA receptors” was specifically upregulated in basal ganglia, based on AMPA‐selective glutamate receptor 3 (GRIA3) as well as both subunits of the Calcium/calmodulin‐dependent protein kinase (CAMK2A/B) that regulate the signaling and trafficking of AMPA receptors by phosphorylation (Kristensen *et al*, 2011; Fig 6D and E). This suggests upregulation of AMPA receptors by inflammation (Guan *et al*, 2003).

GSEA enrichment in the medulla oblongata indicated increased levels of mitochondrial protein translation. Conversely, Rhodopsin‐like receptors and collagen biosynthesis showed negative regulation in COVID‐19, the latter indicating a loss of the stabilizing matrix in the brain (Fig EV5C). Notably, also the Zinc finger CCCH‐type antiviral protein 1 (ZC3HAV1) was highly expressed in COVID‐19 (Fig 6F). Reflecting the downregulation of Rho GTPase signaling in the phosphoproteomic analysis of the lungs, we found reduced levels of Biglycan (BGN) and the Rho guanine nucleotide exchange factor TIAM2.

Interestingly, the myelin proteolipid protein (PLP1) and the neutral sphingomyelinase 2 (SMPD3) were among the most down‐regulated proteins in COVID‐19 of the medulla oblongata (5.6‐fold and 9.6‐fold). This finding was complemented by the substantial downregulation of the myelin basic protein (MBP) in both regions of the brain. In contrast, myelin‐oligodendrocyte glycoprotein (MOG) was among the most significantly upregulated proteins of COVID‐19 in the medulla oblongata, which correlates with higher levels of MOG antibodies seen in circulation in case reports of COVID‐19 (Durovic *et al*, 2021; Johnsson *et al*, 2022; Fig 6G). As myelin protein plasmolipin (PLLP) was also downregulated in the lungs of COVID‐19 (Fig 3A), this may be part of secondary inflammatory damage of myelin components in the CNS and other organs.

## Discussion

COVID‐19 is primarily a lung disease but is also associated with profound systemic inflammation in reaction to an infection with SARS‐CoV‐2. To date, numerous studies have investigated the physiological manifestation of COVID‐19 and the mechanisms of SARS‐CoV‐2 infection. However, its effects on the different tissues in the host organism are not yet entirely understood especially at the protein level, the primary mediators of biological processes. Taking advantage of autopsies from a pioneering cohort of the early pandemic phase in 2020 at the University Medical Center Augsburg (Schaller *et al*, 2020), we set out to apply state of the art MS‐based proteomics to investigate systemic and organ‐specific effects of viral infection on the host. We obtained samples from 10 different tissues of 19 postmortem donors and controls, resulting in more than 300 different tissue samples. To enable timely analysis of these FFPE samples, we developed a protocol that overcomes previous limitations of throughput and sample processing by reducing workflow complexity. Using our fast, parallelized and nontoxic workflow, we identified more than 7,000 proteins in a robust and quantitative manner as a basis to understand and classify the host proteome of COVID‐19. The identification of SARS‐CoV‐2 peptides in the lungs of severe COVID‐19 patients – something not reported for unbiased proteomics in the literature before – validated the depth and specificity of our technology as well the stability of proteins in a pathology‐relevant matrix. No peptides were identified beyond the lungs, consistent with the limited viral spread detected by RT‐qPCR in the same subjects (Hirschbühl *et al*, 2021).

Compared to other proteomics studies, our results confirm the global effects of inflammation seen in both plasma studies (Messner *et al*, 2020; Geyer *et al*, 2021) and a previous report on patient tissue (Nie *et al*, 2021). Although most substantial in the lungs, we observed organ‐wide occurrence of systemic inflammation in COVID‐19 represented by processes such as the complement cascade and interferon signaling and by central markers of inflammation such as the acute phase proteins CRP and LBP1. However, given the overlap inflammatory effects between plasma and all tissues in previous and our work, we suspected that the circulation‐derived proteome masked organ‐specific effects. This could be caused by blood contamination of tissues on the one hand, or the inflammatory response mediated by the circulation in the tissues. Beyond these direct and uniform effects, each tissue should also have its own specific response, mediated by tissue‐resident immune action (Farber, 2021). Hence, we concluded that the truly organ‐specific damage upon a SARS‐CoV‐2 infection had to be separated from the blood proteome associated one to better understand tissue‐specific effects in COVID‐19. To do this, we made use of our previous work defining panels of different classes of proteins in body fluids (Geyer *et al*, 2016; Karayel *et al*, 2022). This analysis strategy indeed efficiently unmasked the tissue‐specific effects of COVID‐19.

The resulting data depicted drastic lung‐specific changes beyond inflammation. It directly associated the characteristic fibroblastic proliferation with increased levels of the fibroblast growth factor receptor substrate FRS3 and the negative regulator of collagen production RCN3, thereby expanding the previous observations on fibroblast modulation in the lungs (Martinez‐Martinez *et al*, 2017; Nie *et al*, 2021). Conversely, decreased levels of lineage markers for alveolar type I and II cells reflected the destruction of the native cellular composition in the lungs at the protein level, also confirming previous work on the single cell RNA level (Melms *et al*, 2021). These markers also included AGER/RAGE, for which decreased abundances in serum of patients with severe disease progression was reported before (Yalcin Kehribar *et al*, 2021). This indicates a connection between stage of cellular loss in the lungs and this potential liquid biomarker. Our data further reveal that the loss of alveolar cells was accompanied by changes in the level of proteins indicating the destruction of the cellular environment and elastic fiber formation.

In comparison to other lung diseases of related pathology, COVID‐19 showed highest similarity to non‐COVID‐19 DAD, thereby reconfirming diffuse alveolar damage as the central histopathologic feature of COVID‐19 on the molecular level (Schaller *et al*, 2020). COVID‐19 together with non‐COVID‐19 DAD and OFP differed from influenza and UIP by profound collagenous fibrosis and germline variance observed for severe disease progression, such as for AP3B1 (Luo *et al*, 2021). The COVID‐19‐containing group also downregulated proteins involved in the generation of elastin polymers and microfibrillar structures suggesting a decline of the extracellular matrix. Furthermore, it showed decreased levels of the antiviral protein NLRX1 which is targeted by SARS‐CoV‐2 protein interaction via ORF9c (Gordon *et al*, 2020). Except influenza, all pathologies featured an increase in cellular transport and COPI/COPII vesicles.

Analysis of peptide phosphorylation patterns in SARS‐CoV‐2‐infected cells in culture revealed major rewiring of signaling pathways by the virus (Bouhaddou *et al*, 2020; Stukalov *et al*, 2021). Our workflow also allowed the characterization of altered signal transduction in an unbiased manner in primary patient tissue, recapitulating some of the major themes of our previous *in vitro* studies and their preservation in the postmortem state. This includes distinct downregulation of Rho GTPase signaling indicating central alterations in the organization of the cytoskeleton on the level of regulating kinases, such as PAK1/2, as well as downstream pathways. Furthermore, the upregulation of phosphorylation events in the context of RNA splicing support *in vitro* studies describing functional splicing as essential requirement for the life cycle of SARS‐CoV‐2 (Bojkova *et al*, 2020). In contrast, the role of SR proteins as regulators of cellular splicing during infection has only been reported in the context of HIV‐1 viral replication and release so far (Wojcechowskyj *et al*, 2013).

Beyond the lungs, our data reflected the substantial histopathologic changes of mediastinal lymph nodes that we reported earlier (Hirschbühl *et al*, 2021). This study extends this finding to the molecular level, showing increased levels of SARS‐CoV and SARS‐CoV‐2 specific antiviral signaling by proteins such as the nuclear importer KPNA2 and NUP98 in the lymph nodes (Frieman *et al*, 2007; Miorin *et al*, 2020; Miyamoto *et al*, 2022). Shared upregulation of the DNA replication machinery between lymph nodes and lungs, such as MCM proteins, suggests similarities in the induction of organ responses upon viral infection. In contrast to central mediators of inflammation such as interleukin‐6 (Rubin *et al*, 2021), interleukin‐12 signaling was specifically downregulated in the lymph nodes in severe COVID‐19 patients (Fig 5E) supporting reports of higher interleukin‐12 abundance in serum of mild compared to severe disease (Tjan *et al*, 2021). Beyond the lymph nodes, endothelialitis has been reported early in the pandemic (Varga *et al*, 2020). Our data mirror this finding by the upregulation of inflammatory and phagocytic mediators as well as corresponding indicators of vascular damage. Processes associated with coagulation and interferon‐regulated inflammation, however, showed no specificity to the blood vessel system.

Inner organs such as the liver, kidney, and spleen rarely show infection with SARS‐CoV‐2 in histopathology or RNA sequencing. It was therefore of special interest that our proteomics results indicate active involvement of kidney and liver in the immune response of the host, mainly shown by antigen presentation and the corresponding response by phagocytosis. Decreased functionality of the liver and its metabolic functions has been reported in severe COVID‐19 (Mao *et al*, 2020; Nie *et al*, 2021) and this is supported by our protein level data.

Direct invasion of SARS‐CoV‐2 into the central nervous system and morphological changes during disease progression in the brain are controversially discussed (Matschke *et al*, 2020; Hirschbühl *et al*, 2021; Schwabenland *et al*, 2021; Douaud *et al*, 2022). Here, we confirm highly prevalent interferon signaling in the basal ganglia and medulla oblongata on the proteomic level. In the basal ganglia, the inflammatory signature was accompanied by substantial proteomic alterations in synapses such as increase in proteins involved in the release of neurotransmitters and those associated with trafficking of AMPA receptors. Altered processes in the medulla oblongata included a loss in the stabilizing extracellular matrix and decrease of Rho signaling, the latter shared with the lungs. Most strikingly, proteins associated with the structure and maintenance of myelin were substantially reduced (Fig 6G). Thus our proteomic results directly document the consequences of secondary inflammation in the brain, correlating with the senso‐motoric symptoms that have been extensively described COVID‐19 (Venkataramani & Winkler, 2022) and the potential for long‐COVID‐19. Degradation of myelin sheaths of the neuronal system is accompanied by inflammatory processes closely related to aging in the brain (Safaiyan *et al*, 2016; Kaya *et al*, 2022), which in turn is increasingly associated with COVID‐19 (Mavrikaki *et al*, 2022).

In summary, the quantitative proteomics approach of this study showed the specific involvement of multiple organs in the host response to COVID‐19 to different degree in systemic inflammation and beyond. Our investigation covers the early phases of the pandemic, whereas the virus has mutated considerably since, impacting transmission rates and pathogenicity. For instance, the Delta variant (B.1.617.2) was highly transmissible and might have been associated with an increased risk for hospitalization. Likewise, pathogenicity differed between virus variants and host mortality was reduced by factors such as the improvement of the host immune response via vaccination or infection over the course of the pandemic (Carabelli *et al*, 2023). These developments also suggest altered effects on the host tissue proteome and could be investigated by stratifying SARS‐CoV‐2 variants, disease progression, immunization status and patient demographic characteristics.

Despite the careful design of this study, certain aspects may limit its medical significance. These include preanalytical variations during sample retrieval and preservation. Despite of a control cohort of largely balanced clinical characteristics, heterogeneity of clinical treatment strategies and minor differences in comorbidities could also have an effect on the clinical interpretation of data. Furthermore, the number of samples organs may limit conclusions on the whole‐organ level. In summary, our proteomics workflow allowed the in‐depth profiling of 352 tissues of a postmortem COVID‐19 and control cohort. Besides recapitulating findings already reported in the literature, we here extend the number of previously investigated organs (Nie *et al*, 2021) and provide an in‐depth and systematic insight into the manifestation of COVID‐19 beyond inflammation. Moreover, we characterize COVID‐19 in the lungs as compared to other lung diseases of similar pathology. While supporting or questioning others, our resource gives rise to new hypotheses that can be validated or used in translational context by the community.

## Materials and Methods

### Study design and ethical approval

From April 4 to May 13, 2020, 19 autopsies (15 full and 4 through limited infrasternal access) were performed. Eighteen patients died at the University Medical Center Augsburg (UKA), representing 86% of all Covid‐19 deceased at the UKA during the first wave of the pandemic. All cases were tested positive for SARS‐CoV‐2 by nasopharyngeal swabs during the clinical course and postmortem. SARS‐CoV‐2 RNA was quantified by RT‐qPCR as previously described (Hirschbühl *et al*, 2021). Before autopsy, informed consent from next of kin was obtained. Clinical data (including medical history, comorbidities, medication, and treatment) were obtained from electronic medical records. This study was approved by the internal review board of the UKA (BKF No. 2020‐18) and the ethics committee of the University of Munich (Project number 20‐426, COVID‐19 registry of the UKA). Ethical approval for the use of tissue samples as controls for the characterization of COVID‐19‐associated proteomic alterations is based on the study protocol of the COVID‐19 registry of the University Hospital Augsburg (COKA). The study protocol of this registry specifically includes the possibility to include SARS‐CoV‐2 negative patients as a control group and was positively approved by the ethics committee of the Ludwig‐Maximilians‐University Munich under the project no. 20‐426. All experiments conformed to the principles set out in the WMA Declaration of Helsinki and the Department of Health and Human Services Belmont Report.

### Processing of tissue samples

Tissue samples from organs (lungs, heart, vessels, spleen, kidneys, and brain) were immediately fixed in buffered 10% formalin solution after harvesting. After fixation (at least 12 days – 12 to 15 days), representative formalin‐fixed and paraffin‐embedded samples (FFPE) were generated. FFPE tissue was cut (10 μm) and mounted on common microtome slides (TOMO IHC Adhesive Glass Slide, TOMO 1190, Matsunami, Japan) or membrane slides (1.0 PEN, Prod. No. 415190‐9041‐000, Zeiss, Germany). Lung tissue was deparaffinized and stained by Hematoxylin and Eosin for pathology assessment. Regions representing the overall histology of each patient were annotated by a pathologist (T.S.) and patient material was collected using a scalpel in a defined area of 25 mm^2^ for each patient and organ. This procedure resulted in a total of 305 samples that were prepared for MS‐based proteomics. For phospho‐proteomic analyses, FFPE tissue was collected likewise from the entire slide to match the COVID‐19 cohort of the lungs. Aiming for a total peptide amount of 85 μg in each sample prior to phospho‐peptide enrichment, we directly matched 28 samples to corresponding samples of the full proteome, while 17 samples were combined within groups resulting in a total of 36 samples for phospho‐proteomic data acquisition.

### Sample preparation

FFPE tissue was collected into a 96‐well plate (AFA‐TUBE TPX Plate, Prod. No. 520291, Covaris, Brighton, U.K.) keeping each tissue type within one plate. For each of these plates, COVID‐19 and control samples of matching tissues were randomized and measured without interruption, avoiding batch effects. Paraffinized and H&E‐stained tissue was processed equally using Adaptive Focused Acoustics® (AFA®, Covaris) sonication and adapted Protein Aggregation Capture (PAC; Batth *et al*, 2019), herein in combination termed APAC. In brief, we accomplished tissue lysis and, if present, paraffin dissociation, by sample heating and sonification. Proteins were subsequently extracted from the solution by precipitation on magnetic particles and purified by extensive washing at 50°C in isopropanol. Tryptic digestion yielded deparaffinized peptides that were finally subjected to MS‐measurement.

In detail, 40 μl of tissue lysis buffer (truXTRAC Proteins – Tissue Lysis Buffer, Prod. No. 520284, Covaris) were added to each sample aliquot, followed by a pre‐incubation for 10 min at 90°C in a PCR cycler. Subsequently, samples were sonified in the 96‐well layout for a total duration of 300 s per column (LE220‐plus Covaris Focused‐ultrasonicator, Covaris) and defined parameters (peak Power: 450.0, duty factor: 50%, cycles: 200, average power: 225). Tissue lysis was resumed during an incubation for 80 min at 90°C in the PCR cycler and concluded by repeated sonification. Enclosing, proteins were reduced and alkylated by 5 mM DTT and 20 mM CAA final concentration for 20 min while shaking at 1,400 rpm (ThermoMixer C, Hamburg, Germany) at room temperature, respectively. In preparation for Protein Aggregation Capture, magnetic carboxylate modified particles (Sera‐Mag™, Prod. No. 24152105050350, GE Healthcare/Merck KGaA, Darmstadt, Germany) were washed trice with 1 ml MS‐grade H2O a constant amount of 300 μg beads was added to each sample well. Protein precipitation was induced by the addition of acetonitrile to a final volume of 70%. To ensure complete precipitation, we incubated the suspension for 10 min at room temperature while shaking at 1,200 rpm and allowed beads to settle down for a further 10 min without agitation. Subsequent to the removal of supernatant on a magnetic rack (e.g. DynaMag™‐96 Side Skirted Magnet, Prod. No. 12027, Invitrogen, Thermo Fisher Scientific, Darmstadt, Germany), beads were washed trice in 100% isopropanol for 10 min at 50°C and shaking at 1,400 rpm to clear all paraffin. For the enzymatic digest, we estimated the protein yield from 25 mm^2^ starting material based on a previous evaluation for the processing of FFPE tissue (Coscia *et al*, 2020). Consequently, beads of each sample well were resuspended in 100 μl of 100 mM Tris, pH 8.5, supplemented with 0.5 μg of trypsin and LysC, respectively, and incubated overnight at 37°C and 1,300 rpm. On the next day, we enclosed a postdigest of 4 h at 37°C and 1,300 rpm with 0.5 μg of both enzymes. Hereafter, the supernatant was removed completely while placing the 96‐well plate on the magnetic rack and transferred twice to a 96‐well PCR plate (twin.tec^®^ PCR Plate LoBind^®^, semi‐skirted, 250 μl, Prod. No. 0030129504, Eppendorf, Hamburg, Germany) to remove residual magnetic particles. The enzymatic reaction was quenched using TFA at a final concentration of 1% (v/v) and resulting peptides were stored at −20°C until further processing. To generate deep proteomic profiles for specific tissues such as the lung and the lymph nodes, 30–50 μg of peptides were pooled for the respective organ as well as separately for either samples derived from patients with SARS‐CoV‐2 or their controls. Each sample pool, herein called ‘libraries’, was subsequently fractionated into 24 fractions by high‐pH reversed‐phase chromatography using the “Spider fractionator” as described previously (Kulak *et al*, 2017). In preparation for the LC–MS measurement using an Evosep LC system, peptides of the single‐shot samples and the deep proteomes were loaded on C18 EvoTips according to the manufacturer's instructions (Evosep, Odense, Denmark) and stored at 4°C until further processing.

The AssayMAP Bravo robot (Agilent) performed the enrichment for phosphopeptides (80 μg) by priming AssayMAP cartridges (packed with 5 μl Fe^3+^‐NTA) with 0.1% TFA in 99% ACN followed by equilibration in equilibration buffer (1% TFA/80% ACN) and loading of the same amount of peptides resuspended in equilibration buffer. Enriched phosphopeptides were eluted with 1% ammonium hydroxide, which was evaporated using a Speedvac for 20 min. Dried peptides were resuspended in 6 μl 2% acetonitrile (v/v)/ 0.1% trifluoroacetic acid (v/v) and 5 μl were analyzed by LC–MS/MS.

### Liquid chromatography and mass spectrometry

For the LC–MS/MS analyses of the full proteome, we coupled the Evosep One platform online to an Orbitrap Exploris 480 Mass Spectrometer (Thermo Fisher Scientific, Waltham, USA) using a nano‐electrospray ion source (Thermo Fisher Scientific). Peptides were loaded on a 15 cm HPLC column (inner diameter: 150 μm; generated in‐house with ReproSil‐Pur C18‐AQ 1.9 μm silica beads (Dr. Maisch GmbH, Ammerbuch, Germany; Müller‐Reif *et al*, 2021)) that was kept at 60°C by an oven containing a Peltier element (in‐house development). Each sample was separated on the analytical column employing the standardized gradient of 88 min (15 samples per day) from the Evosep^+^ method set. Data for single‐shot samples were acquired in a data‐independent (DIA) mode with full MS scans (scan range: 300–1,650 *m/z*; resolution: 120,000; maximum injection time: 50 ms; normalized AGC target: 300%) and 40 periodical MS/MS segments applying isolation windows based on peptide prediction in MaxQuant.live (resolution: 30,000; maximum injection time: 50 ms; normalized AGC target: 1000%). Peptide fragmentation was enabled using a normalized collision energy of 30%. Fractions of the pre‐fractionated library were processed in data‐dependent acquisition (DDA) mode (Top12 method) with full MS (scan range: 350–1,400 *m/z*; resolution: 60,000; maximum injection time: 25 ms; normalized AGC target: 300%) and MS/MS scans (resolution: 15,000, isolation window: 1.3 *m/z*, normalized collision energy: 30%, maximum injection time: 22 ms, normalized AGC target: 200%, dynamic exclusion: 30 s). All spectra were acquired in profile mode using positive polarity.

For phospho proteomic analyses, samples were loaded onto a 50‐cm reversed‐phase column (75 μm inner diameter, packed in house with ReproSil‐Pur C18‐AQ 1.9 μm resin (Dr Maisch)). The column temperature was maintained at 60°C using a homemade column oven. A binary buffer system, consisting of buffer A (0.1% FA) and buffer B (80% ACN plus 0.1% FA) was used for peptide separation, at a flow rate of 300 nl min^−1^. An EASY‐nLC 1200 system (Thermo Fisher Scientific) for nano‐flow liquid chromatography was directly coupled online with the mass spectrometer (Orbitrap Exploris 480, Thermo Fisher Scientific) via a nano‐electrospray source. The FAIMS device was placed between the nanoelectrospray source and the mass spectrometer for spectral library generation. Spray voltage was set to 2,650 V, RF level to 40 and heated capillary temperature to 275°C. Five microliters of phosphopeptide samples were loaded and eluted with a 70‐min gradient starting at 3% buffer B followed by a stepwise increase to 19% in 40 min, 41% in 20 min, 90% in 5 min and 95% in 5 min. The mass spectrometer was operated in DIA mode (in profile mode using positive polarity) with a full scan range of 300–1,400 *m/z* at 120,000 resolution at 200 *m/z* and a maximum fill time of 60 ms. One full scan was followed by 32 windows with a resolution of 30,000. Normalized AGC target and maximum fill time were set to 1,000% and 54 ms, respectively. Precursor ions were fragmented by HCD (NCE stepped 25–27.5–30%). For the library generation, pooled phospho‐enriched sample was measured with 11 different CV settings (−30, −40, −45, −50, −55, −60, −65, −70, −75, −80 or −90 V) using the same DIA method. The noted single CVs were applied to the FAIMS electrodes throughout the analysis.

### Data processing

Single‐shot data were processed organ‐wise in Spectronaut version 14.7.201007.47784 (Copernicus, Biognosys AG, Schlieren, Switzerland) employing a library‐free strategy (directDIA) and searching against the Uniprot human databases UP000005640_9606, UP000005640_9606_additional (integrated in the Spectronaut software; 21,039 and 70,579 entries, respectively) and the severe acute respiratory syndrome coronavirus 2 (downloaded on July 28 2020, 120 entries). Enzymatic cleavage was defined by Trypsin/P with a peptide length of 7–52 and a maximum number of two missed cleavages. Carbamidomethylation was set as a fixed modification while methionine oxidation and N‐terminal acetylation were indicated as variable modifications. A minimum number of 3 and a maximum number of 6 were selected as Best N Fragment per peptides. Significance filtering followed a precursor and protein *q*‐value cutoff of 1%. A global normalization of data based on median quantities was enabled, eliminating possible MS intensity drift over time. Other processing parameters were kept by standard settings of the Spectronaut version.

For phospho‐proteomic data, Spectronaut version 16.2.220903.53000 (Biognosys; Copernicus, Biognosys AG, Schlieren, Switzerland) employing the previous databases was used to generate DIA libraries and analyze DIA single‐runs. Notably, these FASTA files also included protein isoforms which may be relevant for the interpretation of resulting phosphorylation sites. The library consists of DIA single‐runs acquired with a static CV ranging from −30 to −90. For the generation of the hybrid library, the library runs were combined with cohort samples measured also as DIA single‐runs, and phosphorylation at Serine/Threonine/Tyrosine was added as a variable modification to default settings. The maximum number of fragment ions per peptide was increased from 3 to 25. The biological replicate files were analyzed against the hybrid library. Phosphorylation at Serine/Threonine/Tyrosine was added as a variable modification to default settings with a disabled PTM localization filter. An FDR of 1% determined the significance level.

### Bioinformatics data analysis

Bioinformatics data analysis was performed using the R statistical computing environment version 4.0.2. In preparation for the analysis, protein intensities were log2‐transformed. For the Principal Component Analysis (PCA) and ANOVA, protein identifications were filtered for a minimum of 80% valid values in each group and imputed sample‐wise based on a normal distribution (width of 0.3, downshift of 1.8; FactoMineR package). For the analysis of variance (ANOVA) testing, the R stats package was employed. *P*‐values were adjusted using the Benjamini–Hochberg method, followed by post hoc computation of the Tukey Honest Significant Differences (Tukey HSD) at a confidence level of 0.95. Differential expression was determined for proteins with a minimal identification of three valid values in total using Student's *t*‐tests (unpaired, variances assumed to be equal), *P*‐value correction (qvalue package) and filtering for significant differential expression (*q*‐value < 0.05, fold change ≥ 1.5). For the comparison of differential expression across organs, we calculated the ranking factor for each protein, defined as the product of fold‐change and statistical consistency (*q*‐value; Xiao *et al*, 2014).

Marker proteins for coagulation, platelets, and red blood cells (RBC) were extracted from Karayel *et al* (2022), and the 30 most abundant and nonredundant proteins from the plasma were added from Geyer *et al* (2016). Proteins of this systemic‐inflammatory response were complemented by immunoglobulins identified in our dataset (Dataset EV3). For the “filtering” of systemic‐inflammatory signatures, statistical tests were performed on the entire set of proteins and subsequently filtered based on the previously generated table.

Pearson correlations between groups were determined using the R stats package on median protein intensities per group only considering complete observations. Biological pathway enrichment of significantly differentially regulated proteins in each organ was performed for the entire dataset including the systemic‐inflammatory and the organ‐specific effects, respectively, using the WebGestalt gene set analysis toolkit for a GSEA (organism: homo sapiens, FDR threshold: 0.05, databases: ‘pathway_KEGG’, ‘pathway_Reactome’, ‘geneontology_Biological_ Process_noRedundant’). Boxplots, point‐size, bar, radar and scatter (Volcano) plots were generated using the ggplot2 package, upset plots and heatmaps were generated via the ComplexHeatmap package.

To manually validate the quality of identified SARS‐CoV‐2 viral peptides, the corresponding fragment intensities were predicted using AlphaPeptDeep (Zeng *et al*, 2022) at normalized collisional energy 30, and were plotted with AlphaViz (preprint: Voytik *et al*, 2022), comparing them against the detected intensities at the apex elution positions. The elution profiles of the fragments in DIA data were extracted and plotted using AlphaViz. The matching mass error was set to 20 ppm for MS2 spectra.

For the analysis of the phospho‐proteome, the quantification of phosphorylated peptides from the Spectronaut output table was collapsed using the plug‐in tool for Perseus (1.6.7.0) to annotate the exact position of the phospho sites. For this, phospho sites were aggregated using the linear model‐based approach and filtered for a localization probability > 0.75. The data were filtered to contain > 25% valid values across all samples. Raw intensities were log2 transformed and missing values were imputed based on a Gaussian normal distribution with a width of 0.3 and a downshift of 1.8. Overrepresentation analysis (ORA) of annotation terms was performed using the gprofiler2 package (organism: homo sapiens, user threshold: 0.05, databases: Reactome). Network representation of proteins with regulated sites was performed with the STRING app (1.5.1) in Cytoscape (3.7.2).

## Author contributions

**Lisa Schweizer:** Conceptualization; formal analysis; investigation; methodology; writing – original draft; writing – review and editing. **Tina Schaller:** Conceptualization; resources. **Maximilian Zwiebel:** Formal analysis; investigation; writing – review and editing. **Özge Karayel:** Formal analysis; investigation; methodology. **Johannes Bruno Müller:** Conceptualization; investigation; methodology. **Wen‐Feng Zeng:** Formal analysis. **Sebastian Dintner:** Resources. **Thierry M Nordmann:** Writing – review and editing. **Klaus Hirschbühl:** Resources. **Bruno Märkl:** Conceptualization; resources; supervision. **Rainer Claus:** Conceptualization; supervision; writing – original draft; writing – review and editing. **Matthias Mann:** Conceptualization; supervision; writing – original draft; writing – review and editing.

## Disclosure and competing interests statement

MM is an indirect investor of the Evosep company. All other authors declare no competing interests.

## For more information

- www.biochem.mpg.de/mann
- https://www.uk‐augsburg.de/einrichtungen/institute/institut‐fuer‐pathologie‐und‐molekulare‐diagnostik/ueberblick

## Supporting information



Appendix

Expanded View Figures PDF

Table EV1

Table EV2

Table EV3

Table EV4

Table EV5

Table EV6

Table EV7

Table EV8

Table EV9

Table EV10

Table EV11

Table EV12

Table EV13

Dataset EV1

Dataset EV2

Dataset EV3

Dataset EV4

Dataset EV5

Dataset EV6

PDF+

## Data Availability

Anonymized MS‐based proteomics data of this study have been deposited in the Mass Spectrometry Interactive Virtual Environment (MassIVE) of the ProteomeXchange consortium and are available via with identifier MSV000090983 (https://massive.ucsd.edu).
