# Supplementary material for: Quantitative multiorgan proteomics of fatal COVID‐19 uncovers tissue‐specific effects beyond inflammation
Source: EMBO Mol Med. 2023 Jul 31;15(9):e17459. doi: 10.15252/emmm.202317459 (PMC10493576; doi:10.15252/emmm.202317459)
Supplement: Supplementary file 1 — Appendix [file EMMM-15-e17459-s002.pdf]

Appendix

Appendix Fig S1 – Organ-wise evaluation of technical drift effects

Appendix Fig S2 – Comparison to previous proteomic profiling of COVID-19

Appendix Fig S3 – The organ-specific biology of COVID-19

Appendix Fig S4 – Post-mortem quality control

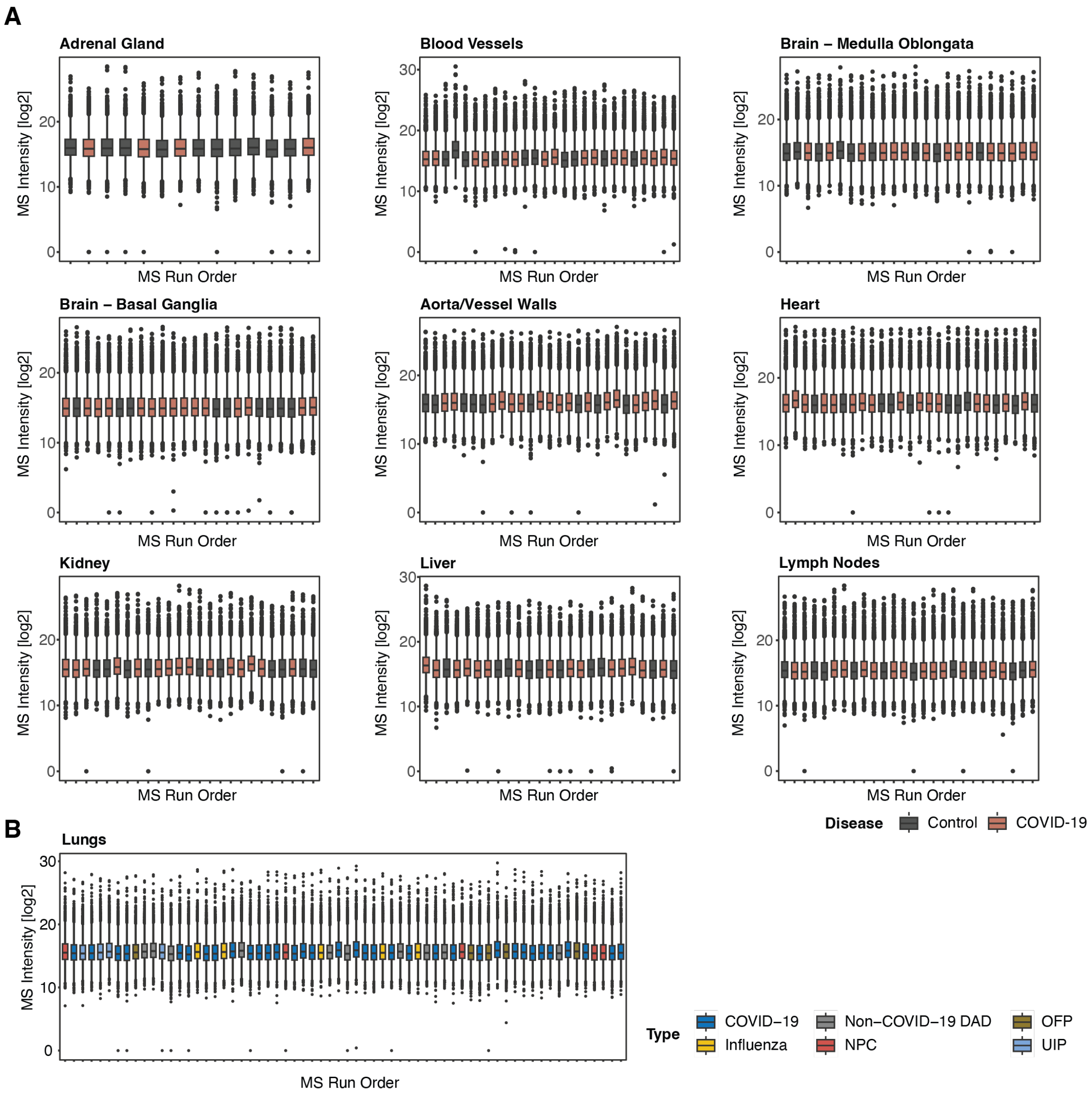

Appendix Fig S1 – Organ-wise evaluation of technical drift effects

A Log2 intensities plotted along the temporal axis of MS data acquisition for each organ. Samples of the COVID-19 and control group were randomized and colored accordingly. Boxes depict the inter quartile range (between 25th and 75th percentile) with the median intensity highlighted as bold lines, 95% of the data are contained within the lower and upper whiskers.

B Log2 intensities of the lungs as plotted in **A.** COVID-19 and diverse control groups were randomized and colored accordingly.

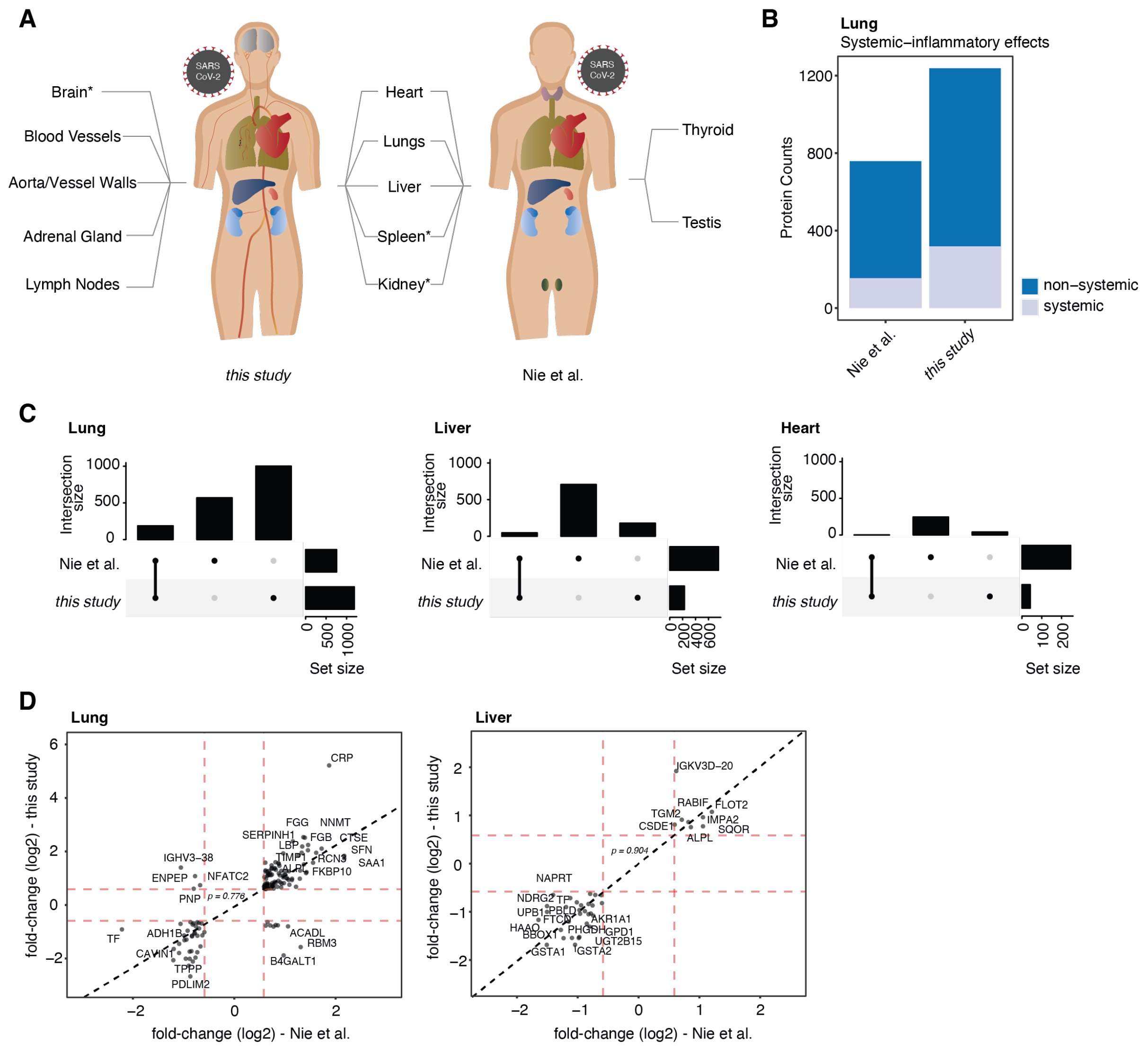

Appendix Fig S2 – Comparison to previous proteomic profiling of COVID-19

A Schematic comparison of investigated organs in a previous MS-based study of COVID-19 (Nie et al., 2021) and this work. Organs marked with an asterisk (*) were subset into physiological regions and were therefore excluded from the following comparison.

B Count of significantly differentially regulated proteins (t-test, adj.pval/q-va < 0.05, fold change >1.5) in both datasets stratified into systemic inflammatory and non-systemic proteins – a key strategy implemented in this study.

C Upset plots depicting the intersection of differentially expressed proteins compared between this study and previous work by Nie et al. for matching organs (the lungs, liver and heart).

D Comparison of fold changes for significantly differentially regulated proteins (t-test, adj.pval/q-val < 0.05, fold change >1.5, indicated by red dashed lines) between this study and Nie et al. Proteins in the lungs and liver scatter closely around the diagonal (dashed black line) indicating high correlation and common protein dysregulation.

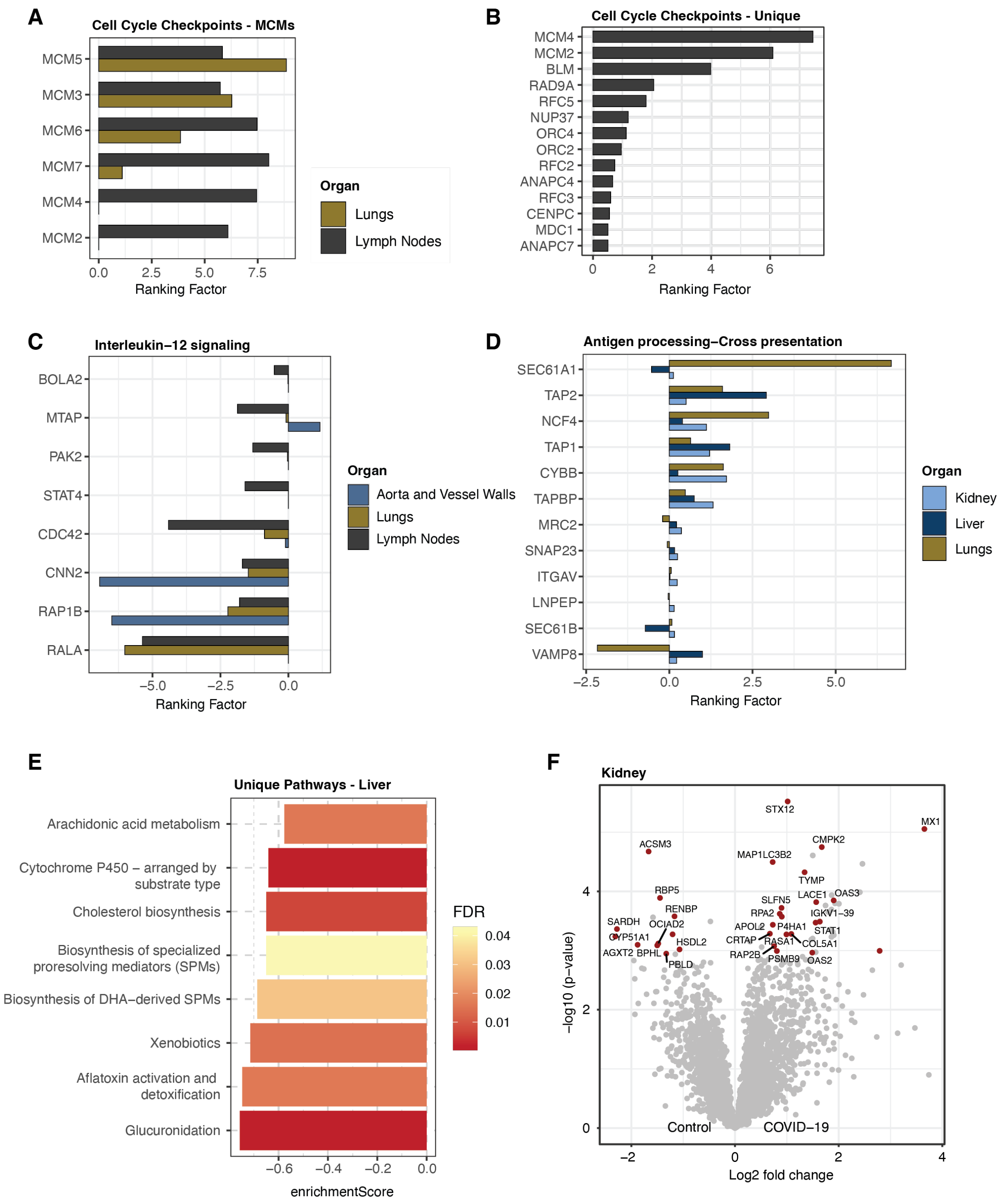

Appendix Fig S3 – The organ-specific biology of COVID-19

A Bar plot representing the ranking factor values (q-value (-log10) x fold change (log2)) of all MCM proteins associated to the Pathway term ‘Cell Cycle Checkpoints’ (Reactome database) commonly identified in lungs and lymph nodes.

B Ranking factor for proteins associated to the Pathway term ‘Cell Cycle Checkpoints’ (Reactome database) that have been uniquely identified in the lymph nodes.

C Proteins of Interleukin-12 signaling (Reactome database) in lungs, lymph nodes and aorta/vessel walls depicted by ranking factors.

D Bar plot of ranking factors for the pathway antigen processing-cross presentation (Reactome database) for lungs, liver and kidney. For reason of clarity, proteasomal subunits are excluded.

E Unique pathway terms for the liver which were identified using a GSEA enrichment (Reactome database). Color code of each bar indicate the FDR of the enrichment.

F Volcano plots of differential protein expression between COVID-19 control specimen in the kidney. Protein significance (t-test, q-val < 0.05, fold change >1.5) is highlighted in red.

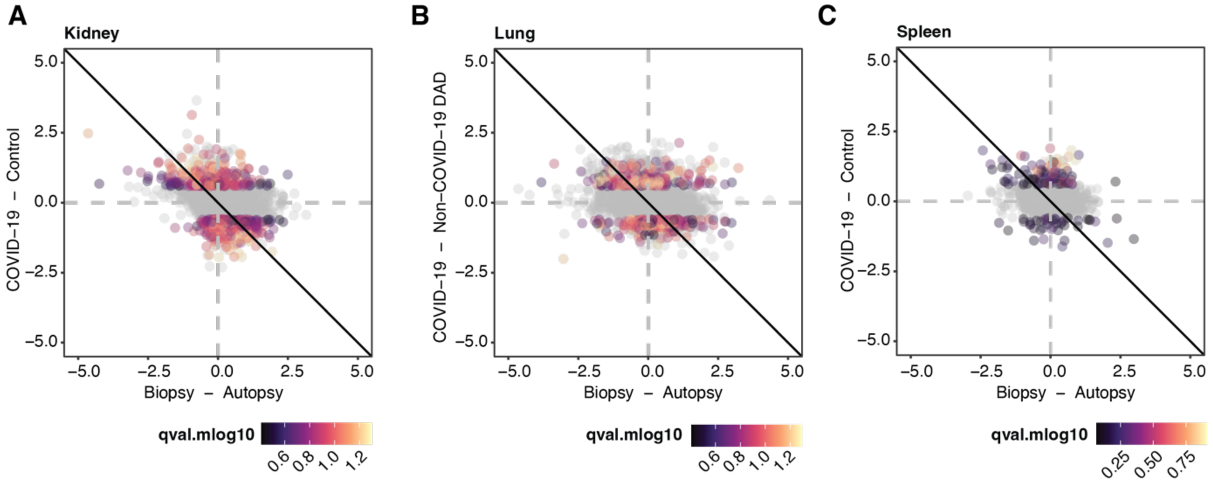

Appendix Fig S4 – Post-mortem quality control

A-C Evaluation of fold-change correlation comparing samples derived from biopsy/autopsy with COVID-19 and control samples (both n=3) for kidney (**A**), non-COVID-19 DAD in the lungs (**B**) and spleen (**C**). No proteins were significantly altered between biopsies and autopsies within the control groups (t-test, q-val < 0.05, fold change >1.5). Diagonal lines illustrate the directionality that would be expected if there was a correlation between alterations associated to COVID-19 and changes introduced by combining biopsies and autopsies. Notably, the distribution of data points in all tested organs did not correlate.
