# Supplementary material for: Quantitative multiorgan proteomics of fatal COVID‐19 uncovers tissue‐specific effects beyond inflammation
Source: EMBO Mol Med. 2023 Jul 31;15(9):e17459. doi: 10.15252/emmm.202317459 (PMC10493576; doi:10.15252/emmm.202317459)
Supplement: Supplementary file 2 — Expanded View Figures PDF [file EMMM-15-e17459-s023.pdf]

Expanded View Figures

Figure EV1. Mass spectrometry quality control.

A Comparison of protein identification in lung tissue of the COVID-19 cohort between the standardized 88 min gradient on Evosep One and a 60 or 120 min gradient on the EASY nanoLC system, respectively. Bars represent tissue samples of different patients and were grouped by the employed LC system, respectively.
B MS runtime of the comparison depicted in (B).
C Evaluation of our workflow for streamlined deparaffinization and lysis (APAC) and our previous state-of-art protocol for the processing of deparaffinized FFPE tissue in a 96-well format (Coscia *et al*, 2020). Each bar depicts tissue samples of different patients while being grouped by the tested workflow, respectively.
D Counts of quantified protein groups in COVID-19 samples and corresponding control groups in all organs using direct DIA data processing (not using a separately acquired DDA library). Each data point depicts a patient-derived sample as indicated in Table EV1.
E Extracted ion chromatograms and prediction of fragment intensities for two examples of SARS-CoV-2 peptides in the directDIA acquisition mode of the lungs. For both peptides, the same patient was selected to represent the cohort.

 © 2023 The Authors

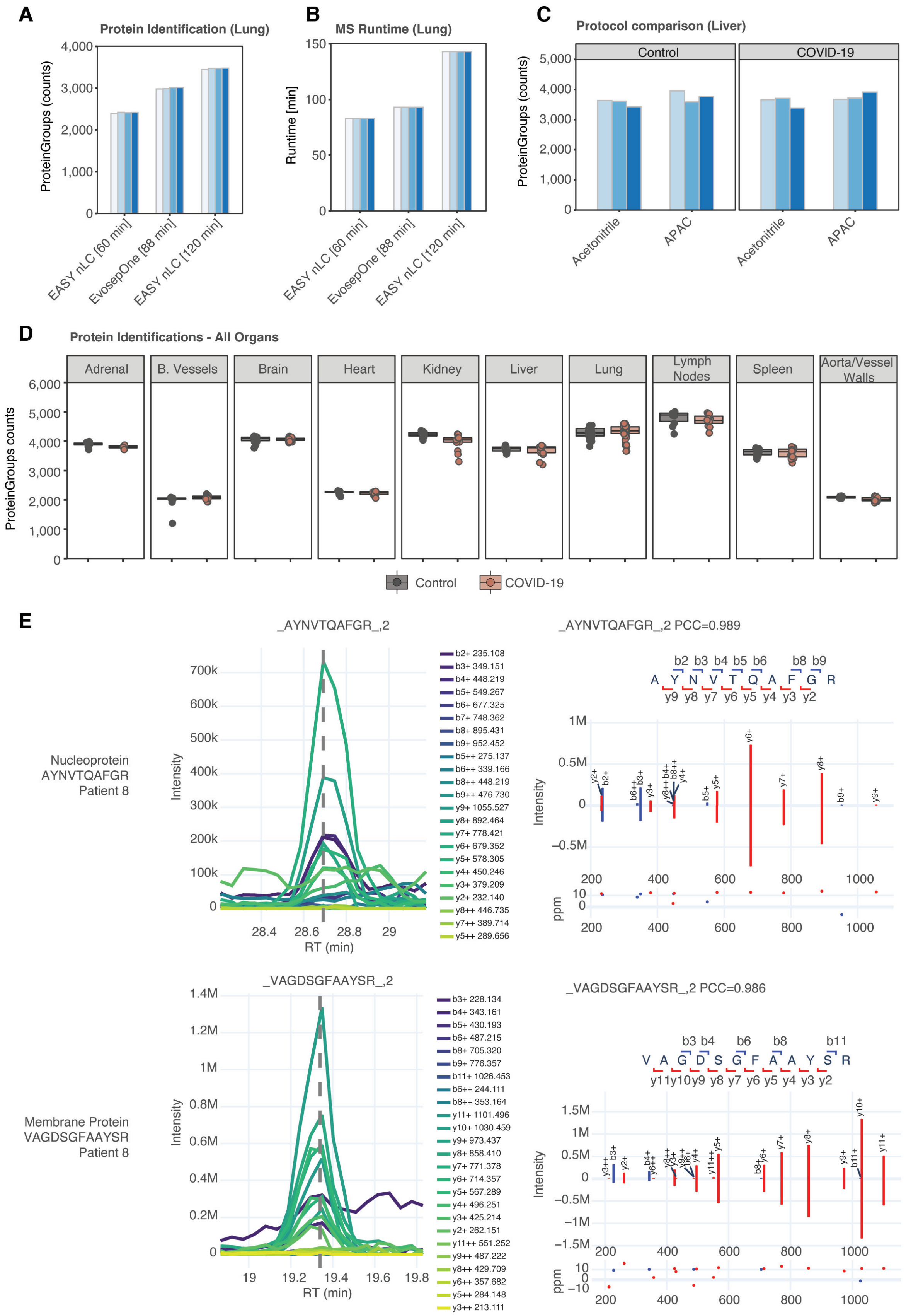
A
Protein Identification (Lung)
ProteinGroups (counts)
EASY nLC [60 min]
EvosepOne [88 min]
EASY nLC [120 min]
B
MS Runtime (Lung)
Runtime [min]
C
Protocol comparison (Liver)
Control
COVID-19
Acetonitrile
APAC
D
Protein Identifications - All Organs
Adrenal
B. Vessels
Brain
Heart
Kidney
Liver
Lung
Lymph Nodes
Spleen
Aorta/Vessel Walls
ProteinGroups counts
Control
COVID-19
E
AYNVTQAFGR,2
AYNVTQAFGR,2 PCC=0.989
Nucleoprotein
AYNVTQAFGR
Patient 8
Intensity
RT (min)
ppm
VAGDSGFAAYSR,2
VAGDSGFAAYSR,2 PCC=0.986
Membrane Protein
VAGDSGFAAYSR
Patient 8

Figure EV1.

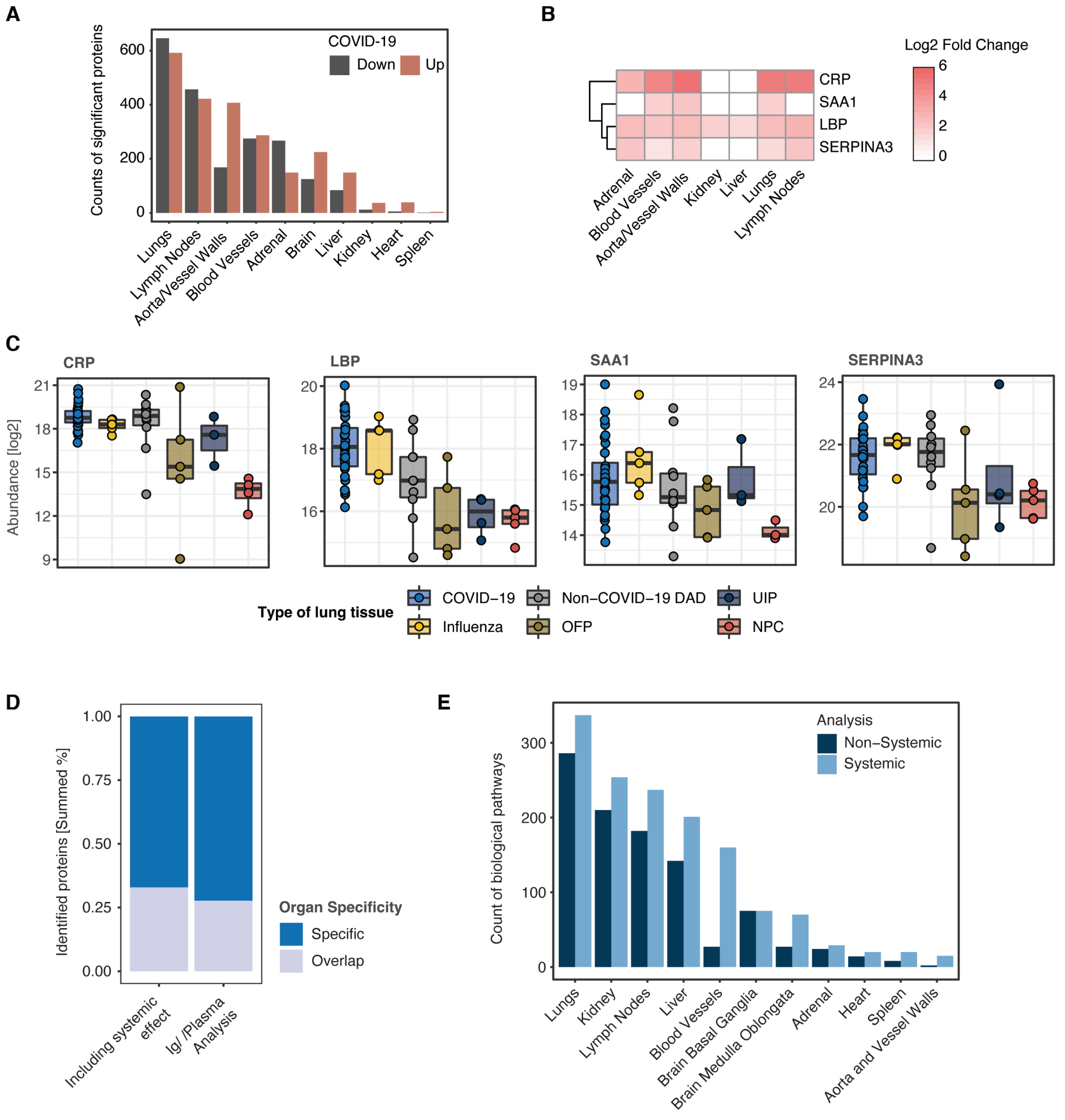

Figure EV2.

 © 2023 The Authors

Figure EV2. The systemic-inflammatory response in COVID-19.

A Number of significantly differentially regulated proteins (COVID-19 vs. controls) across all organs (minimum fold-change 1.5 at a q-value of <0.05).
B Fold changes (log2) of plasma-derived markers and predictive proteins for a severe progression of COVID-19.
C Boxplot representing the log2-abundance of the proteins shown in (B) for COVID-19 and similar pathologies of the lung which have been included into our study. Boxplots represent the 25- and 75-percentile with median values as central band; whiskers span the 1.5-fold interquartile range. Each data point depicts a patient-derived sample as indicated in Table EV1.
D Summed percentage of significantly differentially regulated proteins (minimum fold-change 1.5 at a q-value of <0.05) for the original proteome and after identification of the systemic effect, calculated for organ specificity and overlap in at least two organs, respectively.
E Count of biological pathways derived from an enrichment using the Reactome, KEGG and GO biological process databases and shown for differentially regulated proteins with association to the systemic and nonsystemic subset of proteins across all organs.

Figure EV3. Stratification of COVID-19 related pathologies in the lungs.

A Principal component analysis (PCA) of lung samples derived from COVID-19 and lung pathologies sharing similar phenotype features as well as the nonpathological control group.
B Upset plot depicting the intersection of differentially regulated proteins between COVID-19 and lung pathologies sharing similar phenotype features as well as the nonpathological control group.
C–F Differential regulation of phospho-sites derived from pairwise comparisons between COVID-19 and the control groups of the lungs: non-COVID-19 DAD (A), Influenza (B), fibrosing organizing pneumonia (OFP, C) and usual interstitial pneumonia (UIP, D). Protein significance (t-test, q-val < 0.05, fold change > 1.5) is highlighted in red.

© 2023 The Authors

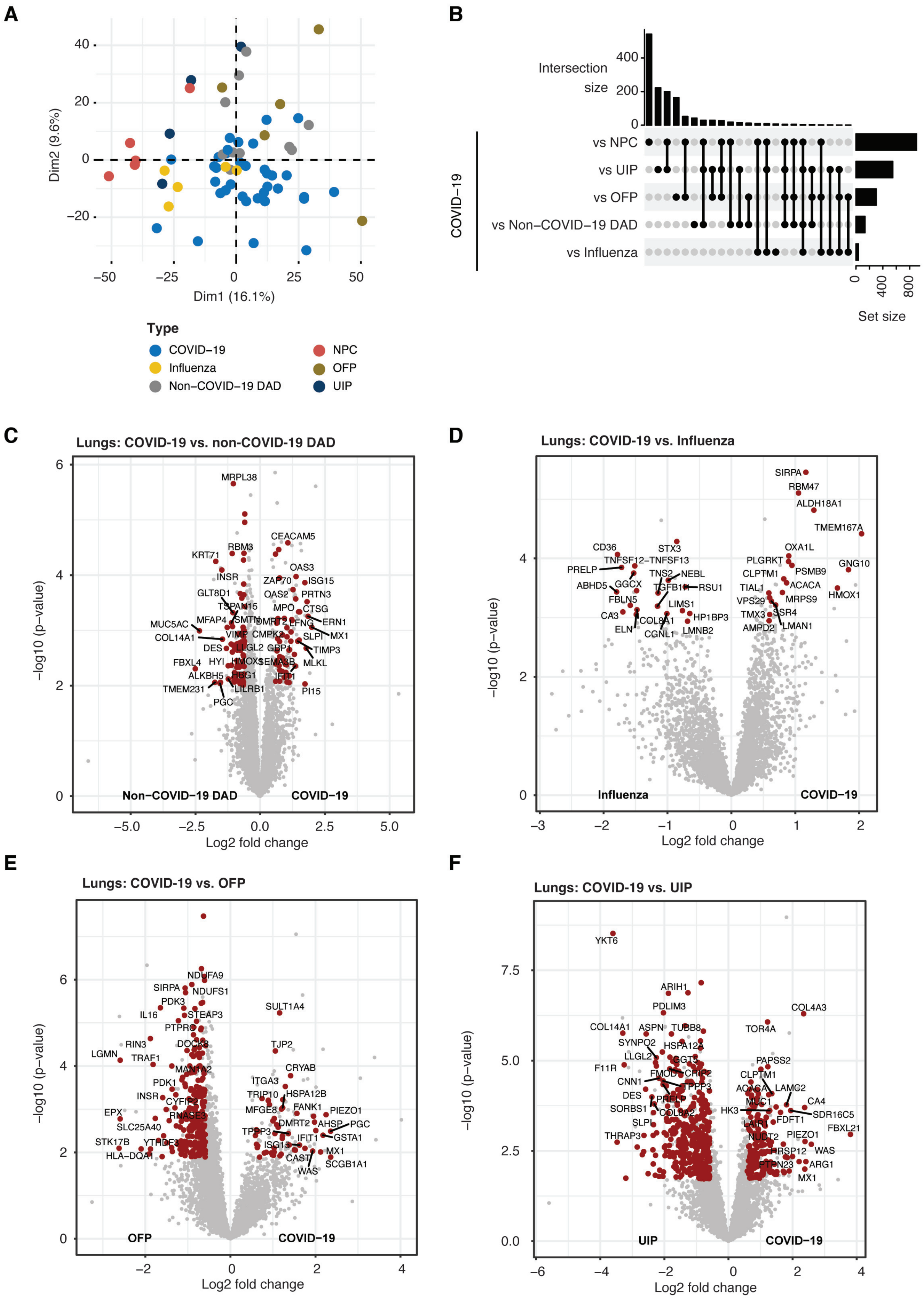

Figure EV3.

© 2023 The Authors

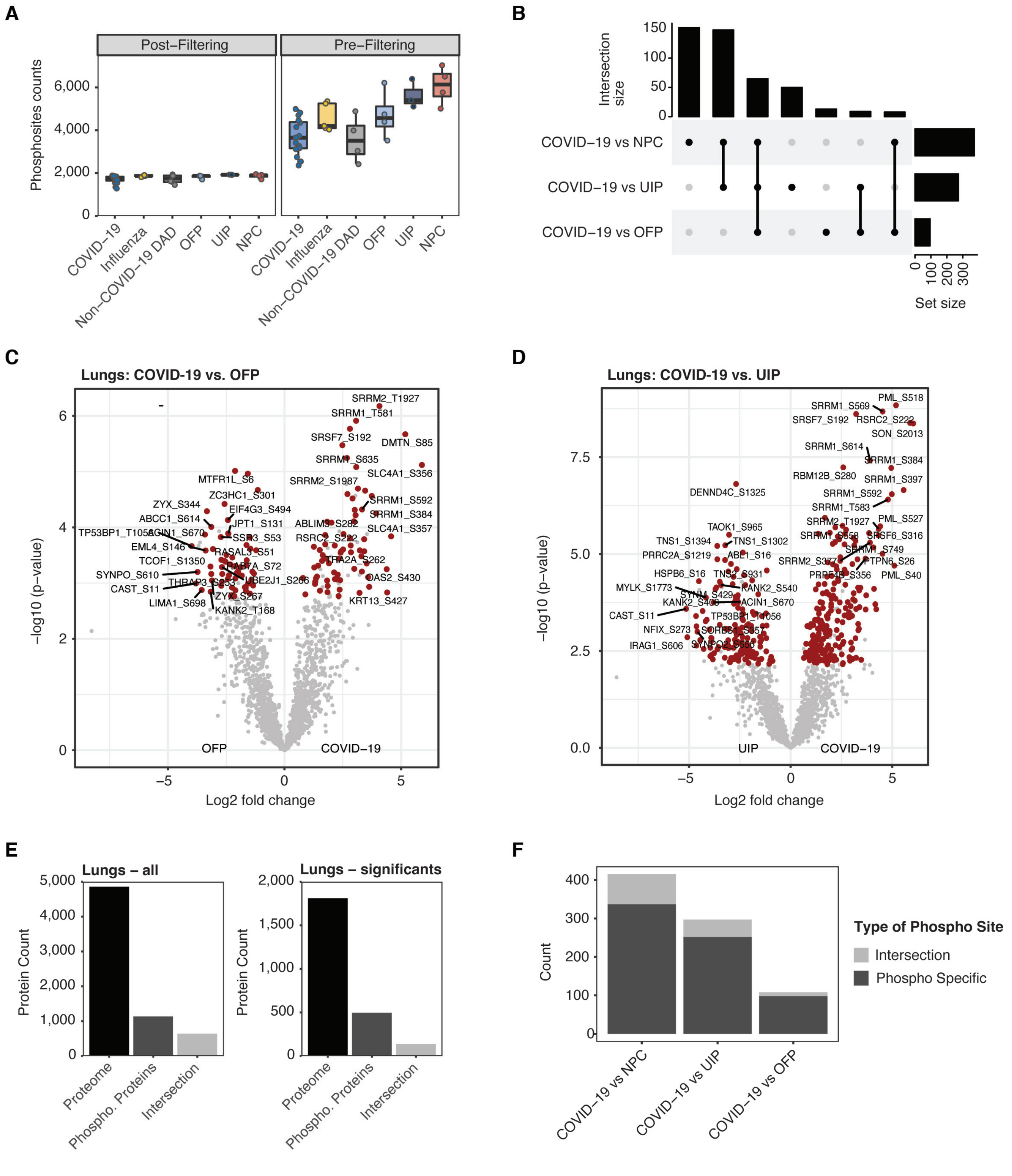

A
Post–Filtering
Pre–Filtering
Phosphosites counts
6,000
4,000
2,000
0
COVID–19
Influenza
Non–COVID–19 DAD
OFP
UIP
NPC
B
Intersection size
150
100
50
0
COVID–19 vs NPC
COVID–19 vs UIP
COVID–19 vs OFP
0
100
200
300
Set size
C
Lungs: COVID-19 vs. OFP
-log10 (p-value)
Log2 fold change
OFP
COVID–19
SRRM2_T1927
SRRM1_T581
SRSF7_S192
DMTN_S85
SRRM1_S635
SLC4A1_S356
MTFR1L_S6
SRRM2_S1987
ZC3HC1_S301
SRRM1_S592
ZYX_S344
EIF4G3_S494
SRRM1_S384
ABCC1_S614
JPT1_S131
ABLIM3_S282
SLC4A1_S357
RSRC2_S222
EML4_S146
TCOF1_S1350
TRA2A_S262
SYNPO_S610
OAS2_S430
CAST_S11
LIMA1_S698
KANK2_T168
KRT13_S427
D
Lungs: COVID-19 vs. UIP
-log10 (p-value)
Log2 fold change
UIP
COVID–19
PML_S518
SRRM1_S569
SRSF7_S192
RSRC2_S222
SON_S2013
SRRM1_S614
SRRM1_S384
RBM12B_S280
SRRM1_S397
DENND4C_S1325
SRRM1_S592
SRRM1_T583
PML_S527
TAOK1_S965
SRRM2_T1927
TNS1_S1394
TNS1_S1302
SRSF6_S316
PRRC2A_S1219
ABL1_S16
SRRM1_S749
TPN6_S26
HSPB6_S16
PML_S40
MYLK_S1773
KANK2_S540
CAST_S11
NFIX_S273
IRAG1_S606
E
Lungs – all
Lungs – significants
Protein Count
5,000
4,000
3,000
2,000
1,000
0
2,000
1,500
1,000
500
0
Proteome
Phospho. Proteins
Intersection
F
Count
400
300
200
100
0
COVID–19 vs NPC
COVID–19 vs UIP
COVID–19 vs OFP
Type of Phospho Site
Intersection
Phospho Specific

Figure EV4.

Figure EV4. Quantitative assessment of phospho-proteomic data.

A Counts of quantified phosphorylation sites in COVID-19 and all control groups. Data are shown before (right) and after (left) stringent filtering as described (Materials and Methods). Boxplots represent the 25- and 75-percentile with median values as central band; whiskers span the 1.5-fold interquartile range. Each data point depicts a patient-derived sample for COVID-19 (n = 16), Influenza (n = 5), Non-COVID-19 DAD (n = 4), OFP (n = 4), UIP (n = 3) and NPC (n = 4).

B Upset plot depicting the intersection of differentially expressed proteins across between COVID-19 and NPC, OFP or UIP, respectively. Statistical testing in comparison to Influenza and non-COVID-19 DAD did not result in significant hits.

C, D Differential phospho-site regulation between COVID-19 and OFP (C) or UIP (D), respectively. Protein significance (t-test, q-val < 0.05, fold change > 1.5) is highlighted in red.

E Intersection between phosphorylated proteins and full proteome in this dataset. Bars show count of identified proteins (black), phosphorylated sites summarized in protein groups (dark gray) and the overlap of the former ones (Intersection, light gray). The analysis for all proteins (left) and significantly differentially regulation (left) determined between all controls and COVID-19 in the lungs of this study show a low degree of corresponding proteomic and phospho-proteomic information.

F Count of phospho-sites separated into unique differential alteration on the level of protein phosphorylation (Phospho Specific) compared to the portion of sites which correspond to significant differential expression on the protein level (Intersection).

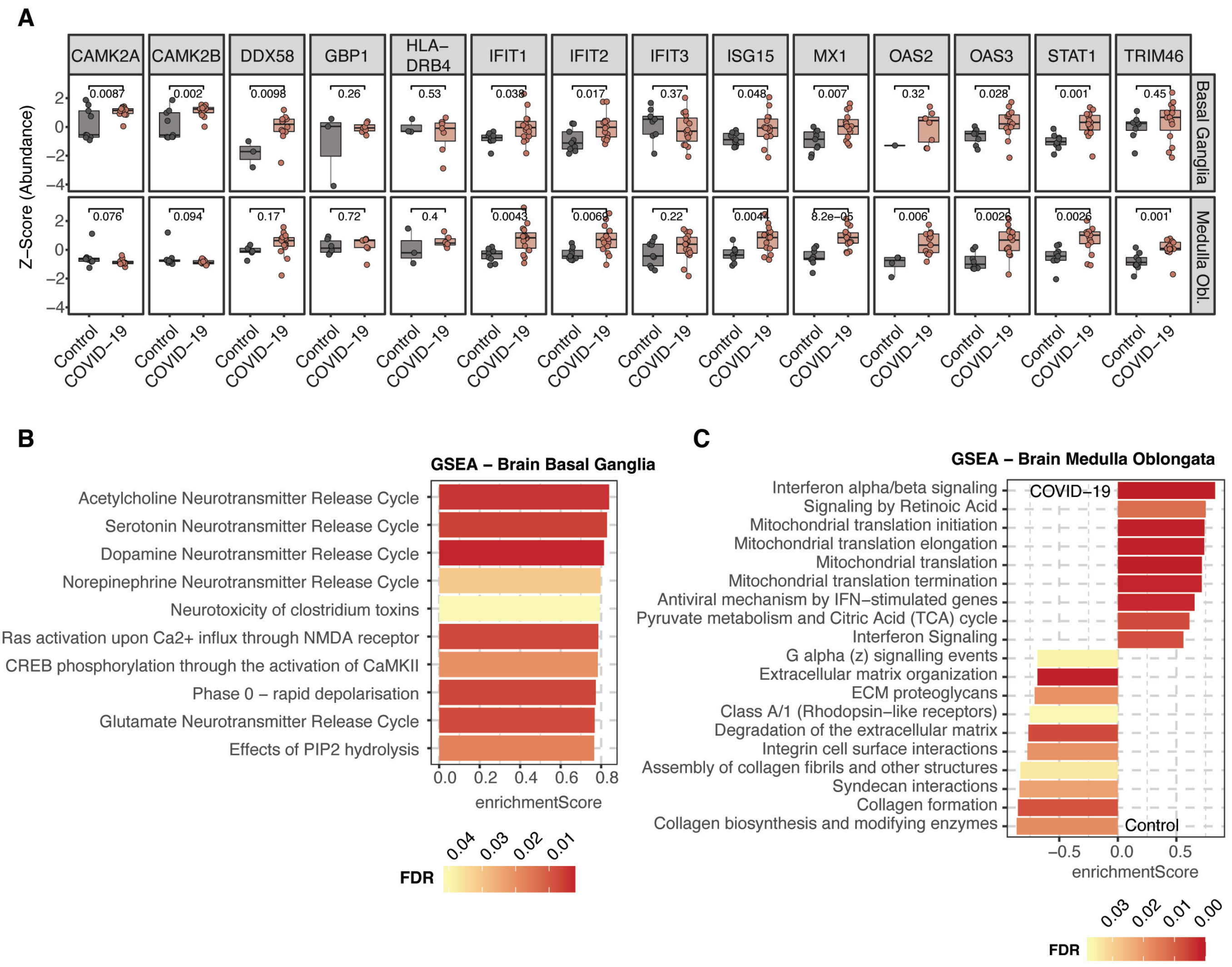

Figure EV5.

 © 2023 The Authors

◀ **Figure EV5. Biological processes associated with COVID-19 of the brain.**

A *Z*-Score protein abundance of proteins associated with "Interferon Signaling" (Reactome database) that showed overlapping occurrence in both regions of the brain. An assessment of statistical significance (unpaired *t*-test) between COVID-19 and control samples is annotated for each comparison. Boxplots represent the 25- and 75-percentile with median values as central band; whiskers span the 1.5-fold interquartile range. Each data point depicts a patient-derived sample as indicated in Table EV1.

B, C GSEA enrichment depicted by the top 10 most significant terms assigned by enrichment score. A positive enrichment score indicates positive protein abundance in COVID-19, whereas a negative value indicates an enrichment in the control samples. The color coding of all bars depicts the FDR value of the respective enrichment term.

© 2023 The Authors
