# Supplementary material for: Quantitative multiorgan proteomics of fatal COVID‐19 uncovers tissue‐specific effects beyond inflammation
Source: EMBO Mol Med. 2023 Jul 31;15(9):e17459. doi: 10.15252/emmm.202317459 (PMC10493576; doi:10.15252/emmm.202317459)
Supplement: Supplementary file 3 — Table EV1 [file EMMM-15-e17459-s015.docx]

**Table EV1 – Design of the COVID-19 and control cohort**

| ***Tissue*** | ***Type*** | ***#Patients (#Samples from histological phenotypes)*** |
| --- | --- | --- |
| Lungs | COVID-19 | 19 (35) |
|  | Control - NPC | 5 |
|  | Control - UIP | 4 |
|  | Control - OFP | 5 |
|  | Control – Non-COVID-19 DAD | 6 (10) |
|  | Control - Influenza | 5 |
| Adrenal Gland | COVID-19 | 4 |
|  | Control | 10 |
| Blood Vessel | COVID-19 | 16 |
|  | Control | 10 |
| Brain – Medulla Oblongata | COVID-19 | 15 |
|  | Control | 9 |
| Brain – Basal Ganglia | COVID-19 | 15 |
|  | Control | 9 |
| Heart | COVID-19 | 16 |
|  | Control | 10 |
| Kidney | COVID-19 | 14 |
|  | Control | 10 |
| Liver | COVID-19 | 15 |
|  | Control | 10 |
| Lymph Nodes | COVID-19 | 16 |
|  | Control | 10 |
| Spleen | COVID-19 | 15 |
|  | Control | 10 |
| Aorta/Vessel Walls | COVID-19 | 17 |
|  | Control | 10 |
|  | Total #samples | 305 |
|  | **Derived from**:  Total #COVID-19 patients | 19 |
|  | Total #control patients | 85 |
