# Supplementary material for: Quantitative multiorgan proteomics of fatal COVID‐19 uncovers tissue‐specific effects beyond inflammation
Source: EMBO Mol Med. 2023 Jul 31;15(9):e17459. doi: 10.15252/emmm.202317459 (PMC10493576; doi:10.15252/emmm.202317459)
Supplement: Supplementary file 4 — Table EV2 [file EMMM-15-e17459-s018.docx]

**Table EV2 - Basic clinical patient characteristics of the COVID-19 cohort**

| ***Variable*** | ***No.*** |
| --- | --- |
| Total number of patients | 19 (100%) |
| Mean Age (range) | 74 years (57–90) |
| Sex (male/female) | 14 (74%) / 5 (26%) |
| Smoker (yes/no) | 7 (37%) /12 (63%) |
| Comorbidities | |
| Median number (range) | 4 (0–9) |
| *Cardiovascular comorbidities* | 13 (68%) |
| Atrial fibrillation | 11 (58%) |
| Coronary artery disease | 5 (26%) |
| Cardiomyopathy | 5 (26%) |
| Aortic valve stenosis | 1 (5%) |
| Hypertension | 13 (68%) |
| Arteriosclerosis | 9 (47%) |
| *Metabolic comorbidities* | 11 (58%) |
| Diabetes | 6 (32%) |
| Obesity | 9 (47%) |
| Median body mass index (range) | 28.3 kg/m² (19.6 – 66.2) |
| *Chronic respiratory disease* | 5 (26%) |
| Chronic renal disease | 7 (37%) |
| Hyperlipoproteinemia | 4 (21%) |
| *Prevalent malignancies* | 3 (16%) |
| *Therapeutics* |  |
| Angiotensin converting enzyme inhibitors | 9 (47%) |
| Angiotensin II receptor blockers | 2 (11%) |

CLL = chronic lymphatic leukemia, CMML = chronic myelomonocytic leukemia
