# Supplementary material for: Quantitative multiorgan proteomics of fatal COVID‐19 uncovers tissue‐specific effects beyond inflammation
Source: EMBO Mol Med. 2023 Jul 31;15(9):e17459. doi: 10.15252/emmm.202317459 (PMC10493576; doi:10.15252/emmm.202317459)
Supplement: Supplementary file 5 — Table EV3 [file EMMM-15-e17459-s010.docx]

**Table EV3 - Clinical course and treatment of the COVID-19 cohort**

| ***Variable*** | ***No.*** |
| --- | --- |
| COVID-19 testing: nasopharyngeal swab | 19 (100%) |
| Symptoms on admission | |
| Cough | 13 (68%) |
| Dyspnea | 11 (58%) |
| Fever | 9 (47%) |
| Worsening general condition | 9 (47%) |
| Diarrhea | 1 (5%) |
| Median time between onset of symptoms and admission* (range) | 5 days (0 – 21) |
| Radiologic findings (CT = 4, X-Ray = 15) | |
| Bilateral patchy shadowing | 16 (84%) |
| Ground-glass opacity | 2 (11%) |
| Respiratory findings | |
| Median Horowitz index* (range)) | 107 mmHg (50 – 150) |
| Acute respiratory distress syndrome | 16 (84%) |
| Septic shock | 7 (37%) |
| Acute kidney injury | 14 (74%) |
| Congestive heart failure | 13 (68%) |
| Clinical diagnosed pneumonia | 8 (42%) |
| Thromboembolic events (DVT/PE) | 2 (11%) |
| Systemic therapy | |
| Systemic glucocorticoids (hydrocortisone for septic shock) | 3 (16%) |
| Remdesivir | 0 (0%) |
| Chloroquine or Hydroxychloroquine | 8 (42%) |
| Reconvalescent plasma | 3 (16%) |
| Intravenous antibiotics | 17 (89%) |
| Median number of antibiotic substances (range) | 2 (0 – 8) |
| Anticoagulation use | |
| Deep venous thrombosis prophylaxis | 9 (47%) |
| Full dose anticoagulation | 10 (53%) |
| Vasopressors | 10 (53%) |
| Ventilation | |
| Oxygen only | 5 (26%) |
| Noninvasive | 5 (26%) |
| Invasive | 9 (47%) |
| Renal replacement therapy | 5 (26%) |

*Missing information in three cases

CT = computed tomography, DVT = deep vein thrombosis, PE = pulmonary embolism
