# Supplementary material for: Quantitative multiorgan proteomics of fatal COVID‐19 uncovers tissue‐specific effects beyond inflammation
Source: EMBO Mol Med. 2023 Jul 31;15(9):e17459. doi: 10.15252/emmm.202317459 (PMC10493576; doi:10.15252/emmm.202317459)
Supplement: Supplementary file 10 — Table EV8 [file EMMM-15-e17459-s004.docx]

**Table EV8 - Specific clinical characteristics of the heart – COVID-19 and controls**

| ***Variable*** | ***COVID-19*** | | ***Controls*** | |
| --- | --- | --- | --- | --- |
|  | number | percentage/range | number | percentage/range |
| Total number of patients | 19 | 100% | 11 | 100% |
| Median age (range) | 73 | 57-90 | 75 | 45-83 |
| Sex (male/female) | 14/5 | 74%/26% | 8/3 | 73%/27% |
| Smoker (yes/no/NA) | 7/12/0 | 37%/63%/0% | 3/7/1 | 27%/64%/9% |
| Biopsy/autopsy | 0/19 | 0%/100% | 0/11 | 0%/100% |
| *Cardiovascular comorbidities* |  |  |  |  |
| atrial flutter/fibrillation | 11 | 58% | 2 | 18% |
| hypertension | 13 | 68% | 6 | 55% |
| coronary artery disease | 5 | 26% | 5 | 45% |
| cardiomyopathy | 5 | 26% | 3 | 27% |
| arteriosclerosis | 9 | 47% | 6 | 55% |
| *Metabolic comorbidities* |  |  |  |  |
| diabetes | 6 | 32% | 5 | 45% |
| hyperlipidemia/  hypercholesterinemia | 4 | 21% | 0 | 0% |
| obesity [BMI] | 9 | 47% | 2 | 18% |
| BMI [kg/m^2^] | 28.3 | 19.6-66.2 | 25.9 | 21.7-58.4 |
| *Chronic respiratory disease* | 5 | 26% | 3 | 30% |
| *Chronic renal disease* | 7 | 37% | 3 | 30% |
| *Active malignancies* | 3 | 16% | 4 | 40% |
