# Supplementary material for: Quantitative multiorgan proteomics of fatal COVID‐19 uncovers tissue‐specific effects beyond inflammation
Source: EMBO Mol Med. 2023 Jul 31;15(9):e17459. doi: 10.15252/emmm.202317459 (PMC10493576; doi:10.15252/emmm.202317459)
Supplement: Supplementary file 23 [file EMMM-15-e17459-s001.pdf]

EMBO Molecular Medicine

Quantitative multi-organ proteomics of COVID-19 uncovers tissue-specific effects beyond inflammation

Lisa Schweizer, Tina Schaller, Maximilian Zwiebel, Özge Karayel, Johannes Müller, Wen-Feng Zeng, Sebastian Dintner, Thierry Nordmann, Klaus Hirschbühl, Bruno Märkl, Rainer Claus, and Matthias Mann
DOI: 10.15252/emmm.202317459

Corresponding authors: Matthias Mann (mmann@biochem.mpg.de) , Rainer Claus (Rainer.Claus@uk-augsburg.de)

Review Timeline:

Submission Date:	20th Jan 23
Editorial Decision:	20th Feb 23
Revision Received:	19th May 23
Editorial Decision:	22nd Jun 23
Revision Received:	11th Jul 23
Accepted:	14th Jul 23

Editors: Jingyi Hou / Zeljko Durdevic

Transaction Report:

(Note: With the exception of the correction of typographical or spelling errors that could be a source of ambiguity, letters and reports are not edited. Depending on transfer agreements, referee reports obtained elsewhere may or may not be included in this compilation. Referee reports are anonymous unless the Referee chooses to sign their reports.)

20th Feb 2023

Dear Matthias,

Thank you again for submitting your work to EMBO Molecular Medicine. We have heard back from three referees who agreed to evaluate your manuscript. As you will see from the reports below, the referees acknowledge the interest of the study. However, they raise a series of concerns, which we would ask you to address in a major revision of the manuscript.

I think that the referees' recommendations are relatively straightforward, so there is no need to reiterate their comments. In particular, Referees #2 and #3 raised concerns regarding the limited information about the control group, and Referee #2 expressed concern that this could potentially lead to misinterpretation of the results. Thus, this issue must be carefully addressed. To address Referee #2's comment regarding Nie et al, 2021, it is important to better contextualize the findings in relation to existing literature and emphasize the novelty of the current study. During our pre-decision cross-commenting process (in which the referees are given a chance to make additional comments, including on each other's reports), Referee #2 suggested including a comparison with the proteome data in Nie et al 2021, which we would encourage you to conduct.

All other issues raised by the referees need to be satisfactorily addressed as well. Please feel free to contact me if you would like to discuss any of the issues raised by the reviewers in further detail.

We would welcome the submission of a revised version within three months. Please note that EMBO Molecular Medicine strongly supports a single round of revision and that, as acceptance or rejection of the manuscript will depend on another round of review, your responses should be as complete as possible.

EMBO Molecular Medicine has a "scooping protection" policy, whereby similar findings that are published by others during review or revision are not a criterion for rejection. Should you decide to submit a revised version, I do ask that you get in touch after three months if you have not completed it to update us on the status.

Please also contact us as soon as possible if similar work is published elsewhere. If other work is published we may not be able to extend the revision period beyond three months.

Please read below for important editorial formatting and consult our author's guidelines for proper formatting of your revised article for EMBO Molecular Medicine.

I look forward to receiving your revised manuscript.

Kind regards,
Jingyi

Jingyi Hou
Editor
EMBO Molecular Medicine

*****

When submitting your revised manuscript, please carefully review the instructions that follow below. We perform an initial quality control of all revised manuscripts before re-review; failure to include requested items will delay the evaluation of your revision.

We require:

1) A .docx formatted version of the manuscript text (including legends for main figures, EV figures and tables). Please make sure that the changes are highlighted to be clearly visible.

2) Individual production quality figure files as .eps, .tif, .jpg (one file per figure). For guidance, download the 'Figure Guide PDF': (https://www.embopress.org/page/journal/17574684/authorguide#figureformat).

3) A .docx formatted letter INCLUDING the reviewers' reports and your detailed point-by-point responses to their comments. As

part of the EMBO Press transparent editorial process, the point-by-point response is part of the Review Process File (RPF), which will be published alongside your paper.

4) A complete author checklist, which you can download from our author guidelines (https://www.embopress.org/page/journal/17574684/authorguide#submissionofrevisions). Please insert information in the checklist that is also reflected in the manuscript. The completed author checklist will also be part of the RPF.

5) Please note that all corresponding authors are required to supply an ORCID ID for their name upon submission of a revised manuscript.

6) It is mandatory to include a 'Data Availability' section after the Materials and Methods. Before submitting your revision, primary datasets produced in this study need to be deposited in an appropriate public database, and the accession numbers and database listed under 'Data Availability'. Please remember to provide a reviewer password if the datasets are not yet public (see https://www.embopress.org/page/journal/17574684/authorguide#dataavailability).

In case you have no data that requires deposition in a public database, please state so in this section. Note that the Data Availability Section is restricted to new primary data that are part of this study.

7) For data quantification: please specify the name of the statistical test used to generate error bars and P values, the number (n) of independent experiments (specify technical or biological replicates) underlying each data point and the test used to calculate p-values in each figure legend. The figure legends should contain a basic description of n, P and the test applied. Graphs must include a description of the bars and the error bars (s.d., s.e.m.). See also 'Figure Legend' guidelines: https://www.embopress.org/page/journal/17574684/authorguide#figureformat

8) At EMBO Press we ask authors to provide source data for the main manuscript figures. Our source data coordinator will contact you to discuss which figure panels we would need source data for and will also provide you with helpful tips on how to upload and organize the files.

9) Our journal encourages inclusion of *data citations in the reference list* to directly cite datasets that were re-used and obtained from public databases. Data citations in the article text are distinct from normal bibliographical citations and should directly link to the database records from which the data can be accessed. In the main text, data citations are formatted as follows: "Data ref: Smith et al, 2001" or "Data ref: NCBI Sequence Read Archive PRJNA342805, 2017". In the Reference list, data citations must be labeled with "[DATASET]". A data reference must provide the database name, accession number/identifiers and a resolvable link to the landing page from which the data can be accessed at the end of the reference. Further instructions are available at .

10) We replaced Supplementary Information with Expanded View (EV) Figures and Tables that are collapsible/expandable online. A maximum of 5 EV Figures can be typeset. EV Figures should be cited as 'Figure EV1, Figure EV2" etc... in the text and their respective legends should be included in the main text after the legends of regular figures.

- For the figures that you do NOT wish to display as Expanded View figures, they should be bundled together with their legends in a single PDF file called *Appendix*, which should start with a short Table of Content. Appendix figures should be referred to in the main text as: "Appendix Figure S1, Appendix Figure S2" etc.

- Additional Tables/Datasets should be labeled and referred to as Table EV1, Dataset EV1, etc. Legends have to be provided in a separate tab in case of .xls files. Alternatively, the legend can be supplied as a separate text file (README) and zipped together with the Table/Dataset file.

See detailed instructions here:

.

11) The paper explained: EMBO Molecular Medicine articles are accompanied by a summary of the articles to emphasize the major findings in the paper and their medical implications for the non-specialist reader. Please provide a draft summary of your article highlighting

- the medical issue you are addressing,

- the results obtained and

- their clinical impact.

This may be edited to ensure that readers understand the significance and context of the research. Please refer to any of our

published articles for an example.

12) For more information: There is space at the end of each article to list relevant web links for further consultation by our readers. Could you identify some relevant ones and provide such information as well? Some examples are patient associations, relevant databases, OMIM/proteins/genes links, author's websites, etc...

13) Author contributions: You will be asked to provide CRediT (Contributor Role Taxonomy) terms in the submission system. These replace a narrative author contribution section in the manuscript.

14) A Conflict of Interest statement should be provided in the main text.

15) Every published paper now includes a 'Synopsis' to further enhance discoverability. Synopses are displayed on the journal webpage and are freely accessible to all readers. They include a short stand first (maximum of 300 characters, including space) as well as 2-5 one-sentences bullet points that summarizes the paper. Please write the bullet points to summarize the key NEW findings. They should be designed to be complementary to the abstract - i.e. not repeat the same text. We encourage inclusion of key acronyms and quantitative information (maximum of 30 words / bullet point). Please use the passive voice. Please attach these in a separate file or send them by email, we will incorporate them accordingly.

Please also suggest a striking image or visual abstract to illustrate your article as a PNG file 550 px wide x 300-600 px high.

EMBO Molecular Medicine has a "scooping protection" policy, whereby similar findings that are published by others during review or revision are not a criterion for rejection. Should you decide to submit a revised version, I do ask that you get in touch after three months if you have not completed it, to update us on the status.

Please note: When submitting your revision you will be prompted to enter your funding and payment information. This will allow Wiley to send you a quote for the article processing charge (APC) in case of acceptance. This quote takes into account any reduction or fee waivers that you may be eligible for. Authors do not need to pay any fees before their manuscript is accepted and transferred to the publisher.

EMBO Press participates in many Publish and Read agreements that allow authors to publish Open Access with reduced/no publication charges. Check your eligibility: https://authorservices.wiley.com/author-resources/Journal-Authors/open-access/affiliation-policies-payments/index.html

***** Reviewer's comments *****

Referee #1 (Comments on Novelty/Model System for Author):

I have some questions about the data normalization (see full review).

Referee #1 (Remarks for Author):

Schweizer et al have performed proteomic analysis of hundreds of COVID-19-infected tissues from post-mortem autopsies across several major human organ systems. The authors have also conducted proteomic analysis of samples from other lung diseases (e.g., influenza, diffuse alveolar damage) and non-pathological controls. This work is of great importance given that proteomics has been applied much less frequently than transcriptomic approaches to COVID-19 due to the inherent technological challenges of proteomics. The work appears thorough and well done. Although the work is purely descriptive and can sometimes read like a "parts list", I agree with the authors that the value of this work is that it may "[give] rise to new hypotheses that can be validated or used in translational context by the community."

Major points:
- The authors have focused primarily on organ-specific signatures between COVID-19 and control samples. Have the authors considered patient-specific signatures, especially ones that might be conserved across organ systems?
- There is considerable variability in the COVID patients in Fig. 3C. For example, several COVID patients have Cluster 6 proteins upregulated even though this cluster is marked by "proteins that decreased in abundance compared to NPC controls." Can the authors analyze or speculate on the heterogeneity observed across patients?
- It is unclear if any of the lung phospho-proteomes (Fig. 4) are from the same the samples as the lung proteomes (Fig. 3). If they are from some of the same samples, can any of the observed changes in phospho-sites be normalized to changes in protein expression level?
- can the authors use the phospho-proteomic data (Fig. 4) to infer which kinases are most upregulated in COVID? For example, methods like Post-Translational Modification Set Enrichment Analysis (PMID: 30563849).
- With such a large sample cohort, did the authors need to do any batch correction of the data? They mention "computer-based randomization of samples ... was used to exclude batch effects during sample preparation." but this doesn't exclude batch

effects as the samples were run on the LC-MS.
- I didn't find any details about normalization of the data in the Methods. Were the raw protein intensities simply log2 transformed? Did the authors investigate the effects of data normalization strategies across all the samples (i.e., simple methods like median subtraction or more complex methods like singular value decomposition such as EigenMS)?
- page 26: the authors should clarify this statement: "In both regions we quantified more than 4,000 protein groups that showed equal levels in both areas of COVID-19 and control specimen (Fig 6B)." According to the legend, it's actually the "Number of identified protein groups" that were equal, not the levels (i.e., which for me levels = protein expression levels).
- The Supplemental Tables of the proteomic data do not include data from the individual samples. There is only summary data (e.g., log2 fold change of protein phosphorylation in the lungs). The data would be much more useful is the authors included the data from the individual samples as well.
- The authors have analyzed samples from the very early phases of the COVID-19 pandemic (April to May 2020). Can the authors speculate on how the emergence of new COVID-19 variants might change their results?

Minor points:
- Typo, Fig 1 legend: "A total of 352 specimen from"
- Typo, page 10: "This revealed that markers of the RBC panel dominated some specimen of the COVID-19 cohort"
- Typo, Fig. 5 legend: "Differential protein expression between COVID-19 control specimen in the lymph nodes"

Referee #2 (Comments on Novelty/Model System for Author):

Novelty is reduced by publication of the paper by Nie et al. 2021. The model system is inadequate at this stage because there is insufficient information about the control subjects to evaluate claims that proteomic differences between COVID-19 patients and controls are indeed due to COVID rather than unrelated differences between patients and controls. Controls may be fine, but we just do not know.

Referee #2 (Remarks for Author):

The manuscript by Schweizer et al. describes development (actually improvement of current protocols) of a streamlined LC-MS/MS based clinical proteomic method and subsequent study of 352 FFPE tissue samples from 10 different organs of 19 COVID-19 patients who died from the disease and 9-10 (most organs) or more (26 lung) control samples. The depth of coverage from FFPE samples given relatively rapid analysis times (about 90 mins/sample) is impressive, making interesting clinical studies such as this one possible. The depth of coverage enabled the discovery of tissue-specific differences between COVID-19 patients and controls that went beyond the predominant circulating inflammatory molecules. While this work represents an impressive demonstration of cutting-edge clinical proteomics technology, unfortunately lack of information about controls limits the utility of clinical insights from the differences in protein amounts between samples. In addition, there are no follow-up experiments to test hypotheses generated by the data or to compensate for problems with the controls.

Specific Comments:

1) Info about COVID-19 patients is given in Supplementary Tables 1 and 2 but I cannot find any information about control individuals. This is a crucial omission, as we have no information about how well these individuals were matched for age, gender, and other health parameters. How can we know which differences are due to COVID-19 and which differences are due to other factors such as age or comorbidities between groups? Were these tissue differences, especially in lung, pre-existing, thus making those individuals more susceptible to death by COVID-19? Specifically, could the COVID patients have DAD as a pre-condition that rendered them susceptible rather than developing DAD-like changes as a result of COVID infection? / (i.e. cause and effect difficult to determine). Neuronal changes could also be an effect of age, but we cannot evaluate this possibility without information about controls. Lack of info about controls is especially important given the fact that COVID fatalities occur most frequently in older patients, especially those with underlying morbidities.

2) The in-depth proteomic study by Nie et al. of 7 organs from 19 COVID-19 patients, cited in this manuscript, used a TMT approach and also reported non-inflammatory tissue differences and as a result reduces the clinical novelty of the manuscript by Schweizer et al.

3) Filtering out circulation-mediated proteome changes helped to highlight organ-specific effects and is a good idea.

4) It's not clear to me whether or not phosphorylation levels on individual sites were corrected for total amounts of the corresponding proteins in the phosphoproteomics experiments described in Fig 4. I don't believe so, but in that case protein levels rather than phosphorylation status of each protein would then be the key variable. For example, NPC, UIP and OFP which had the greatest phospoproteomic differences also had the largest protein differences (page 18).

Minor Comments:

1) Throughout the manuscript supplementary figures are referred to as EV figures- this should be changed to make figure nomenclature consistent.

2) Supplemental Figure 1 A, B, and C: Presumably each bar represents a single sample/patient, this should be stated in the figure legend.

3) Page 19 line 2: should be "on average"

Referee #3 (Comments on Novelty/Model System for Author):

State of the art proteomics methods applied to a unique cohort, no direct medical applications, but of general scientific interest.

Referee #3 (Remarks for Author):

Quantitative multi-organ proteomics of fatal COVID-19 uncovers tissue-specific effects beyond inflammation

The authors have performed mass spectrometry (MS)-based analysis of post-mortem tissues from patients deceased from COVID-19 and compared with tissues from patients deceased from related lung infections as well as non-lung related diseases. COVID-19 specific tissue effects are described as well as systemic COVID-19 effects detected in the tissues.
The manuscript is well written and describes a unique COVID-19 cohort, analyzed using state of the art MS-based proteomics methods. Conceptually the deconvolution of systemic- and tissue-specific effects is interesting and could provide a useful approach in future studies.

Major comments

My major concern with this manuscript is that the experimental design is poorly described and needs to be updated both in the main text, the figure legends and the material and methods section before being accepted for publication.

-Detailed description of the control cases needs to be included in the manuscript. It is unclear how the control cases are selected, how many they are, if they are also postmortem samples, how the ethical approval was handled for these patients etc.

- The clinical metadata of the non-COVID patients used as controls should be described in a separate supplementary table.

-To increase the readability, the number of patients in each tissue- and disease/control group should be included in the main text in brackets (n=xx) as well as in figure legends, as the group sizes varies substantially in the different statistical comparisons.

I.e the numbers in figure 1a does not add up to 352 samples - the numbers in the figure needs to be explained in more detail. Figure 1b lacks information of number of cases and samples from each region/disease group, which should be added to make the figure useful for the reader. In figure 1a the lung controls are stated to be 26 and in the heatmap 3c there are 29 lung controls etc.

Minor comments

It would be interesting to see some more general descriptions on how well the FFPE postmortem proteomics data recapitulates the tissue proteome, by comparison with known tissue enriched proteins such as those described in the human protein atlas.

****
Pre-decision cross-commenting

Referee #1
After reading the other reviews, I am in complete agreement that the control samples are poorly described and could contribute significantly to misinterpretation of the COVID19-specific alterations.

Referee #2

I believe all of the referee's comments are reasonable, and also agree with the decision to invite a revision that addresses the comments. I would like to add that some comparison with the proteome differences between COVID-19 patients and controls found by Nie et al. (2021), who conducted a similar study, would be useful.

Referee #3
I agree with the comments raised by the other two reviewers. I also think that relating these results to those in Nie et al would be very useful. Overall I would support the decision to give the authors a chance to address the concerns.

May 19, 2023

Point-by-point response in the review process for 'Quantitative multi-organ proteomics of fatal COVID-19 uncovers tissue-specific effects beyond inflammation'.

We thank the reviewers for their thorough evaluation of our manuscript and highly appreciate their constructive comments from both a proteomic and medical point of view. All reviewers appreciated the robust technology enabling our clinical study on FFPE tissue, as an important comparison at the proteomics level compared to the many studies with other 'omics' technologies in the context of COVID-19.

While all reviewers were generally positive, there were two main requests in the cross-commenting section:

1) Additional details on the cohorts and on the study design: In the revision we now first focused on a thorough description of the control cohort across organs resulting in several new EV tables/datasets. In parallel, we edited the description of the experimental design to clarify the relation between cohort samples and patients.
2) Comment more on the commonalities and differences to the previous work on a similar topic by Nie et al.: We appreciate this point, which will also be on the minds of future readers of the paper. In the revised manuscript, we go to greater length to directly compare results from the two studies. Briefly, there are mutually confirmed findings, but also complementary ones in the proteomic profiling of COVID-19 across different organs. Generally, with the benefit of our analysis strategy, we believe that we are able to unmask the tissue specific effects of COVID-19 infection from the systemic, circulation mediated part. We hope that this has become clearer in the revision.

Furthermore, as requested, we now discuss the matter of batch correction and the normalization of proteomic and phospho-proteomic data. By adding a new and readily accessible EV dataset containing all proteomic data across organs, we improved the data availability of our study as suggested by the reviewing committee. Finally, we elaborate on the extent of tissue markers that were consistently detectable in the FFPE samples of our autopsy cohort and the remaining important comments and suggestions from the reviewers.

We revised these and other comments of the reviewers in detail and addressed all of them in the point-to-point response below. Changes in the text, figures and tables were highlighted in the manuscript (‘track changes in Word’). Due to changes in the manuscript outline, the numbering order of EV/appendix figures and tables as well as of panels in the main figures has changed. Note that all changes in the revised manuscript are also listed (*in green*) in this response to facilitate the reviewing process.

Referee #1

Referee #1 (Comments on Novelty/Model System for Author):

I have some questions about the data normalization (see full review).

Referee #1 (Remarks for Author):

Schweizer et al have performed proteomic analysis of hundreds of COVID-19-infected tissues from post-mortem autopsies across several major human organ systems. The authors have also conducted proteomic analysis of samples from other lung diseases (e.g., influenza, diffuse alveolar damage) and non-pathological controls. This work is of great importance given that proteomics has been applied much less frequently than transcriptomic approaches to COVID-19 due to the inherent technological challenges of proteomics. The work appears thorough and well done. Although the work is purely descriptive and can sometimes read like a "parts list", I agree with the authors that the value of this work is that it may "[give] rise to new hypotheses that can be validated or used in translational context by the community."

We thank the reviewer for the positive feedback, including regarding our work as ‘of great importance’ and the thoughtful comments on the process of data normalization. We hope to address all concerns thoroughly in this response. Below, we explain the matter of batch correction and supply a new figure on this (**Appendix Figure S1**). Moreover, we discuss normalization of the phospho-proteome and improve data availability of this manuscript through a new **Dataset EV1**. Finally, we comment on the evaluation of patient specific signatures in the context of this study design, elaborate on differences between virus variants and address diversity of COVID-19 patients in our study.

Major points:

(1) The authors have focused primarily on organ-specific signatures between COVID-19 and control samples. Have the authors considered patient-specific signatures, especially ones that might be conserved across organ systems?

We appreciate this interesting question regarding patient-specific signatures, especially due to the wide spectrum of clinical courses and manifestations that clearly suggest heterogeneity and potential individual proteomic signatures of COVID-19 patients. This is generally supported by the reviewer's comment below on cluster 6 in Figure 3C. However, the design of our study focused primarily on overlapping and distinct signatures in different organs to gain insights into the major COVID-19-associated patho-mechanisms at the proteome level, as described in the response to the reviewer's comment number (5) below. Given the limited number of patients, interindividual heterogeneity in patient characteristics and comorbidities and varying COVID-19 disease courses, we believe that patient-specific signatures may be too unstable or stochastic to robustly extract these individual profiles.
Furthermore, analyses and biological conclusions based on potential signatures in patient subgroups are limited as this study focuses exclusively on post-mortem autopsies in patients with fatal disease. We agree, however, that the analysis of patient-specific signatures with favorable and fatal disease outcomes on an individual basis in larger cohorts should certainly be a focus of future research.

(2) There is considerable variability in the COVID patients in Fig. 3C. For example, several COVID patients have Cluster 6 proteins upregulated even though this cluster is marked by "proteins that decreased in abundance compared to NPC controls." Can the authors analyze or speculate on the heterogeneity observed across patients?

We thank the reviewer for pointing out the variable patterns in Figure 3C that indeed could point out to patient-based variabilities and sub-group specific signatures in the lungs. However, we believe that potential subgroups within COVID-19 patients should be treated with caution, as detailed above. Because our cohort comprised heterogeneity in a limited number of patients including different comorbidities, a certain extent of variation between patients is expected. Given our study design, any disease-associated patterns identified despite this inter-patient variability should reflect stable COVID-19 signatures, despite patient-specific biases. In

principle, clinical factors such as the degree of fibrosis in each selected region, ventilation or age could contribute to the heterogeneity of proteomic signal observed in Figure 3C. However, we found that none of these parameters resulted in a distinct stratification of these patients compared to the COVID-19 group. Thus, although there surely are patient-specific signatures that may cause heterogeneity in the COVID-19 group, studying this is beyond the scope of this study due to the limited number of patients.

(3) It is unclear if any of the lung phospho-proteomes (Fig. 4) are from the same the samples as the lung proteomes (Fig. 3). If they are from some of the same samples, can any of the observed changes in phospho-sites be normalized to changes in protein expression level?

We thank the reviewer for pointing out the missing information on the study design for the phosho-proteomic data of the lungs. We apologize and now address this concern as follows. First, we inserted details on the sample selection and processing in the *Material and Methods* (page 34, line 26 – page 35, line2):

‘Regions representing the overall histology of each patient were annotated by a pathologist (T.S.) and patient material was collected using a scalpel in a defined area of 25 mm^2 for each patient and organ. This procedure resulted in a total of 305 samples that were prepared for MS-based proteomics. For phospho-proteomic analyses, FFPE tissue was collected likewise from the entire slide to match the COVID-19 cohort of the lungs. Aiming for a total peptide amount of 85 ug in each sample prior to phospho-peptide enrichment, we directly matched 28 samples to corresponding samples of the full proteome, while 17 samples were combined within groups resulting in a total of 36 samples for phospho-proteomic data acquisition.’

Second, we re-phrased a sentence in the main text of the manuscript to summarize the relationship between proteome and phospho-proteome (page 18, lines 27-29):
‘*Among those, 28 samples directly matched samples of the proteome measurements, whereas 17 samples reflected the proteome of multiple specimens within one group.’*

Third, we added an explanatory sentence in the legend of Figure 4C to clarify the relationship between the groups depicted in the annotation of this heatmap and the previous figures (page 21, lines 11-12):

'COVID-19 and control groups partially or entirely match the groups shown in the full proteome of the lungs (Figure 3).'

Regarding the normalization of phosphorylated sites to the proteome, a point also raised by reviewer #2, we are well aware to this important issue. Ideally, one would separately determine changes in the proteome and phospho-proteome and take their ratios. However, as we know from previous experience and as is also acknowledged in the literature (Li *et al*, 2019; Wu *et al*, 2011), this requires a very deep coverage of the proteome, which has so far only been reached in cell culture studies and certainly not in the FFPE tissue available here (**Response Figure 1 A, B** and new **Figure EV4 B**). We elaborated on this issue and the limitation on the data interpretation in the updated version of the manuscript (page 18, lines 34-37):

*'Of note, these parallel changes in the proteome may modulate to the outcome of the phospho-proteome. However, the degree of corresponding proteomic and phospho-proteomic information specifically derived from FFPE tissue can vary, thereby raising the necessity for a case-to-case consideration of proteomic abundance changes (**Figure EV4 B**).'*

To nevertheless address this issue to validate the biological findings, we originally created **Supplementary Figure 4 E and F** in the initial version of this manuscript. Following the reviewer's concerns, we have now rearranged the figures in the revised version of the manuscript: former **Supplementary Figure 4 F** has become main **Figure 4E**, while the elements A,B of **Response Figure 1** are now part of **Figure EV3** to visualize the low degree of corresponding proteomic and phospho-proteomic data, preventing global normalization of phosphorylation data. **Figure 4 E** now shows the fold changes between COVID-19 and controls in both datasets. These clearly show higher fold changes of the phospho-proteome for proteins of Rho GTPase signaling and mRNA splicing than observed in the proteome changes alone.

In addition, we decided to point out again in the 'Material and Methods' section that the phosphorylation data were processed using a FASTA file including protein isoforms to clarify the interpretation of our results (page 38, lines 13-14):

'Notably, these FASTA files also included protein isoforms which may be relevant for the interpretation of resulting phosphorylation sites'.

(4) Can the authors use the phospho-proteomic data (Fig. 4) to infer which kinases are most upregulated in COVID? For example, methods like Post-Translational Modification Set Enrichment Analysis (PMID: 30563849).

We appreciate this suggestion and applied the suggested site-specific PTM signature enrichment analysis (PTM-SEA) to our phosphorylation data (Krug *et al*, 2019). Upon filtering for significance (FDR <= 5%), we created enrichment patterns from two different databases, however, no distinct signature could be identified (**Response Figure 1 C**). Hence, it appears that the interpretation of phosphorylation data in this study may be limited to the identification of Rho signaling and mRNA splicing that we were able to interpret in relation to the proteome.

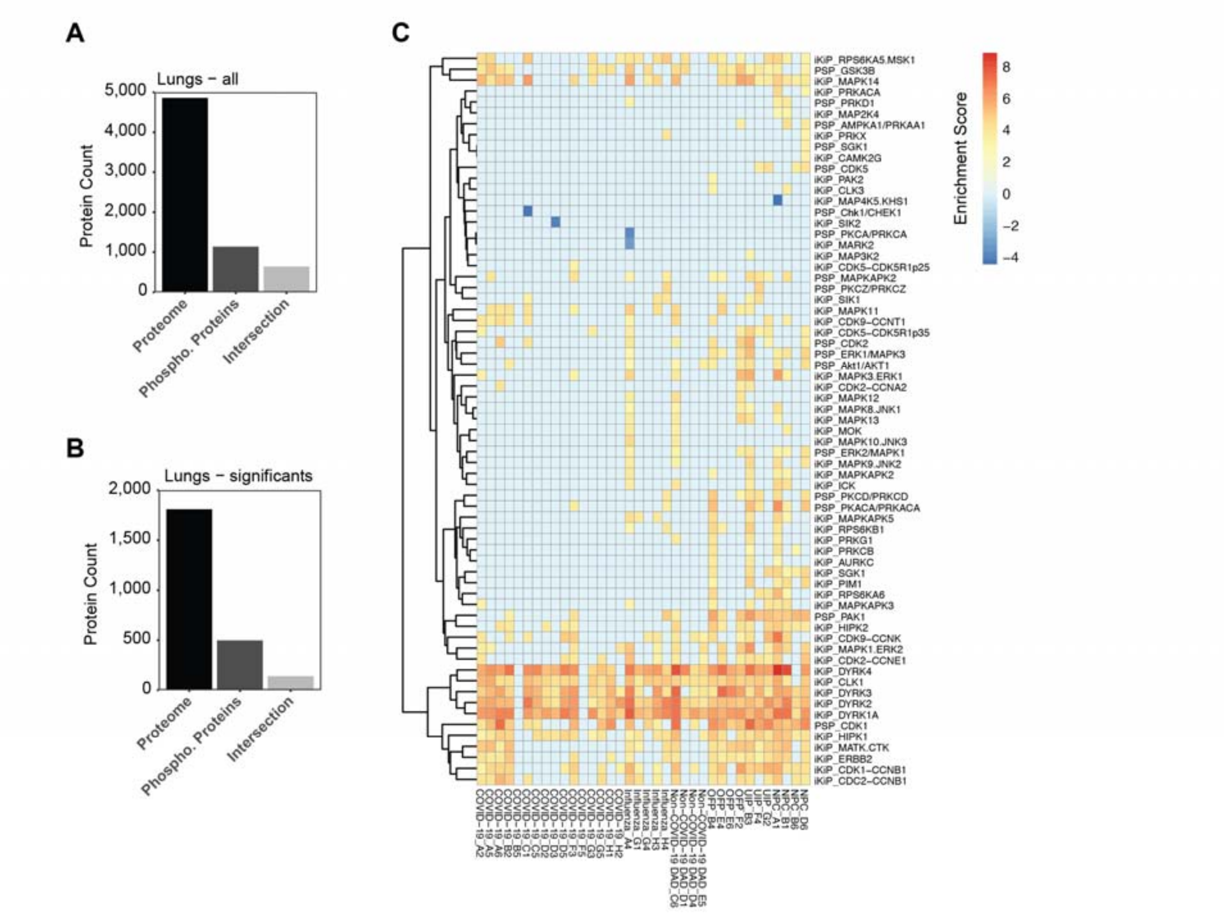

Response Figure 1 – Normalization and extended analysis on the phospho proteome

A, B Overlap between the full proteome and phospho proteome on the level of proteins (sites were summarized to proteins). The bar plots visualize the low degree of overlap for the entire dataset (**A**) and proteins of significant differential regulation (**B**), thereby preventing global normalization of phosphorylation abundances. Phosphorylation sites were filtered for confidence as described in the main manuscript.

C PTM-SEA analysis (Krug et al, 2019). The heatmap visualizes enrichment scores for entries from the associated database. Scores were filtered for significance (FDR <= 5%) while non-significant values were set to 0.

(5) With such a large sample cohort, did the authors need to do any batch correction of the data? They mention "computer-based randomization of samples ... was used to exclude batch effects during sample preparation." but this doesn't exclude batch effects as the samples were run on the LC-MS.

We apologize for not making this completely clear. We randomized the samples of every tissue type within one plate. Thus, there can be no plate associated batch effects for the different tissues (given that we do relative comparisons). LC-MS based batch effects should be minor in the first place, given our set up, and particularly here since individual plates were measured in at most four days. We clarify this as follows *(page 35, lines 5-8)*:

'FFPE tissue was collected into a 96-well plate […] keeping each tissue type within one plate. For each of these plates, COVID-19 and control samples of matching tissues were randomized and measured without interruption, avoiding batch effects.'

In more detail, there are common batch correction strategies based on linear regression and smoothing based on median quantities using MS and sample processing control samples (Messner *et al*, 2020). In addition, references across multiplexing channels (Mertins *et al*, 2018) have shown great benefit in previous work. However, we did not directly compare different organs by MS intensities, but conducted organ-centric relative analyses by comparing the results of Student's t-tests (fold change, p-value and their product, here termed 'ranking factor') for COVID-19 and control samples between organs. As pointed out in the corrected sentence on the sample collection above, we limited the collection of one tissue type to one plate, e. g. all COVID-19 and control samples of the lungs were collected and processed on plate 1 of the study.

However, aside from sample preparation, we acknowledge the reviewer's other concerns that all data were acquired in a specific order using the LC and MS instruments (drift rather than batch differences). To address this, we first analyzed the median protein intensity along the MS measurement order (**Response Figure 2**). We observed consistent levels of median protein intensities that can be related to the data quantification algorithm of the Spectronaut software, which compensates for possible drifts by a global normalization to the median sizes. We specified the normalization strategy in the revised version of the manuscript *(page 38, lines 9-10):*

‘A global normalization of data based on median quantities was enabled, eliminating possible MS intensity drift over time.’

To also make the information in Response Figure 2 accessible to the reader and to clarify our strategy in the main text, we have included it as a new **Appendix Figure S1** in the updated version of our manuscript. The numbering of all subsequent figures has been adjusted in the manuscript and the new figure has been referenced as follows *(page 5 , lines 7-9)*:

‘*Samples were processed for each tissue separately to allow proteomic stratification within each organ as well as an organ-wide relative assessment (Appendix Figure S1).’*

To further exclude a biological impact of the MS data acquisition order, we performed a Principal Component Analysis (**Response Figure 3**) for each organ to investigate the main driver of variance per cohort. Across all organs, the first two dimensions were driven by the biological grouping into COVID-19 and control specimen, rather than the sequence on the LC-MS instrumentation. This demonstrates that our relative multi-organ study did not require a batch correction, while correction of a potential intensity drift was already performed by the Spectronaut analysis software.

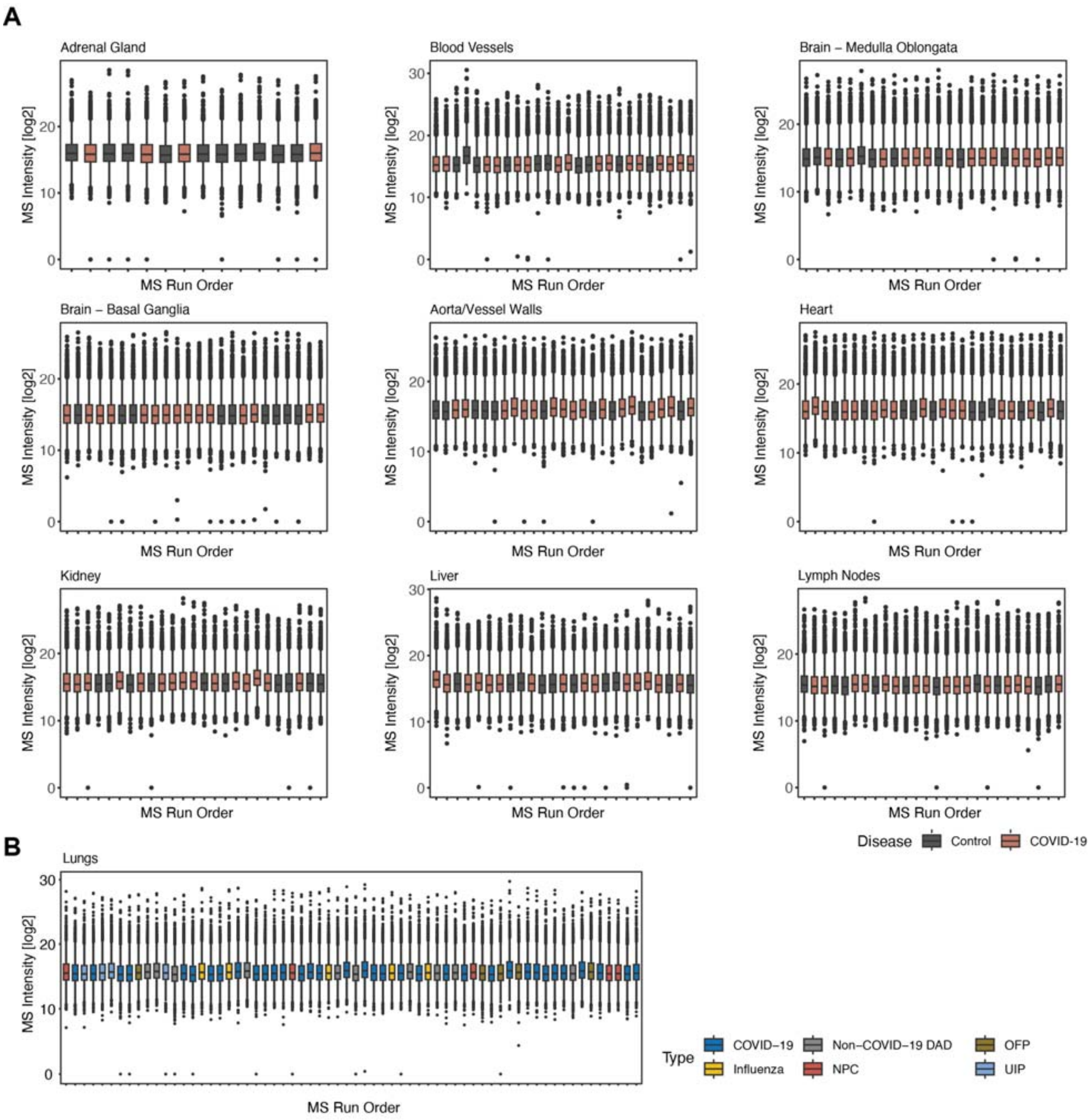

Response Figure 2 – Organ-wise evaluation of technical drift effects

A Log2 intensities plotted along the temporal axis of MS data acquisition for each organ. Samples of the COVID-19 and control group were randomized and colored accordingly. Boxes depict the inter quartile range (between 25th and 75th percentile) with the median intensity highlighted as bold lines, 95% of the data are contained within the lower and upper whiskers.

B Log2 intensities of the lungs as plotted in **A.** COVID-19 and diverse control groups were randomized and colored accordingly.

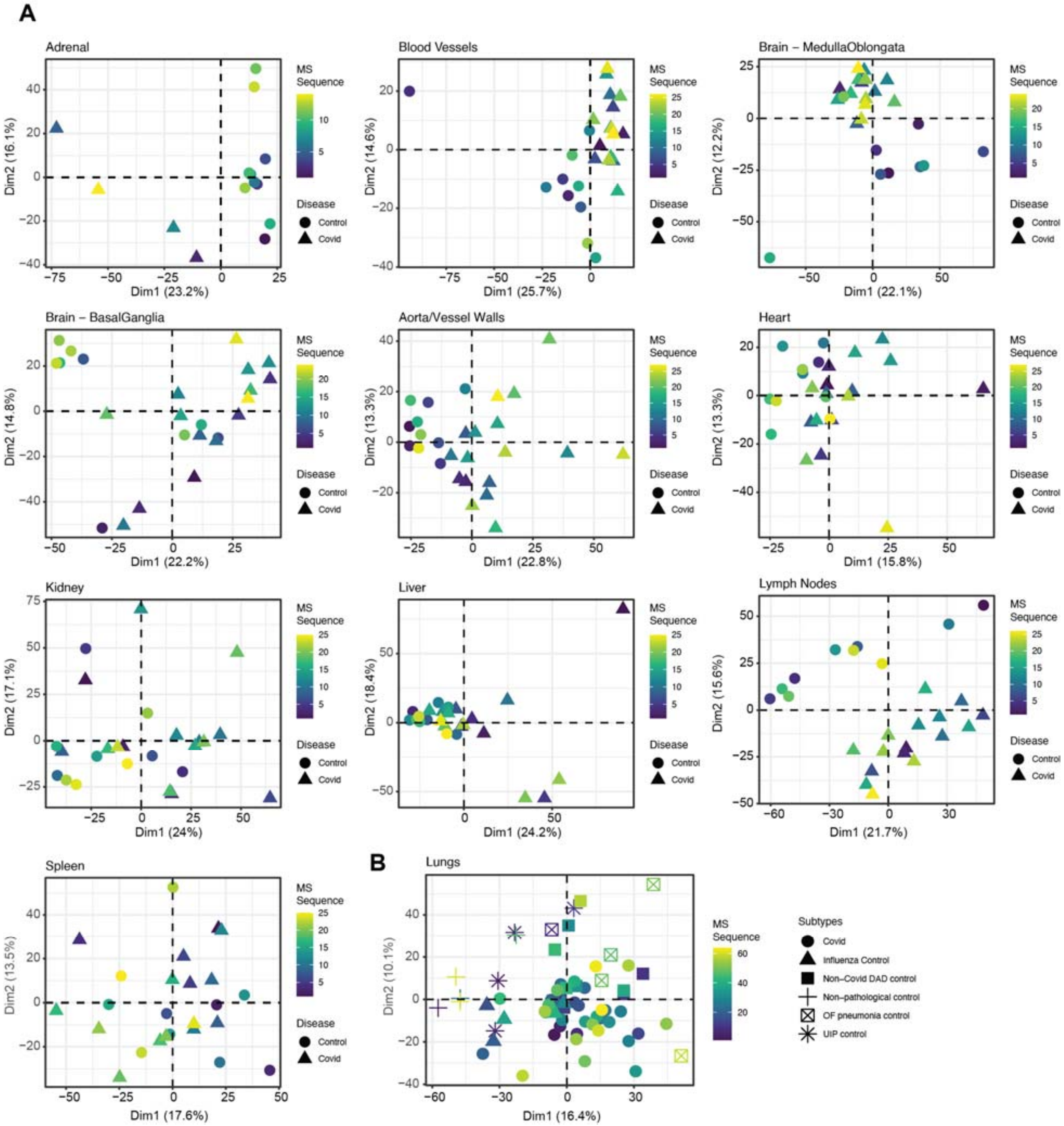

Response Figure 3 – No biological impact of the MS run order

A Principal component analysis (PCA) of all organs of this study. COVID-19 and control samples are indicated by point shapes, the MS measurement order sequence for each organ by color. Data were filtered for 80% valid values in each group and imputed from a left-centered Gaussian distribution (width: 0.3, downshift: 1.8).

B PCA of the lungs as shown in **A**. The extended control group of the lungs was annotated by shape type.

(6) I didn't find any details about normalization of the data in the Methods. Were the raw protein intensities simply log2 transformed? Did the authors investigate the effects of data normalization strategies across all the samples (i.e., simple methods like median subtraction or more complex methods like singular value decomposition such as EigenMS)?

The proteomic data were indeed log2-transformed as described in the section 'Bioinformatics data analysis': 'In preparation for the analysis, protein intensities were log2-transformed' (page 38, line 27). With regard to the details provided to the reviewer's comment (5), we conclude that the data do not require additional data normalization beyond the log2 transformation.

(7) page 26: the authors should clarify this statement: "In both regions we quantified more than 4,000 protein groups that showed equal levels in both areas of COVID-19 and control specimen (Fig 6B)." According to the legend, it's actually the "Number of identified protein groups" that were equal, not the levels (i.e., which for me levels = protein expression levels).

We thank the reviewer for picking this up and have rephrased as follows (page 26, lines 6-9):

'In both regions we quantified more than 4,000 protein groups that showed consistent identification numbers in both areas of COVID-19 and control specimens (Fig 6B, Table EV5).'

(8) The Supplemental Tables of the proteomic data do not include data from the individual samples. There is only summary data (e.g., log2 fold change of protein phosphorylation in the lungs). The data would be much more useful is the authors included the data from the individual samples as well.

Experimental data from individual samples were uploaded to the Mass Spectrometry Interactive Virtual Environment (MassIVE) of the ProteomeXchange consortium (Identifier MSV000090983) and will be available for public download subsequent to the revision process without restrictions. However, we agree that the direct availability to the data as supplementary table would be very useful to the reader. Hence, we summarized the quantification data of all organs in the new **Dataset EV1** and highlight this in the beginning of the 'Results' section (page 5, lines 9-11).:

*‘Altogether, this resulted in more than 350 human tissue proteomes from 19 COVID-19 and a heterogeneous group of 85 control patients to be analyzed in a robust, quantitative and reproducible manner (**Dataset EV1**).’*

(9) The authors have analyzed samples from the very early phases of the COVID-19 pandemic (April to May 2020). Can the authors speculate on how the emergence of new COVID-19 variants might change their results?

We welcome this interesting and thought-provoking commentary. Unfortunately, we do not have experimental data in this regard, thus, we are left to speculate here. We have inserted these potential consequences of the new variants of SARS-CoV-2 on the associated possible proteomic changes and the accompanying changes in the clinical presentation of COVID-19 in the discussion of the revised manuscript (page 32, lines 14-24):

‘In summary, the quantitative proteomics approach of this study showed the specific involvement of multiple organs in the host response to COVID-19 to different degree in systemic inflammation and beyond. Our investigation covers the early phases of the pandemic whereas the virus has mutated considerably since, impacting transmission rates and pathogenicity. For instance, the Delta variant (B.1.617.2) was highly transmissible and might have been associated with an increased risk for hospitalization. Likewise, pathogenicity differed between virus variants and host mortality was reduced by factors such as the improvement of the host immune response via vaccination or infection over the course of the pandemic (PMID: 36653446). These developments also suggest altered effects on the host tissue proteome and could be investigated by stratifying SARS-CoV-2 variants, disease progression, immunization status and patient demographic characteristics.’

<u>Minor points</u>:

(1) Typo, Fig 1 legend: "A total of 352 specimen from"

We thank the reviewer for pointing out this repeating typo. We have changed the text accordingly in a rephrased version of the previous sentence to *‘Specimens of ten different organs from 19 patients’* in the legend of Fig 1.

(2) Typo, page 10: "This revealed that markers of the RBC panel dominated some specimen of the COVID-19 cohort"

The text on page 10 has been corrected: *'This revealed that markers of the RBC panel dominated some specimens of the COVID-19 cohort […]'.*

(3) Typo, Fig. 5 legend: "Differential protein expression between COVID-19 control specimen in the lymph nodes"

The text in the legend of Fig 5 has been corrected: *'Differential protein expression between COVID-19 control specimens in the lymph nodes'.*

Referee #2

Referee #2 (Comments on Novelty/Model System for Author):

Novelty is reduced by publication of the paper by Nie et al. 2021. The model system is inadequate at this stage because there is insufficient information about the control subjects to evaluate claims that proteomic differences between COVID-19 patients and controls are indeed due to COVID rather than unrelated differences between patients and controls. Controls may be fine, but we just do not know.

These points are addressed in detail below, specifically we now supply the missing cohort information and a thorough comparison to the paper of Nie et al.

Referee #2 (Remarks for Author):

The manuscript by Schweizer et al. describes development (actually improvement of current protocols) of a streamlined LC-MS/MS based clinical proteomic method and subsequent study of 352 FFPE tissue samples from 10 different organs of 19 COVID-19 patients who died from the disease and 9-10 (most organs) or more (26 lung) control samples. The depth of coverage from FFPE samples given relatively rapid analysis times (about 90 mins/sample) is impressive, making interesting clinical studies such as this one possible. The depth of coverage enabled the discovery of tissue-specific differences between COVID-19 patients and controls that went beyond the predominant circulating inflammatory molecules. While this work represents an

impressive demonstration of cutting-edge clinical proteomics technology, unfortunately lack of information about controls limits the utility of clinical insights from the differences in protein amounts between samples. In addition, there are no follow-up experiments to test hypotheses generated by the data or to compensate for problems with the controls.

We thank the reviewer for the constructive feedback and discuss all concerns in this response and the edited version of the manuscript. First and foremost, we now provide the new **Dataset EV2 and Tables EV4 and EV5** containing detailed data of the patients of our control cohort across all organs. Based on this information, we further clarify the reviewer's questions regarding the biological implications of our results. Moreover, we present the comparison of this study to the work of Nie et al. and provide this information also to the reader with additional text (see below) and as new **Appendix Figure S2**. Finally, we appreciate the reviewer's comments on the normalization of the phospho-proteomic data and discuss these alongside the similar question of reviewer #1.

Specific Comments:

(1) Info about COVID-19 patients is given in Supplementary Tables 1 and 2 but I cannot find any information about control individuals. This is a crucial omission, as we have no information about how well these individuals were matched for age, gender, and other health parameters. How can we know which differences are due to COVID-19 and which differences are due to other factors such as age or comorbidities between groups?

We thank the reviewer for pointing out this important shortcoming that limited the biological interpretation of our results in the initial version of the manuscript. We have now undertaken major efforts to collect comprehensive clinical data for the entire control cohort and have provided this in the new **Dataset EV2**. As noted before, our cohort cannot be fully representative of the population due to the limited sample size. We have now summarized critical information in response to the reviewer's concerns in **Table EV4 and EV5** and provide this to the reader.

As the reviewer may appreciate from **Dataset EV2** and specifically for all non-COVID-19 samples in the lungs (**Table EV4**), basic patient characteristics are very similar between COVID-19 and controls. For lung, we cover a similar age range between both groups. The same is true for gender and smoking, also risk factors for adverse disease outcomes in COVID-19.

Cardiovascular and metabolic comorbidities were more common in the COVID-19 group than in the control group. Of note, however, the incidence of preexisting chronic respiratory disease and active malignancies (mostly including lung cancer in regions other than the samples studied) was again similar in both cohorts. One notable difference was the origin of the tissue samples. While our COVID-19 cohort came exclusively from autopsies, half of our control samples in the lungs represented biopsies from living donors, which is now clear from the new supplemental dataset.

In summary, we have thoroughly addressed the valid and important concerns of the reviewer. As presented in the new EV tables and datasets, we are convinced that the pre-existing health parameters should not cause systematic bias or erroneous conclusions in the presented analysis.

Were these tissue differences, especially in lung, pre-existing, thus making those individuals more susceptible to death by COVID-19? Specifically, could the COVID patients have DAD as a pre-condition that rendered them susceptible rather than developing DAD-like changes as a result of COVID infection? / (i.e. cause and effect difficult to determine).

As shown in the previous Supplementary Table 3 (now **Table EV2**), a total of 26% of the COVID-19 cohort had pre-existing lung disease. This is similar to the control cohort (38%, **Table EV4**). However, none of the pre-existing conditions in the lungs of COVID-19 patients was diffuse alveolar damage (DAD) or associated with DAD (noted in the revision – please see below). We cannot formally exclude that pre-existing lung conditions may have had an impact on COVID-19 associated changes in the lungs. However, given the heterogeneity of our cohort, our data suggest that the observed proteomic changes are substantially associated with COVID19 rather than pre-existing conditions.

Neuronal changes could also be an effect of age, but we cannot evaluate this possibility without information about controls.

We agree that myelin sheath degradation and associated inflammatory processes, in particular, may be closely related to brain aging and have provided this relationship in the "Discussion" of our original manuscript. To support the relationship to COVID-19 rather than age as the reason for our observation, we provide the reviewer and the reader with the new **Table EV5**. Baseline patient characteristics (age, sex) were very similar between cases and controls in the brain analyses, ruling out the possibility that the observed proteomic changes in the neuronal system

were exclusively due to older age and age differences. In addition, we emphasize that, as in the COVID-19 cohort, all control samples in the brain were exclusively from autopsies.

Lack of info about controls is especially important given the fact that COVID fatalities occur most frequently in older patients, especially those with underlying morbidities.

As mentioned above and shown in the new EV tables and datasets, our cohorts present a slight disbalance in comorbidities, but otherwise a good overall balance between COVID-19 and control groups. This indicates that differences in clinical parameters (including comorbidities) are not responsible for the observed proteomic differences.

Although, we are convinced that the minor differences of clinical parameters between COVD-19 and control patients do not considerably impact our analyses, major findings and the conclusions, we draw the reader's attention to this potential limitation. We have commented on the basic patient characteristics and the minor differences in comorbidities in the 'Discussion' (page 32, lines 26-29).

'Despite of a control cohort of largely balanced clinical characteristics, heterogeneity of clinical treatment strategies and minor differences in comorbidities could also have an effect on the clinical interpretation of data.'

Moreover, we highlighted the new supplementary tables in the revised version of our manuscript (page 5, lines 11-13):

'Patient characteristics including BMI, smoking status and comorbidities are listed in Tables EV2 and EV3 for COVID-19 patients, while detailed information on control cases is given in Dataset EV2.'

and (page 14, lines 5-7):

'Notably, the selection of control samples included clinical factors such as pre-existing chronic respiratory disease and smoking status into the study design (Dataset EV2 and Table EV4).'

and (page 26, lines 7-9):

'[…] areas of COVID-19 and control specimens that were largely balanced for age, sex and other clinical parameters (Fig 6B, Table EV5).'

Table EV3: Specific clinical characteristics of the Lungs – COVID-19 and controls

Variable	***COVID-19***		***Controls***	
	number	percentage/range	number	percentage/range
Total number of patients	19	100%	29	100%
Median age (range)	73	57-90	66	19-82
Sex (male/female)	14/5	74%/26%	19/10	66%/34%
Smoker (yes/no)	7/12	37%/63%	8/18	28%/62%
Biopsy/autopsy	0/19	0%/100%	16/13	55%/45%
Cardiovascular comorbidities				
atrial flutter/fibrillation	11	58%	4	14%
hypertension	13	68%	14	48%
coronary artery disease	5	26%	1	3%
cardiomyopathy	5	26%	3	10%
arteriosclerosis	9	47%	3	10%
Metabolic comorbidities				
diabetes	6	32%	4	14%
hyperlipidemia/ hypercholesterinemia	4	21%	5	17%
obesity [BMI]	9	47%	7	24%
BMI [kg/m^2]	28.3 kg/m^2	19.6-66.2	23.1 kg/m^2	14.7-33.0
Chronic respiratory disease	5	26%	11	38%
Chronic renal disease	7	37%	2	7%
Active malignancies	3	16%	6	21%

Table EV5: Specific clinical characteristics of the Brain – COVID-19 and controls

Variable	***COVID-19***		***Controls***	
	number	percentage/range	number	percentage/range
Total number of patients	19	100%	9	100%
Median age (range)	73	57-90	67	45-83
Sex (male/female)	14/5	74%/26%	6/3	67%/33%
Smoker (yes/no)	7/12	37%/63%	1/8	11%/89%
Biopsy/autopsy	0/19	0%/100%	0/9	0%/100%
Cardiovascular comorbidities				
atrial flutter/fibrillation	11	58%	2	22%
hypertension	13	68%	5	56%

coronary artery disease	5	26%	3	33%
cardiomyopathy	5	26%	2	22%
arteriosclerosis	9	47%	6	67%
Metabolic comorbidities				
diabetes	6	32%	2	22%
hyperlipidemia/ hypercholesterinemia	4	21%	0	0%
obesity [BMI]	9	47%	3	33%
BMI [kg/m^2]	28.3 kg/m^2	19.6-66.2	24.8 kg/m^2	18.0-36.4
Chronic respiratory disease	5	26%	1	11%
Chronic renal disease	7	37%	1	11%
Active malignancies	3	16%	5	56%

(2) The in-depth proteomic study by Nie et al. of 7 organs from 19 COVID-19 patients, cited in this manuscript, used a TMT approach and also reported non-inflammatory tissue differences and as a result reduces the clinical novelty of the manuscript by Schweizer et al.

We appreciate the quality and outcome of the study by Nie et al. which was published early in the pandemic. Nevertheless, given the global and continuing impact of COVID-19 we believe there is an urgent need for an independent proteomic study of broadly the same question. This is particularly the case because the required technology is quite advanced, explaining why there are so few studies of this kind. More fundamentally, the two studies differ in important ways in their design, data analysis strategies and conclusions that could be drawn from them.

Following the consensus of the reviewers in the 'pre-decision cross-commenting', we will compare the two datasets and then highlight novel aspects of our study. In the revised manuscript, we created a new **Appendix Figure S2** based on **Response Figure 4**.

Overall, the studies are complementary in that they deal with different organ groups, such as lymph nodes, adrenal blood vessels, aorta/vascular walls, and brain in our study compared to testis and thyroid tissue in Nie et al. (**Response Figure 4A**). They also sub-stratified tissues between white and red bulb of spleen as well as medulla and cortex of kidney. Together, Nie et al. investigated 7 different organs, whereas our study investigated overall 10 organs. In addition, we analyzed two brain regions, which is of great interest because neurological symptoms have been frequently described in COVID-19 and post-COVID-19 patients.

Most importantly, our separation of the systemic-inflammatory response is a key strategy distinguishing our manuscript from Nie et al. This allowed us to investigate COVID-19 directly in a tissue specific manner and beyond the shared systemic response mediated by the circulation. In the revised manuscript, we now compared the systemic-inflammatory effects of both datasets in the significantly altered proteome of the lungs as an exemplary organ (**Response Figure 4B**). This revealed that the proteomes of Nie et al. also contained many proteins related to systemic inflammation. However, these were not differentiated from the tissue specific effects as our strategy allowed us to do.

To systematically evaluate the outcome of both studies, we have now selected the lungs, liver and the heart as the most comparable tissue types by study design, while considering the systemic-inflammatory response in both datasets. Among these organs, the overlap of significant proteins (sp, t-test, q-val < 0.05, fold change >1.5) was most substantial in the lungs (sp = 188), followed by the liver (sp = 48), while the overlap was minimal in the heart (sp = 1) (**Response Figure 4C**). In the lungs, almost 1.5 times as many significant proteins (+43%) were identified uniquely in our proteomic investigation. For the liver and heart, unique identifications were higher in the study of Nie et al.

As the unique identifications were already discussed in the respective studies in detail, we next investigated commonly altered proteins in more detail.

The fold changes of significant proteins had a Pearson correlation of 0.78 in the lungs and 0.90 in the liver (**Response Figure 4B**). Among the proteins of highest correlation, the negative regulator of collagen production RCN3 and the Nicotinamide N-methyltransferase (NNMT) – both proteins prominently discussed in our manuscript - showed a comparable fold change between both studies. The acute-inflammatory protein CRP showed a higher fold change in our data, probably indicating higher levels of inflammation in our clinical cohort.

We have integrated a summary of these observations into the 'Results' of the revised version of the manuscript as follows:

(page 11, lines 16-19):

'This formed the basis for all subsequent analyses to determine organ-specific changes in SARS-CoV-2 infection after separating out the systemic inflammatory effects, something that had not been done in previous work <u>*(Nie et al, 2021)(Appendix Figure S2 A,B).*</u>

(page 14, lines 19-25):

'Reassuringly, proteins prominently discussed here such as RCN3 and NNMT showed comparable fold changes and directionality compared to previous work (Nie et al., 2021) (Appendix Figure S2 C,D). In contrast, we observed a somewhat greater increase of the acute-inflammatory protein CRP, perhaps indicating greater levels of inflammation in our clinical cohort. Conversely, we confirmed concordant upregulation in the lung of the transcription activators STAT1-3, cathepsins as well as the cytokine-related protein NAMPT, Thrombospondin-1 (THBS1) and CHI3L1, which modulates fibroblast proliferation (Dataset EV4).'

(page 23, lines 13-14):

'Proteins involved in liver metabolism such as Glutathione S-transferase A1 (GSTA1) were concordantly downregulated in our and previous data (Nie et al., 2021) (Appendix Figure S2 C,D).'

Moreover, we extended the comparison to Nie et. al. in the discussion section of our revised manuscript:

(page 30, lines 11-15):

'It directly associated the characteristic fibroblastic proliferation with increased levels of the fibroblast growth factor receptor substrate FRS3 and the negative regulator of collagen production RCN3, thereby expanding previous observations of fibroblast modulation in the lungs (Martinez-Martinez et al, 2017; Nie et al., 2021).'

(page 31, lines 29-31):

'Decreased functionality of the liver and its metabolic functions has been reported in severe COVID-19 (Mao et al, 2020 and Nie et al., 2021) and this is supported by our protein level data.'

(page 32, lines 31-34 – page 33, line 1):

'Besides recapitulating findings already reported in the literature, we here extend the number of previously investigated organs (Nie et al., 2021) and provide an in-depth molecular and systematic insight into the manifestation of COVID-19 beyond inflammation. Moreover, we characterize COVID-19 in the lungs as compared to other lung diseases of similar pathology.'

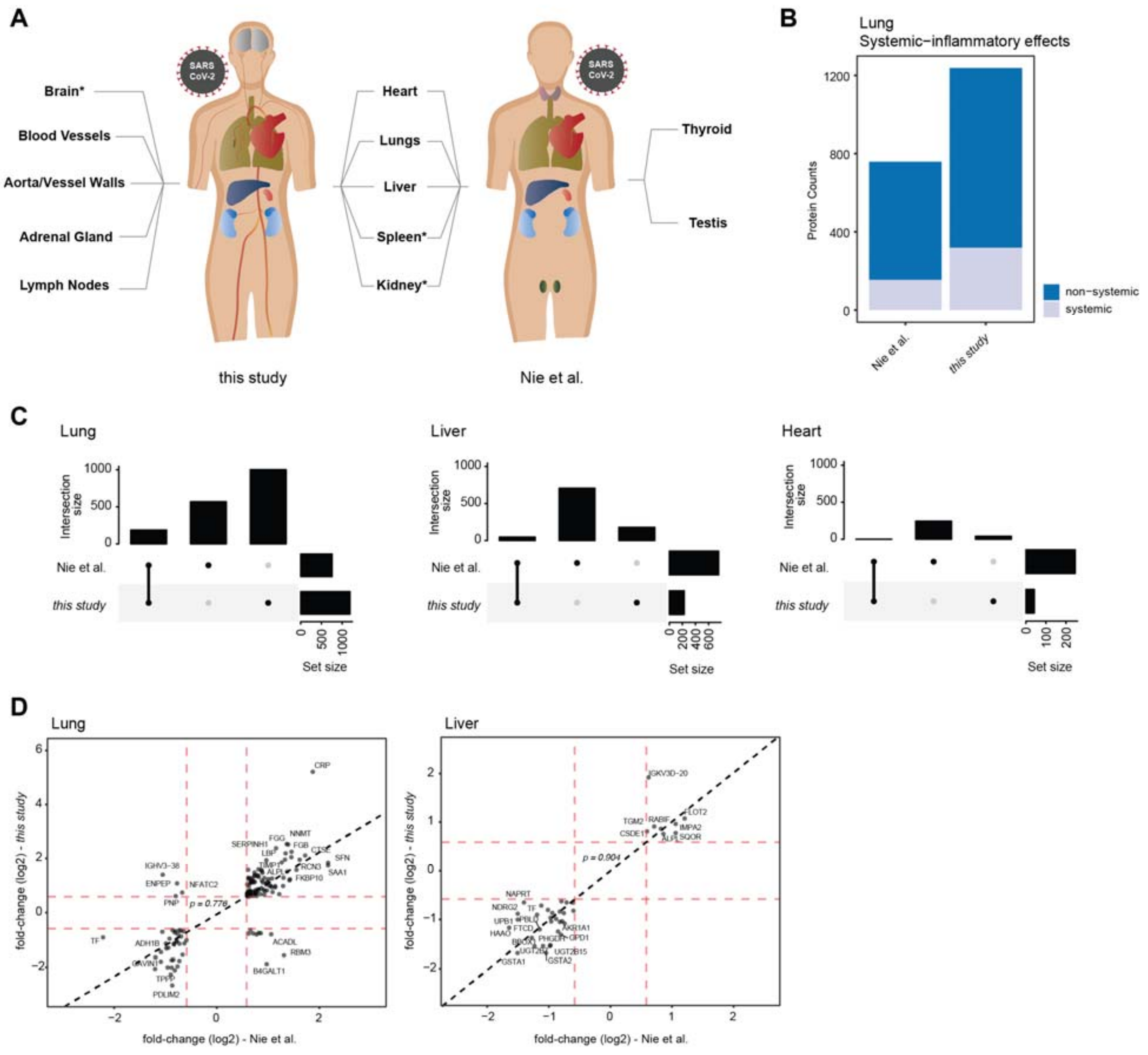

Response Figure 4 – Comparison to the study of Nie et al.

A Schematic comparison of investigated organs in a previous MS-based study of COVID-19 (Nie et al., 2021) and this work. Organs marked with an asterisk (*) were subset into physiological regions and were therefore excluded from the following comparison.

B Count of significantly differentially regulated proteins (t-test, adj.pval/q-va < 0.05, fold change >1.5) in both datasets stratified into systemic inflammatory and non-systemic proteins – a key strategy implemented in this study.

C Upset plots depicting the intersection of differentially expressed proteins compared between this study and previous work by Nie et al. for matching organs (the lungs, liver and heart).

D Comparison of fold changes for significantly differentially regulated proteins (t-test, adj.pval/q-val < 0.05, fold change >1.5, indicated by red dashed lines) between this study and Nie et al. Proteins in the lungs and liver scatter closely around the diagonal (dashed black line) indicating high correlation and common protein dysregulation.

(3) Filtering out circulation-mediated proteome changes helped to highlight organ-specific effects and is a good idea.

We appreciate the reviewer's positive feedback on our strategy which defines one of the novelties in this study. We hope that our manuscript demonstrates the utility of separating the circulation-mediated proteome in COVID-19 and beyond.

(4) It's not clear to me whether or not phosphorylation levels on individual sites were corrected for total amounts of the corresponding proteins in the phosphoproteomics experiments described in Fig 4. I don't believe so, but in that case protein levels rather than phosphorylation status of each protein would then be the key variable. For example, NPC, UIP and OFP which had the greatest phospoproteomic differences also had the largest protein differences (page 18).

Please see our answer to Reviewer #1, who asked the same questions (page 4-5).

<u>Minor Comments:</u>

(1) Throughout the manuscript supplementary figures are referred to as EV figures- this should be changed to make figure nomenclature consistent.

Thank you for catching this. We have now adapted our numbering to the journal guidelines. Five supplementary figures are now EV figures in the manuscript. The remaining three supplementary figures are summarized in the appendix and are referred to as 'Appendix Figure

S#' in the main text. Additionally, we adapted the guidelines for tables ('Table EV#') and datasets ('Dataset EV#').

(2) Supplemental Figure 1 A, B, and C: Presumably each bar represents a single sample/patient, this should be stated in the figure legend.

We thank the reviewer for pointing this out and have adapted the legend of **Figure EV1** accordingly:

(Figure EV1 A) *'[…] Bars represent tissue samples of different patients and were grouped by the employed LC system, respectively.'*

(Figure EV1 C) *'[…] Each bar depicts tissue samples of different patients while being grouped by the tested workflow, respectively.'*

(3) Page 19 line 2: should be "on average"

Changed in the revised version of the manuscript.

Referee #3

Referee #3 (Comments on Novelty/Model System for Author):

State of the art proteomics methods applied to a unique cohort, no direct medical applications, but of general scientific interest.

Referee #3 (Remarks for Author):

Quantitative multi-organ proteomics of fatal COVID-19 uncovers tissue-specific effects beyond inflammation

The authors have performed mass spectrometry (MS)-based analysis of post-mortem tissues from patients deceased from COVID-19 and compared with tissues from patients deceased from related lung infections as well as non-lung related diseases. COVID-19 specific tissue

effects are described as well as systemic COVID-19 effects detected in the tissues. The manuscript is well written and describes a unique COVID-19 cohort, analyzed using state of the art MS-based proteomics methods. Conceptually the deconvolution of systemic- and tissue-specific effects is interesting and could provide a useful approach in future studies.

We thank the Reviewer for the positive feedback and have addressed all comments as described below in detail. This includes a major revision on the experimental design that has been updated in several sections of the manuscript and the figures. Considering also the concerns of Reviewer #2, we thereafter address the selection, ethical approval and design of the control cohort. The latter is now summarized in the new **Dataset EV2** to provide a direct overview to the reader. Lastly, we elaborate on the extent of tissue markers that were consistently detectable in the FFPE samples of our autopsy cohort.

Major comments:

My major concern with this manuscript is that the experimental design is poorly described and needs to be updated both in the main text, the figure legends and the material and methods section before being accepted for publication.

We thank the reviewer for raising this important point which we have addressed by major updates of the manuscript text, figure legends and the material and methods section, as also described above in response to similar concerns of the other reviewers.

In the section 'Material and Methods', we have changed the title of 'Tissue samples' into 'Processing of tissue samples' and inserted details on sample collection. Consequently, redundant information was removed from the section 'Sample Preparation' (see also answer to Reviewer 31, page 4 of this response) (Manuscript page 34, line 26 – page 35, line2).

To clarify the selection of control samples in the main text, we re-arranged the first section of the 'Results' and added information on the selection and composition of all control groups (page 4, lines 25-31).

'A cohort of healthy control tissues was selected from non-COVID-19 patients from the FFPE sample archive of the Dept. of Pathology at University Hospital Augsburg for each organ, respectively. Tissue of the control cohort did not show a pathology in the selected organ, while

additional samples were collected for the lungs from phenotypes with similarities to COVID-19 such as influenza and non-COVID-19 related DAD. Overall, control samples originated from a heterogenous medical background but comparable age and gender distribution to the COVID-19 cohort.'

Finally, we have rephrased the legends of Figure 1A and 1B and included information on the experimental design:

(Figure 1A) *'Specimens of ten different organs from 19 patients with fatal COVID-19 (CVD) were analyzed in this study and complemented with an organ-matched control group (Ctrl) derived from non-COVID-19 patients. Control samples did not show a pathology in the respective organ, originated from a heterogenous medical background but comparable age and gender distribution in comparison to the COVID-19 cohort.*

(Figure 1B) '*For specimens of COVID-19 in the lungs, tissue was sampled from regions histologically classified into acute, organizing, and fibrotic stages of COVID-19 where possible. Additional to non-pathological control (tissue with healthy phenotype), these samples were compared to non-COVID-19 pathologies of the lungs including influenza, non-COVID-19 diffuse alveolar damage (DAD), usual interstitial pneumonia (UIP) with progressive fibrosis of the lung, and fibrosing organizing pneumonia (OFP).'*

(1) Detailed description of the control cases needs to be included in the manuscript. It is unclear how the control cases are selected, how many they are, if they are also postmortem samples, how the ethical approval was handled for these patients etc.

We thank the reviewer for the important queries regarding the organ samples of the different control cohorts. We first have complemented the ethical approval in the 'Material and Methods' section as follows.

Study design <u>and ethical approval</u> (page 34, lines 11-18).

'[…] This study was approved by the internal review board of the UKA (BKF No. 2020-18) and the ethics committee of the University of Munich (Project number 20-426, COVID-19 registry of the UKA). Ethical approval for the use of tissue samples as controls for the characterization of COVID-19 associated proteomic alterations is based on the study protocol of the COVID-19 registry of the University Hospital Augsburg (COKA). The study protocol of this registry specifically includes the possibility to include SARS-CoV-2 negative patients as a control group and was positively approved by the ethics committee of the Ludwig-Maximilians-University Munich under the project no. 20-426.'

Furthermore, we fully agree that clinical metadata of control patients/control samples are essential to assess the observed proteomic changes with respect to COVID-19 specificity. Therefore, we follow the advice of the reviewers and we have added a detailed description of the baseline clinical characteristics and conditions that existed at the time of sample collection to our analysis.

The revised text can be found in the answers to Reviewer #2 on pages 14-18.

(2) The clinical metadata of the non-COVID patients used as controls should be described in a separate supplementary table.

We agree that a separate supplementary table is needed to provide detailed clinical data of the patients of our control cohort across all organs. In the revised version of our manuscript, we have inserted the new **Dataset EV2** and **Tables EV4 and EV5**.

The revised text can be found in the answers to Reviewer #2 on pages 14-18.

(3) To increase the readability, the number of patients in each tissue- and disease/control group should be included in the main text in brackets (n=xx) as well as in figure legends, as the group sizes varies substantially in the different statistical comparisons.

I.e the numbers in figure 1a does not add up to 352 samples - the numbers in the figure needs to be explained in more detail. Figure 1b lacks information of number of cases and samples from each region/disease group, which should be added to make the figure useful for the reader. In figure 1a the lung controls are stated to be 26 and in the heatmap 3c there are 29 lung controls etc.

We appreciate the reviewer's suggestion and agree that listing the respective number of patients will improve the overview on the study cohort. To facilitate the evaluation by the reviewers and readers, the new **Table EV1** provides an additional overview on the study cohort and derived samples.

Table EV1: Overview on patients per tissue and derived samples. The table is complementing Figure 1A/B as well as the main text.

Tissue	Type	#Patients (#Samples from histological phenotypes)
Lungs	COVID-19	19 (35)
	Control - NPC	5
	Control - UIP	4
	Control - OFP	5
	Control – Non-COVID-19 DAD	6 (10)
	Control - Influenza	5
Adrenal Gland	COVID-19	4
	Control	10
Blood Vessel	COVID-19	16
	Control	10
Brain – Medulla Oblongata	COVID-19	15
	Control	9
Brain – Basal Ganglia	COVID-19	15
	Control	9
Heart	COVID-19	16
	Control	10
Kidney	COVID-19	14
	Control	10
Liver	COVID-19	15
	Control	10
Lymph Nodes	COVID-19	16
	Control	10
Spleen	COVID-19	15
	Control	10
Aorta/Vessel Walls	COVID-19	17
	Control	10
	Total #samples	305
	**Derived from**:	
	Total #COVID-19 patients	19
	Total #control patients	85 (compare to Augsburg)

Thereafter, we have implemented the reviewer's comment by adding the number of patients per tissue in the main text (page 4, lines 31-34 – page 5, lines 1-2):

*'The resulting cohort included patients from the COVID-19 and control groups (patient n = CVD/CTRL) from lung (n = 19/25), heart (n = 16/10), mediastinal lymph nodes (n = 16/10), blood vessels (n = 16/10), large vessel (aortal) walls (n = 17/10), brain (medulla oblongata, basal ganglia) (n = 15/9), liver (n = 15/10), spleen (n = 15/10), kidney (n = 14/10) and adrenal glands (n = 4/10) and selected them for proteomic analysis (**Fig 1A, Table EV1**).'*

The same patient numbers were also added to the figure legends (page 7, line 8 – page 8, lines 1-5):

'Overall, the cohort comprised patients from the COVID-19 (CVD) and control groups (CTRL) from lung (CVD = 19, CTRL = 25), heart (CVD = 16, CTRL = 10), mediastinal lymph nodes (CVD = 16, CTRL = 10), blood vessels (CVD = 16, CTRL = 10), large vessel (aortal) walls (CVD = 17, CTRL = 10), brain (medulla oblongata, basal ganglia) (CVD = 15, CTRL = 9), liver (CVD = 15, CTRL = 10), spleen (CVD = 15, CTRL = 10), kidney (CVD = 14, CTRL = 10) and adrenal glands (CVD = 4, CTRL = 10).'

Additionally, we have noted that the number of patients in the lung controls was 25 instead of 26. We apologize for this mistake and we have corrected it in Figure 1A.

Because samples were collected from multiple regions in some patients, the number in Figure 1A deviates from the specific patient numbers inserted above (indicated in **Table EV1**). Overall, 305 MS runs were recorded for the main cohort from 19 COVID-19 patients and a total of 85 control patients. In addition, MS data were acquired for a deep fractionated DDA library of the lung cohort to verify the identification of SARS-CoV-2 peptides – which resulted in a total of 48 samples. Including the revised numbers, the total number of full proteome samples was 353. Figure 1A has been updated accordingly by the overall number of samples as well as the patient numbers of COVID-19 and the heterogeneous group of controls.

We regret the unclear description of sample collection in relation to each patient and now explain this procedure in more detail in the main text. We also include a corresponding comment in the legend of Figure 1. We hope that this, together with the illustration of the

different pathological phenotypes in Figure 1B, clarifies our experimental process (page 5, lines 5-11).

'To account for different pathological phenotypes within one specimen, several tissue regions were collected for COVID-19 and non-COVID-19 DAD controls. […] Altogether, this resulted in more than 350 human tissue proteomes from 19 COVID-19 and a heterogeneous group of 85 control patients to be analyzed in a robust, quantitative and reproducible manner.'

(Legend figure 1A)

'A total of 353 samples was acquired from 19 COVID-19 patients and 85 control patients.'

Additionally, we have inserted the numbers of patients for all lung controls into the main text (page 5, lines 2-4):

'The additional control group of the lungs comprised different types of phenotypically similar non-COVID-19 lung diseases including influenza (n = 5), non-COVID-19 diffuse alveolar damage (DAD, n = 6), common interstitial pneumonia (UIP, n = 4) with progressive fibrosis of the lung, and fibrosing organizing pneumonia (OFP, n= 5) (Fig 1B).'

We also verified the number of lung controls in our cohort in comparison to the heatmap in Figure 3C. With the new supplemental Table 1, the reader will now be able to relate the number of samples collected per sample to the heat map in Figure 3 C (e.g., 35 columns (dark blue) of COVID-19 lung samples correspond to the number of samples collected in Table EV1). We thank the reviewer for this relevant comment that improved the understanding of the sample collection in our study.

Finally, to ensure that the relationship between the samples collected and the representation of the samples in our figures is correct, we compared Figures 2-6 with **Table EV1** and confirm that the numbers in all figures are identical.

Minor comments:

(1) It would be interesting to see some more general descriptions on how well the FFPE postmortem proteomics data recapitulates the tissue proteome, by comparison with known tissue enriched proteins such as those described in the human protein atlas.

We thank the reviewer for this interesting comment. To provide information on the distinct properties of post-mortem samples in context of the tissue-specific proteome, we have followed the reviewer's suggestion and mapped 3,106 'tissue enriched' proteins (downloaded from the Human Protein Atlas) onto the COVID-19 cohort of our study (**Response Figure 5)**. The tissue markers were identified across a broad abundance range in all organs and included proteins such as the Pulmonary surfactant-associated protein A2 (SFTPA2) in the lungs, the enzyme PNMT catalyzing the transmethylation of noradrenaline to adrenaline in the adrenal gland as well as the B-cell differentiation antigen CD72 in the lymph nodes (**Response Figure 5A)**. To also obtain information on the control cohorts, we have selected the organs with the most enriched markers, the brain and liver, and found similar frequency distributions between COVID-19 and control groups (**Response Figure 5B)**. Thus, we conclude that postmortem tissue reflects the tissue-specific proteome and is therefore suitable for studies such as those presented in this manuscript.

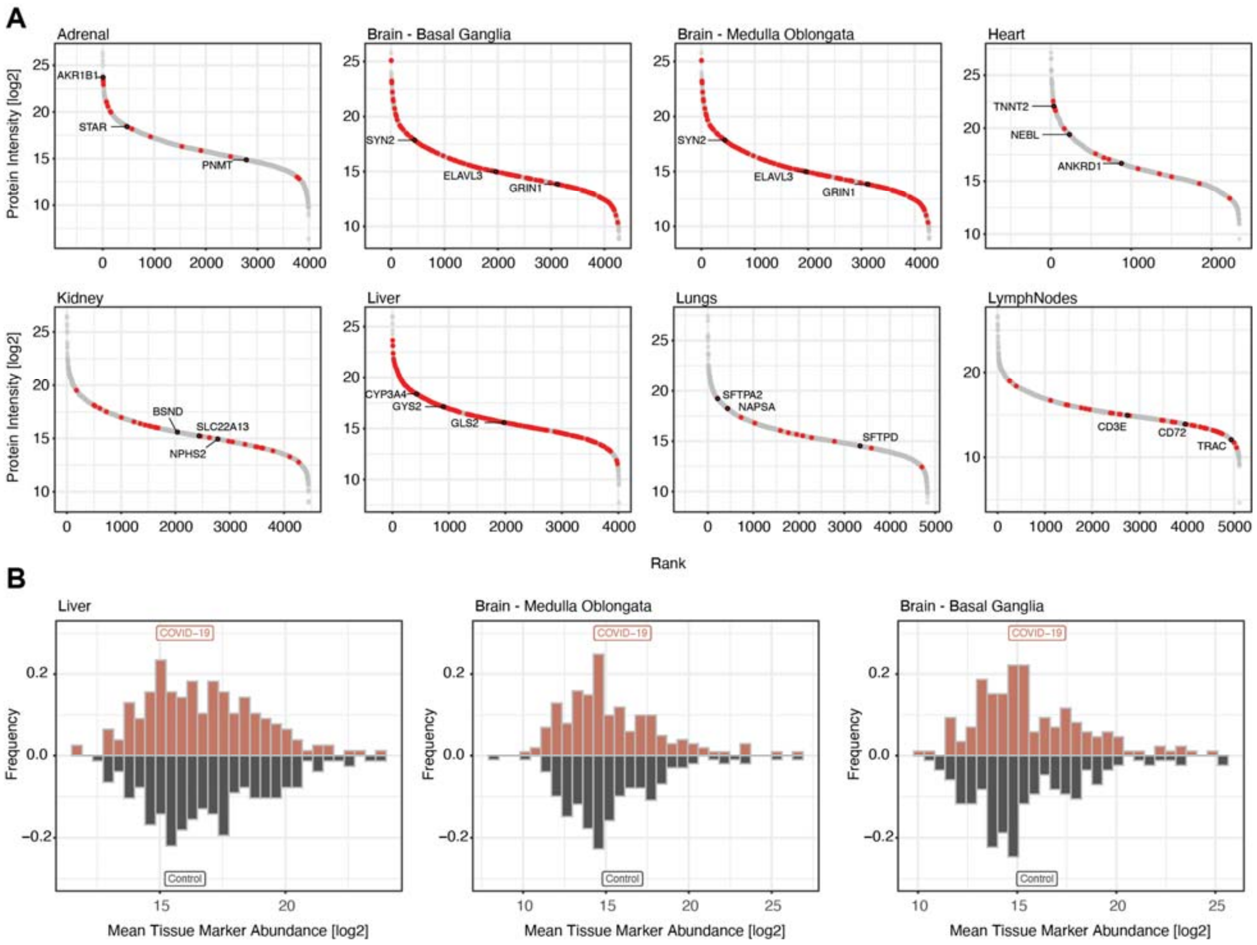

Response Figure 5 – Tissue enriched protein markers of the HPA in this study

A Indication of tissue enriched protein markers (derived from the Human Protein Atlas, HPA) among the COVID-19 proteome of each organ. All identified proteins within each organ were sorted by abundance (grey). Markers of the HPA are highlighted in red and are exemplary annotated in each plot.

B Histogram of tissue enriched proteins from the HPA in COVID-19 (red) and control samples (grey) for each organ, respectively.

Pre-decision cross-commenting

All these points are addressed in response to the individual reviewers.

Referee #1

After reading the other reviews, I am in complete agreement that the control samples are poorly described and could contribute significantly to misinterpretation of the COVID19-specific alterations.

Referee #2

I believe all of the referee's comments are reasonable, and also agree with the decision to invite a revision that addresses the comments. I would like to add that some comparison with the proteome differences between COVID-19 patients and controls found by Nie et al. (2021), who conducted a similar study, would be useful.

Referee #3

I agree with the comments raised by the other two reviewers. I also think that relating these results to those in Nie et al would be very useful. Overall I would support the decision to give the authors a chance to address the concerns.

References

Krug K, Mertins P, Zhang B, Hornbeck P, Raju R, Ahmad R, Szucs M, Mundt F, Forestier D, Jane-Valbuena J *et al* (2019) A Curated Resource for Phosphosite-specific Signature Analysis. *Mol Cell Proteomics* 18: 576-593

Li J, Paulo JA, Nusinow DP, Huttlin EL, Gygi SP (2019) Investigation of Proteomic and Phosphoproteomic Responses to Signaling Network Perturbations Reveals Functional Pathway Organizations in Yeast. *Cell Rep* 29: 2092-2104 e2094

Mertins P, Tang LC, Krug K, Clark DJ, Gritsenko MA, Chen L, Clauser KR, Clauss TR, Shah P, Gillette MA *et al* (2018) Reproducible workflow for multiplexed deep-scale proteome and phosphoproteome analysis of tumor tissues by liquid chromatography-mass spectrometry. *Nat Protoc* 13: 1632-1661

Messner CB, Demichev V, Wendisch D, Michalick L, White M, Freiwald A, Textoris-Taube K, Vernardis SI, Egger AS, Kreidl M *et al* (2020) Ultra-High-Throughput Clinical Proteomics Reveals Classifiers of COVID-19 Infection. *Cell Syst* 11: 11-24 e14

Nie X, Qian L, Sun R, Huang B, Dong X, Xiao Q, Zhang Q, Lu T, Yue L, Chen S *et al* (2021) Multi-organ proteomic landscape of COVID-19 autopsies. *Cell* 184: 775-791 e714

Wu R, Dephoure N, Haas W, Huttlin EL, Zhai B, Sowa ME, Gygi SP (2011) Correct interpretation of comprehensive phosphorylation dynamics requires normalization by protein expression changes. *Mol Cell Proteomics* 10: M111 009654

22nd Jun 2023

Dear Prof Mann,

Thank you for the submission of your revised manuscript to EMBO Molecular Medicine. I am pleased to inform you that we will be able to accept your manuscript pending the following final amendments:

1) Please address 2 points raised by the referee #2 by including additional EV tables (point 1) and reanalyzing the data from biopsies and autopsies as suggested by the referee (point 2).
2) In the main manuscript file, please do the following:
- Correct/answer the track changes suggested by our data editors by working from the attached document.
- Remove all figures.
- Please place main figure legends at the end of the manuscript followed by EV figure legends.
- Remove EV Dataset legends and EV Table legends and place them in the corresponding file in a separate tab.
- All figures and panels should be called out in a sequential order. Currently Fig. 5B is called out after Fig. 5C and D and Fig.EV4 B should go before Fig. EV4 C-F. Please correct. Also, please call out all individual panels for Appendix Figure S1. Supplementary Figure 5A is called out but doesn't exist.
- Please rename "Conflict of Interest" to "Disclosure Statement & Competing Interests". We updated our journal's competing interests policy in January 2022 and request authors to consider both actual and perceived competing interests. Please review the policy https://www.embopress.org/competing-interests and update your competing interests if necessary.
- Author contributions: Please remove it from the manuscript and specify author contributions in our submission system. CRediT has replaced the traditional author contributions section because it offers a systematic machine-readable author contributions format that allows for more effective research assessment. You are encouraged to use the free text boxes beneath each contributing author's name to add specific details on the author's contribution. More information is available in our guide to authors:
https://www.embopress.org/page/journal/17574684/authorguide#authorshipguidelines
3) Funding: Information about International Max Planck Research School for Life Sciences - IMPRS-LS is missing in our submission system. Please make sure that information about all sources of funding are complete in both our submission system and in the manuscript.
4) Tables: Please unzip all Dataset files and submit them as .xls files. Also, each EV Table should be uploaded as an individual .xls file.
5) The Paper Explained: Please add it to the main manuscript file.
6) Synopsis:
- Synopsis image: Please provide the synopsis image as a high-resolution jpeg file 550 px-wide x (250-400)-px high.
- Please check your synopsis text and image before submission with your revised manuscript. Please be aware that in the proof stage minor corrections only are allowed (e.g., typos).
7) For more information: This space should be used to list relevant web links for further consultation by our readers. Could you identify some relevant ones and provide such information as well? Some examples are patient associations, relevant databases, OMIM/proteins/genes links, author's websites, etc...
8) As part of the EMBO Publications transparent editorial process initiative (see our Editorial at http://embomolmed.embopress.org/content/2/9/329), EMBO Molecular Medicine will publish online a Review Process File (RPF) to accompany accepted manuscripts. This file will be published in conjunction with your paper and will include the anonymous referee reports, your point-by-point response and all pertinent correspondence relating to the manuscript. Let us know whether you agree with the publication of the RPF and as here, if you want to remove or not any figures from it prior to publication. Please note that the Authors checklist will be published at the end of the RPF.
9) Please provide a point-by-point letter INCLUDING my comments as well as the reviewer's reports and your detailed responses (as Word file).

I look forward to reading a new revised version of your manuscript as soon as possible.

Yours sincerely,

Zeljko Durdevic

Zeljko Durdevic
Editor
EMBO Molecular Medicine

*** Instructions to submit your revised manuscript ***

*** PLEASE NOTE *** As part of the EMBO Publications transparent editorial process initiative (see our Editorial at https://www.embopress.org/doi/pdf/10.1002/emmm.201000094), EMBO Molecular Medicine will publish online a Review Process File to accompany accepted manuscripts.

In the event of acceptance, this file will be published in conjunction with your paper and will include the anonymous referee reports, your point-by-point response and all pertinent correspondence relating to the manuscript. If you do NOT want this file to be published, please inform the editorial office at contact@embomolmed.org.

When submitting your revised manuscript, please include:

1) a .docx formatted version of the manuscript text (including Figure legends and tables)

2) Separate figure files*

3) supplemental information as Expanded View and/or Appendix. Please carefully check the authors guidelines for formatting Expanded view and Appendix figures and tables at
https://www.embopress.org/page/journal/17574684/authorguide#expandedview

4) a letter INCLUDING the reviewer's reports and your detailed responses to their comments (as Word file).

5) The paper explained: EMBO Molecular Medicine articles are accompanied by a summary of the articles to emphasize the major findings in the paper and their medical implications for the non-specialist reader. Please provide a draft summary of your article highlighting
- the medical issue you are addressing,
- the results obtained and
- their clinical impact.
This may be edited to ensure that readers understand the significance and context of the research.
Please refer to any of our published articles for an example.

6) For more information: There is space at the end of each article to list relevant web links for further consultation by our readers. Could you identify some relevant ones and provide such information as well? Some examples are patient associations, relevant databases, OMIM/proteins/genes links, author's websites, etc...

7) Author contributions: the contribution of every author must be detailed in a separate section.

8) EMBO Molecular Medicine now requires a complete author checklist (https://www.embopress.org/page/journal/17574684/authorguide) to be submitted with all revised manuscripts. Please use the checklist as guideline for the sort of information we need WITHIN the manuscript. The checklist should only be filled with page numbers were the information can be found. This is particularly important for animal reporting, antibody dilutions (missing) and exact values and n that should be indicted instead of a range.

9) Every published paper now includes a 'Synopsis' to further enhance discoverability. Synopses are displayed on the journal webpage and are freely accessible to all readers. They include a short stand first (maximum of 300 characters, including space) as well as 2-5 one sentence bullet points that summarise the paper. Please write the bullet points to summarise the key NEW findings. They should be designed to be complementary to the abstract - i.e. not repeat the same text. We encourage inclusion of key acronyms and quantitative information (maximum of 30 words / bullet point). Please use the passive voice. Please attach these in a separate file or send them by email, we will incorporate them accordingly.

You are also welcome to suggest a striking image or visual abstract to illustrate your article. If you do please provide a jpeg file 550 px-wide x 400-px high.

10) A Conflict of Interest statement should be provided in the main text

11) Please note that we now mandate that all corresponding authors list an ORCID digital identifier. This takes <90 seconds to complete. We encourage all authors to supply an ORCID identifier, which will be linked to their name for unambiguous name

identification.

Currently, our records indicate that the ORCID for your account is 0000-0003-1292-4799.

Please click the link below to modify this ORCID:
Link Not Available

12) The system will prompt you to fill in your funding and payment information. This will allow Wiley to send you a quote for the article processing charge (APC) in case of acceptance. This quote takes into account any reduction or fee waivers that you may be eligible for. Authors do not need to pay any fees before their manuscript is accepted and transferred to our publisher.

*Additional important information regarding Figures

Each figure should be given in a separate file and should have the following resolution:
Graphs 800-1,200 DPI
Photos 400-800 DPI
Colour (only CMYK) 300-400 DPI"

Figures are not edited by the production team. All lettering should be the same size and style; figure panels should be indicated by capital letters (A, B, C etc). Gridlines are not allowed except for log plots. Figures should be numbered in the order of their appearance in the text with Arabic numerals. Each Figure must have a separate legend and a caption is needed for each panel.

*Additional important information regarding figures and illustrations can be found at https://bit.ly/EMBOPressFigurePreparationGuideline. See also figure legend preparation guidelines: https://www.embopress.org/page/journal/17574684/authorguide#figureformat

The system will prompt you to fill in your funding and payment information. This will allow Wiley to send you a quote for the article processing charge (APC) in case of acceptance. This quote takes into account any reduction or fee waivers that you may be eligible for. Authors do not need to pay any fees before their manuscript is accepted and transferred to our publisher.

***** Reviewer's comments *****

Referee #1 (Remarks for Author):

The authors have satisfied all of my previous concerns.

Referee #2 (Comments on Novelty/Model System for Author):

The technical quality of the proteomic analyses is high, and there are some novel aspects to the workflow including separation of systemic from organ-specific effects. However, medical impact is low to medium because of some potential problems due to post-mortem differences between control and disease subjects. This issue is discussed in remarks to the authors below.

Referee #2 (Remarks for Author):

The authors clearly made a serious effort to respond to the reviewer comments and as a result the revised manuscript is much improved. In addition to addressing a number of technical concerns, addition of information about control subjects and a detailed comparison to the recent paper by Nie et al. were especially important and valuable. However, inspection of data about controls raises two issues that may be addressed by the authors:

1. Addition of information about control subjects in Dataset EV2 and Tables EV4 and EV5 is absolutely crucial for evaluating the significance of the biological conclusions. While the information in Tables EV4 and EV5 is easy to read and informative, the information in Dataset EV2 is much harder to understand quickly and requires the reader to calculate summary statistics and make comparisons to patient data found elsewhere in the manuscript such as Table EV 2. I strongly recommend production of additional EV Tables similar to EV4 and EV5 to summarize data for the other organs in addition to lung (EV4) and brain (EV5) to make the information more accessible and informative for the readers.

2. All of the samples derived from Covid patients were obtained at autopsy, and this was the case for control samples from some organs including brain and heart. However, as noted by the authors in their response to the initial submission, some of the control samples were obtained from biopsies of living subjects, including some of the samples from lung, kidney, liver, and spleen and all of the samples from adrenal glands, lymph nodes, vessel walls and vessels. It is well known that postmortem

interval can have a significant effect on proteomes, especially the phosphoproteome, and it is not hard to imagine that death, even after a short postmortem interval (we do not know the postmortem intervals) might affect the proteome at least as much as covid infection. As a result the authors need to perform some additional comparisons between control samples obtained by autopsy versus biopsy from at least one organ such as lung to measure the effects of death on the proteomic changes. It will be critical to know the extent of postmortem effects to interpret changes between control and patient samples. If the effect is significant, it may then be useful to remove biopsied control samples from analysis of organs whose control samples were obtained by both biopsy and autopsy, and to discuss possible contributions of this difference for organs whose control samples were obtained exclusively by biopsy. If there are no detectable differences between biopsied and autopsied control samples, then this should be reported- in either case this issue needs to be clarified for proper interpretation of the conclusions about the effects of covid-19 infection on the proteome.

July 11, 2023

Point-by-point response to the 2nd review of 'Quantitative multi-organ proteomics of fatal COVID-19 uncovers tissue-specific effects beyond inflammation'.

We thank the editor and the reviewers for conditionally accepting our manuscript, and are pleased that they believe that we have adequately addressed their previous concerns. Below, we address all remaining comments of Reviewer 2. This includes the generation of new Supplementary Tables EV6-13 to facilitate the readout of information given in Dataset EV2 regarding the control cohorts of our study. In addition, we fulfilled the reviewer's request to evaluate the post-mortem effect on the proteome by comparing control samples originating from autopsy and biopsy. This showed that there are no significant effects on the proteome and these results are now available as quality control (**Appendix Figure S4**).
Moreover, we addressed all of the editor's comments and reformatted the manuscript accordingly.

Reviewer comments

Referee #1

The authors have satisfied all of my previous concerns.

We are pleased to hear that our efforts sufficiently addressed the reviewer's comments.

Referee #2

Referee #2 (Comments on Novelty/Model System for Author):

The technical quality of the proteomic analyses is high, and there are some novel aspects to the workflow including separation of systemic from organ-specific effects. However, medical impact is low to medium because of some potential problems due to post-mortem differences between control and disease subjects. This issue is discussed in remarks to the authors below.

Referee #2 (Remarks for Author):

The authors clearly made a serious effort to respond to the reviewer comments and as a result the revised manuscript is much improved. In addition to addressing a number of technical concerns, addition of information about control subjects and a detailed comparison to the recent paper by Nie et al. were especially important and valuable.

We thank the Reviewer for the positive feedback on our efforts to improve the manuscript.

However, inspection of data about controls raises two issues that may be addressed by the authors:

1. Addition of information about control subjects in Dataset EV2 and Tables EV4 and EV5 is absolutely crucial for evaluating the significance of the biological conclusions. While the information in Tables EV4 and EV5 is easy to read and informative, the information in Dataset EV2 is much harder to understand quickly and requires the reader to calculate summary statistics and make comparisons to patient data found elsewhere in the manuscript such as Table EV 2. I strongly recommend production of additional EV Tables similar to EV4 and EV5 to summarize data for the other organs in addition to lung (EV4) and brain (EV5) to make the information more accessible and informative for the readers.

We agree and all information on the control cohort is now summarized and easily accessible as new Supplementary Tables EV6-13 complementing Dataset EV2.

2. All of the samples derived from Covid patients were obtained at autopsy, and this was the case for control samples from some organs including brain and heart. However, as noted by the authors in their response to the initial submission, some of the control samples were obtained from biopsies of living subjects, including some of the samples from lung, kidney, liver, and spleen and all of the samples from adrenal glands, lymph nodes, vessel walls and vessels. It is well known that postmortem interval can have a significant effect on proteomes, especially the phosphoproteome, and it is not hard to imagine that death, even after a short postmortem interval (we do not know the postmortem intervals) might affect the proteome at least as much as covid infection. As a result the authors need to perform some additional comparisons between control samples obtained by autopsy versus biopsy from at least one organ such as lung to measure the effects of death on the proteomic changes. It will be critical to know the extent of postmortem effects to interpret changes between control and patient samples.

If the effect is significant, it may then be useful to remove biopsied control samples from analysis of organs whose control samples were obtained by both biopsy and autopsy, and to discuss possible contributions of this difference for organs whose control samples were obtained exclusively by biopsy. If there are no detectable differences between biopsied and autopsied control samples, then this should be reported- in either case this issue needs to be clarified for proper interpretation of the conclusions about the effects of covid-19 infection on the proteome.

We thank the reviewer for prompting us to perform an additional control to evaluate if the post-mortem interval and sample processing affected the proteome in our study. To address this, we followed the reviewer's suggestion and compared biopsies and autopsies of the control cohorts of the kidney, non-COVID-19 DAD in the lungs as well as the spleen as these organs provided sufficient numbers of samples for a statistic comparison. Pairing biopsies with the same number of pathologically matching autopsies from the respective group (n=3 vs. 3 in each group), we performed a Student's T-Test (**Response Figure 1 A-C**). As can be seen in the volcano plots below, none of the proteins with a fold-change passed the significance level used throughout the manuscript (t-test, q-val < 0.05, fold change >1.5). Furthermore, the proteins with the highest significance do not follow an obvious biochemical theme (**Response Table 1**).
To further evaluate this, we correlated the fold changes of the comparison autopsies vs. biopsies with the result on the alterations related to COVID-19 as presented in the manuscript (see Dataset EV4 and EV6) for the previous organs. Notably, the distribution of data points in all tested organs did not correlate with alterations associated to COVID-19 (**Response Figure 1 D-F**). Hence, we are confident that the proteomics alterations reported in this manuscript are associated with the disease course of COVID-19 rather than the difference between post-mortem samples and autopsies. As we agree with the reviewer that this is an important point, we provide a new **Appendix Figure S4** (same as **Response Figure 1 D-F**) in the revised manuscript and add the following text to the 'Results' section:

(page 15, lines 25-27):

*Of note, there was no significant association of proteomic changes across organs with the post-mortem interval of patients (**Appendix Figure S4 A-C**).*

Regarding the cohort of Nie et al, all their comparisons were made between autopsies of COVID patients and biopsies of controls: *'Control samples in our study were selected from patients who had undergone surgery in the Union Hospital, Tongji Medical College, Huazhong University of Science and Technology from November 2019 to March 2020. All control samples are histologically healthy tissue samples from the non-COVID-19*

patients who were mostly victims of injuries. In case of lack of injured patients, benign lesions from cancer patients were procured' (Nie et al., 2023).'
However, by extension of our results shown above, we do not believe that this materially impacted their results and conclusions.

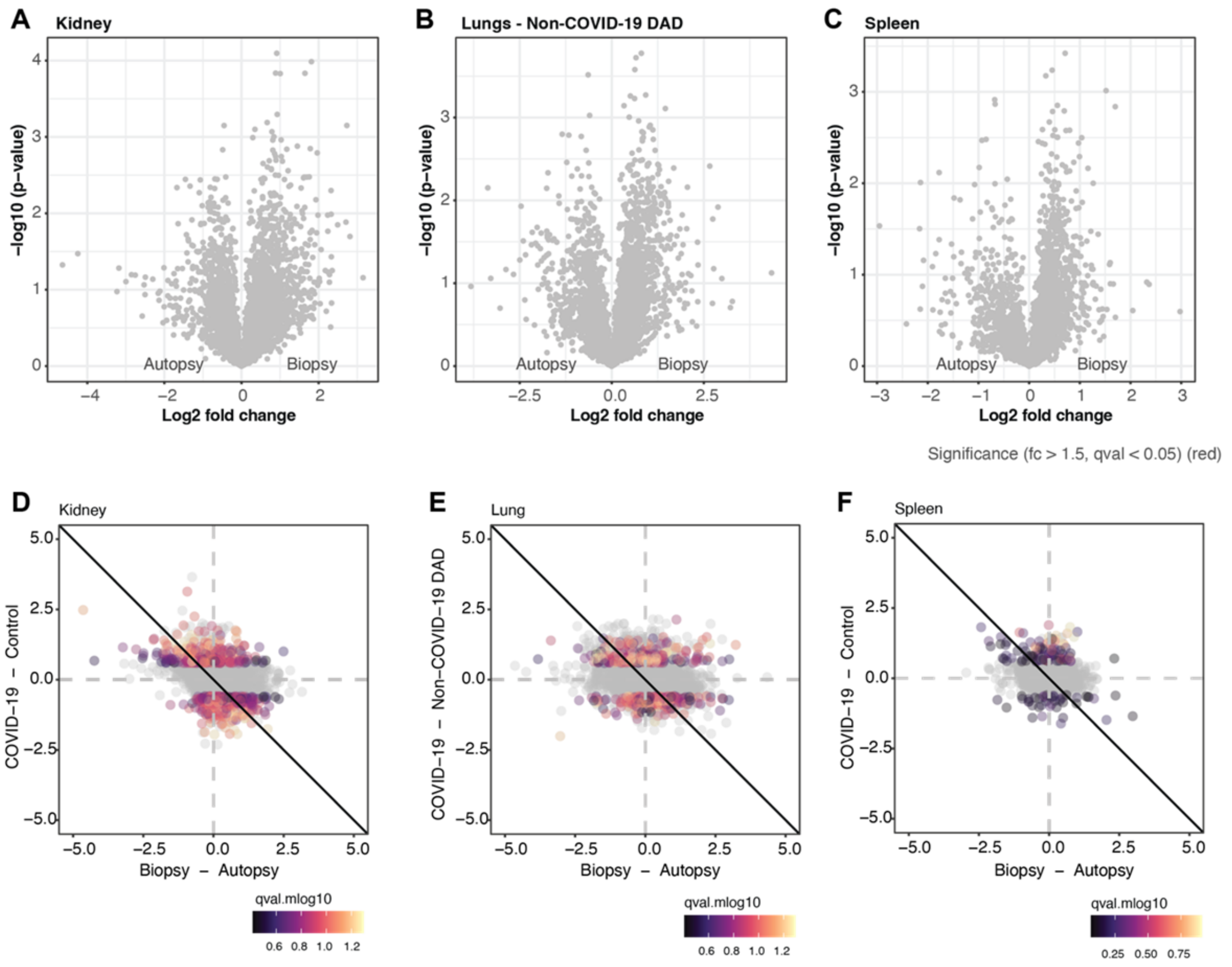

Response Figure 1 – Comparison of autopsy and biopsy-derived samples of the control cohort

A-C Differential protein regulation between three biopsy and autopsy samples in the kidney (**A**), the lung control cohort of non-COVID-19 related DAD (**B**), and spleen (**C**). Of note, no significant proteins (t-test, q-val < 0.05, fold change >1.5) were detected (Response Table 1).

D-F Evaluation of fold change correlation comparing samples derived from biopsy/autopsy with COVID-19 and control samples (n=3, respectively) for kidney (**D**), non-COVID-19 DAD in the lungs (**E**) and spleen (**F**). Diagonal lines illustrate the directionality that would be expected if there was a correlation between alterations associated with COVID-19 and changes introduced by

combining biopsies and autopsies. Notably, the distribution of data points in all tested organs did not correlate.

Response Table 1 – Summary of most significant changes assessed by p-value in the exemplary comparison of autopsy and biopsy-derived samples of the control cohort

Organ	*Gene symbol*	*Protein name*	*q-value*	*p-value*	*Log 2 Fold change*	*Rank*
Kidney	PGRMC1	Membrane-associated progesterone receptor component 1	0,08	8,01E-05	0,91	1
Lung[a]	IRF2BPL	Probable E3 ubiquitin-protein ligase IRF2BPL	0,21	1,68E-04	0,80	1
Spleen	PAICS	Bifunctional synthetase PAICS	0,47	3,78E-04	0,71	1
Kidney	MRPL14	Large ribosomal subunit protein uL14m	0,08	1,03E-04	1,81	2
Lung[a]	MYH9	Myosin-9	0,21	1,90E-04	0,66	2
Spleen	TCP1	T-complex protein 1 subunit alpha	0,47	5,79E-04	0,45	2
Kidney	EIF3G	Eukaryotic translation initiation factor 3 subunit G	0,08	1,46E-04	0,88	3
Lung[a]	PLXNB2	Plexin-B2	0,21	2,64E-04	0,63	3
Spleen	DUSP23	Dual specificity protein phosphatase 23	0,47	6,68E-04	0,33	3

[a] Analysis in the lungs was performed using the non-COVID-19 DAD control cohort

14th Jul 2023

Dear Dr. Mann,

We are pleased to inform you that your manuscript is accepted for publication and is now being sent to our publisher to be included in the next available issue of EMBO Molecular Medicine.

Please read below for additional IMPORTANT information regarding your article, its publication and the production process.

Congratulations on your interesting work,

Zeljko Durdevic

Zeljko Durdevic
Editor
EMBO Molecular Medicine

Follow us on Twitter @EmboMolMed
Sign up for eTOCs at embopress.org/alertsfeeds

*** *** *** IMPORTANT INFORMATION *** *** ***

SPEED OF PUBLICATION
The journal aims for rapid publication of papers, using using the advance online publication "Early View" to expedite the process: A properly copy-edited and formatted version will be published as "Early View" after the proofs have been corrected. Please help the Editors and publisher avoid delays by providing e-mail address(es), telephone and fax numbers at which author(s) can be contacted.

Should you be planning a Press Release on your article, please get in contact with embomolmed@wiley.com as early as possible, in order to coordinate publication and release dates.

LICENSE AND PAYMENT:

All articles published in EMBO Molecular Medicine are fully open access: immediately and freely available to read, download and share.

EMBO Molecular Medicine charges an article processing charge (APC) to cover the publication costs. You, as the corresponding author for this manuscript, should have already received a quote with the article processing fee separately. Please let us know in case this quote has not been received.

Once your article is at Wiley for editorial production you will receive an email from Wiley's Author Services system, which will ask you to log in and will present you with the publication license form for completion. Within the same system the publication fee can be paid by credit card, an invoice, pro forma invoice or purchase order can be requested.

Payment of the publication charge and the signed Open Access Agreement form must be received before the article can be published online.

PROOFS

You will receive the proofs by e-mail approximately 2 weeks after all relevant files have been sent o our Production Office. Please return them within 48 hours and if there should be any problems, please contact the production office at embopressproduction@wiley.com.

Please inform us if there is likely to be any difficulty in reaching you at the above address at that time. Failure to meet our

deadlines may result in a delay of publication.

All further communications concerning your paper proofs should quote reference number EMM-2023-17459-V3 and be directed to the production office at embopressproduction@wiley.com.

Thank you,

Zeljko Durdevic
Editor
EMBO Molecular Medicine

EMBO Press Author Checklist

Corresponding Author Name: Rainer Claus and Matthias Mann	
Journal Submitted to: EMBO Molecular Medicine	
Manuscript Number: EMM-2023-17459	

USEFUL LINKS FOR COMPLETING THIS FORM
The EMBO Journal - Author Guidelines
EMBO Reports - Author Guidelines
Molecular Systems Biology - Author Guidelines
EMBO Molecular Medicine - Author Guidelines

Reporting Checklist for Life Science Articles (updated January
This checklist is adapted from Materials Design Analysis Reporting (MDAR) Checklist for Authors. MDAR establishes a minimum set of requirements in transparent reporting in the life sciences (see Statement of Task: 10.31222/osf.io/9sm4x). Please follow the journal's guidelines in preparing your
Please note that a copy of this checklist will be published alongside your article.

Abridged guidelines for figures
1. Data
The data shown in figures should satisfy the following conditions:
- the data were obtained and processed according to the field's best practice and are presented to reflect the results of the experiments in an accurate and unbiased manner.
- ideally, figure panels should include only measurements that are directly comparable to each other and obtained with the same assay.
- plots include clearly labeled error bars for independent experiments and sample sizes. Unless justified, error bars should not be shown for technical
- if n<5, the individual data points from each experiment should be plotted. Any statistical test employed should be justified.
- Source Data should be included to report the data underlying figures according to the guidelines set out in the authorship guidelines on Data

2. Captions
Each figure caption should contain the following information, for each panel where they are relevant:
- a specification of the experimental system investigated (eg cell line, species name).
- the assay(s) and method(s) used to carry out the reported observations and measurements.
- an explicit mention of the biological and chemical entity(ies) that are being measured.
- an explicit mention of the biological and chemical entity(ies) that are altered/varied/perturbed in a controlled manner.
- the exact sample size (n) for each experimental group/condition, given as a number, not a range;
- a description of the sample collection allowing the reader to understand whether the samples represent technical or biological replicates (including how many animals, litters, cultures, etc.).
- a statement of how many times the experiment shown was independently replicated in the laboratory.
- definitions of statistical methods and measures:
  - common tests, such as t-test (please specify whether paired vs. unpaired), simple χ2 tests, Wilcoxon and Mann-Whitney tests, can be unambiguously identified by name only, but more complex techniques should be described in the methods section;
  - are tests one-sided or two-sided?
  - are there adjustments for multiple comparisons?
  - exact statistical test results, e.g., P values = x but not P values < x;
  - definition of 'center values' as median or average;
  - definition of error bars as s.d. or s.e.m.

Please complete ALL of the questions below.
Select "Not Applicable" only when the requested information is not relevant for your study.

Materials

Newly Created Materials	Information included in the manuscript?	In which section is the information available? (Reagents and Tools Table, Materials and Methods, Figures, Data Availability Section)
New materials and reagents need to be available; do any restrictions apply?	Not Applicable	

Antibodies	Information included in the manuscript?	In which section is the information available? (Reagents and Tools Table, Materials and Methods, Figures, Data Availability Section)
For **antibodies** provide the following information:<br>- Commercial antibodies: RRID (if possible) or supplier name, catalogue number and or/clone number<br>- Non-commercial: RRID or citation	Not Applicable	

DNA and RNA sequences	Information included in the manuscript?	In which section is the information available? (Reagents and Tools Table, Materials and Methods, Figures, Data Availability Section)
**Short novel DNA or RNA including primers, probes**: provide the sequences.	Not Applicable	

Cell materials	Information included in the manuscript?	In which section is the information available? (Reagents and Tools Table, Materials and Methods, Figures, Data Availability Section)
**Cell lines:** Provide species information, strain. Provide accession number in repository **OR** supplier name, catalog number, clone number, and/**OR** RRID.	Not Applicable	
**Primary cultures:** Provide species, strain, sex of origin, genetic modification status.	Not Applicable	
Report if the cell lines were recently **authenticated** (e.g., by STR profiling) and tested for mycoplasma contamination.	Not Applicable	

Experimental animals	Information included in the manuscript?	In which section is the information available? (Reagents and Tools Table, Materials and Methods, Figures, Data Availability Section)
**Laboratory animals or Model organisms:** Provide species, strain, sex, age, genetic modification status. Provide accession number in repository **OR** supplier name, catalog number, clone number, **OR** RRID.	Not Applicable	
**Animal observed in or captured from the field:** Provide species, sex, and age where possible**.**	Not Applicable	
Please detail **housing and husbandry conditions**.	Not Applicable	

Plants and microbes	Information included in the manuscript?	In which section is the information available? (Reagents and Tools Table, Materials and Methods, Figures, Data Availability Section)
**Plants:** provide species and strain, ecotype and cultivar where relevant, unique accession number if available, and source (including location for collected wild specimens).	Not Applicable	
**Microbes:** provide species and strain, unique accession number if available, and source.	Not Applicable	

Human research participants	Information included in the manuscript?	In which section is the information available? (Reagents and Tools Table, Materials and Methods, Figures, Data Availability Section)
If collected and within the bounds of privacy constraints report on age, sex and gender or ethnicity for all study participants.	Yes	Results Section<br>Material and Methods - Study design and ethical approval

Core facilities	Information included in the manuscript?	In which section is the information available? (Reagents and Tools Table, Materials and Methods, Figures, Data Availability Section)
If your work benefited from core facilities, was their service mentioned in the acknowledgments section?	Not Applicable	

Design

Study protocol	Information included in the manuscript?	In which section is the information available? (Reagents and Tools Table, Materials and Methods, Figures, Data Availability Section)
If study protocol has been **pre-registered, provide DOI in the manuscript**. For clinical trials, provide the trial registration number **OR** cite DOI.	Not Applicable	
Report the **clinical trial registration number** (at ClinicalTrials.gov or equivalent), where applicable.	Not Applicable	

Laboratory protocol	Information included in the manuscript?	In which section is the information available? (Reagents and Tools Table, Materials and Methods, Figures, Data Availability Section)
Provide DOI OR other citation details if **external detailed step-by-step protocols** are available.	Not Applicable	

Experimental study design and statistics	Information included in the manuscript?	In which section is the information available? (Reagents and Tools Table, Materials and Methods, Figures, Data Availability Section)
Include a statement about **sample size** estimate even if no statistical methods were used.	Yes	Results Section<br>Figure 1A
Were any steps taken to minimize the effects of subjective bias when allocating animals/samples to treatment (e.g. **randomization procedure**)? If yes, have they been described?	Yes	Material and Methods - Sample preparation
Include a statement about **blinding** even if no blinding was done.	Not Applicable	
Describe **inclusion/exclusion criteria** if samples or animals were excluded from the analysis. Were the criteria pre-established?<br><br>If sample or data points were omitted from analysis, report if this was due to attrition or intentional exclusion and provide justification.	Yes	Material and Methods - Processing of tissue samples
For every figure, are **statistical tests** justified as appropriate? Do the data meet the assumptions of the tests (e.g., normal distribution)? Describe any methods used to assess it. Is there an estimate of variation within each group of data? Is the variance similar between the groups that are being statistically compared?	Yes	Results Section - Figure Legends<br>Material and Methods - Bioinformatics data analysis

Sample definition and in-laboratory replication	Information included in the manuscript?	In which section is the information available? (Reagents and Tools Table, Materials and Methods, Figures, Data Availability Section)
In the figure legends: state number of times the experiment was **replicated** in laboratory.	Not Applicable	
In the figure legends: define whether data describe **technical or biological replicates**.	Not Applicable	

Ethics

Ethics	Information included in the manuscript?	In which section is the information available? (Reagents and Tools Table, Materials and Methods, Figures, Data Availability Section)
Studies involving **human participants**: State details of **authority granting ethics approval** (IRB or equivalent committee(s), provide reference number for approval.	Yes	Material and Methods - Study design and ethical approval
Studies involving **human participants**: Include a statement confirming that **informed consent** was obtained from all subjects and that the experiments conformed to the principles set out in the WMA Declaration of Helsinki and the Department of Health and Human Services Belmont Report.	Yes	Material and Methods - Study design and ethical approval
Studies involving **human participants: For** publication of **patient photos**, include a statement confirming that consent to publish was obtained.	Not Applicable	
Studies involving experimental **animals**: State details of **authority granting ethics approval** (IRB or equivalent committee(s), provide reference number for approval. Include a statement of compliance with ethical regulations.	Not Applicable	
Studies involving **specimen and field samples:** State if relevant **permits** obtained, provide details of authority approving study; if none were required, explain why.	Not Applicable	

Dual Use Research of Concern (DURC)	Information included in the manuscript?	In which section is the information available? (Reagents and Tools Table, Materials and Methods, Figures, Data Availability Section)
Could your study fall under dual use research restrictions? Please check biosecurity documents and list of **select agents and toxins** (CDC): https://www.selectagents.gov/sat/list.htm	Not Applicable	
If you used a select agent, is the security level of the lab appropriate and reported in the manuscript?	Not Applicable	
If a study is subject to dual use research of concern regulations, is the name of the **authority granting approval and reference number** for the regulatory approval provided in the manuscript?	Not Applicable	

Reporting

The MDAR framework recommends adoption of discipline-specific guidelines, established and endorsed through community initiatives. Journals have their own policy about requiring specific guidelines and recommendations to complement MDAR.

Adherence to community standards	Information included in the manuscript?	In which section is the information available? (Reagents and Tools Table, Materials and Methods, Figures, Data Availability Section)
State if relevant guidelines or checklists (e.g., **ICMJE, MIBBI, ARRIVE, PRISMA**) have been followed or provided.	Not Applicable	
For **tumor marker prognostic studies,** we recommend that you follow the **REMARK** reporting guidelines (see link list at top right). See author guidelines, under 'Reporting Guidelines'. Please confirm you have followed these guidelines.	Not Applicable	
For **phase II and III randomized controlled trials**, please refer to the **CONSORT** flow diagram (see link list at top right) and submit the CONSORT checklist (see link list at top right) with your submission. See author guidelines, under 'Reporting Guidelines'. Please confirm you have submitted this list.	Not Applicable	

Data Availability

Data availability	Information included in the manuscript?	In which section is the information available? (Reagents and Tools Table, Materials and Methods, Figures, Data Availability Section)
Have **primary datasets** been deposited according to the journal's guidelines (see 'Data Deposition' section) and the respective accession numbers provided in the Data Availability Section?	Yes	Material and Methods - Data availability
Were **human clinical and genomic datasets** deposited in a public access-controlled repository in accordance to ethical obligations to the patients and to the applicable consent agreement?	Yes	Material and Methods - Data availability
Are **computational models** that are central and integral to a study available without restrictions in a machine-readable form? Were the relevant accession numbers or links provided?	Not Applicable	
If publicly available data were reused, provide the respective **data citations in the reference list**.	Not Applicable	
